# A dual-host regulatory hub integrating biofilm formation and innate immunity resistance

**DOI:** 10.1038/s41467-026-77307-4

**Published:** 2026-09-01

**Authors:** Alexandre Baillez, Amélie Dewitte, François Pierre, Mounia Kortebi, Virginia S. Lioy, Mélanie Hillion, Julie Hardouin, Sébastien Janel, Xavier Thuru, Charlotte Clarisse, Hassiba Bouafia, Catherine Robbe Masselot, Sébastien Bontemps-Gallo, Florent Sebbane

**Affiliations:** 1 Univ. Lille, CNRS, Inserm, CHU Lille, Institut Pasteur Lille, U1019 - UMR 9017 - CIIL - Center for Infection and Immunity of Lille, F-59000 Lille, France; 2 Université Paris-Saclay, CEA, CNRS, Institute for Integrative Biology of the Cell (I2BC), F-91198 Gif-sur-Yvette, France; 3 Université Rouen Normandie, INSA Rouen Normandie, CNRS, Laboratoire Polymères, Biopolymères, Surfaces UMR-6270, F-76000 Rouen, France; 4 Normandie Université, INSERM US-51, CNRS UAR-2026, HeRacLeS-PISSARO, F-76000 Rouen, France; 5 Univ. Lille, Inserm, CHU Lille, CNRS, U1366-UMR9020 – CRCLille – Cancer Research Center of Lille, F-59000 Lille, France; 6 Univ. Lille, CNRS, UMR8576 - Unité de Glycobiologie Structurale et Fonctionnelle, F-59000 Lille, France

**Keywords:** Bacterial pathogenesis, Bacterial immune evasion, Pathogens, Bacterial physiology, Bacterial genes

## Abstract

Vector-borne pathogens must adapt to sharply distinct host environments, yet the regulatory logic that coordinates transitions between opposing host-specific programs remains poorly defined. Here, we identify a minimal regulatory module in the flea-borne pathogen *Yersinia pestis* that integrates vector transmission with resistance to mammalian innate immunity. Screening of flea-induced genes uncovered the regulator HdfR, which acts primarily through activation of *maoP*, encoding a nucleoid-associated protein. This HdfR-MaoP module promotes biofilm-dependent foregut blockage in the flea while coordinating baseline envelope adaptations that limit complement recognition and inducible responses that confer resistance to antimicrobial peptides under mammalian host-like conditions. Functional interchangeability of HdfR and MaoP homologs reveals evolutionary conservation of this regulatory logic, and pharmacological perturbation of the module sensitizes *Y. pestis* to antimicrobial peptides. Together, these findings define a parsimonious and evolutionarily conserved regulatory hub that orchestrates bacterial success across abrupt environmental transitions and exposes a tractable point of intervention.

## Abstract (web summary)

The plague bacterium, *Yersinia pestis*, is usually found in small mammals and their fleas. Here, Baillez et al. identify a regulatory pathway that enables the pathogen to adapt to its different hosts, and show that this pathway can be chemically inhibited.

## Introduction

Organisms that alternate between disparate environments must continually interpret external cues and reconfigure their physiology to maintain fitness^1^. This principle is exemplified by vector-borne pathogens, which face abrupt transitions in temperature, nutrient availability and immune pressure as they cycle between arthropods and mammals^2–4^. Such ecological discontinuities impose conflicting selective constraints that require coordinated regulatory solutions.

Among vector-borne pathogens, *Yersinia pestis*, the agent of flea-borne plague, undergoes an exceptionally extensive physiological transition^5,6^. Within its flea vector, *Y. pestis* assembles a cohesive biofilm that obstructs the foregut, ensuring regurgitative transmission of contaminated blood^7,8^. Yet this same biofilm becomes a liability in mammals^9^, where the bacterium must evade innate immunity to establish the bacteremia required for transmission back to naive fleas^10–12^. Thus, successful transmission depends on the precise deployment of opposing physiological programs across hosts. Intriguingly, passage through each host enhances fitness in the next, indicating that *Y. pestis* adjusts its physiology in ways that benefit sequential stages of its life cycle^8,13^. These observations suggest that host-specific programs are not deployed in isolation, but are instead coordinated across transitions (Supplementary Fig. 1). Yet the mechanisms that coordinate the opposing demands of flea colonization and mammalian infection remain unclear.

Decades of research have dissected the regulatory networks underpinning the flea-rodent-flea cycle of *Y. pestis*^14,15^. Key regulators include the two-component systems PhoPQ and OmpR-EnvZ, transcriptional regulators such as RovA and Crp, and post-transcriptional regulators like Hfq. These systems respond to general stress cues, such as antimicrobial peptides, osmotic changes, nutrient limitation or temperature shifts and collectively support survival in both flea and mammalian environments^16–21^. However, their regulatory outputs remain largely compartmentalized. For instance, PhoPQ modifies lipopolysaccharides to resist antimicrobial peptides but does not prime resistance to serum complement^16^, while RovA’s temperature-dependent switch toggles between biofilm repression and virulence without preparing bacteria for mammalian immune attack^18,22^. Despite decades of study on *Y. pestis*, no regulatory hub has been shown to directly couple vector transmission to mammalian immune evasion, although such integration would be predicted to provide a major fitness advantage during the flea-mammal transmission cycle.

Here, we bridge this gap by characterizing the HdfR-MaoP axis as a minimal regulatory hub that directly couples biofilm-mediated flea blockage with resistance to mammalian innate immunity. Through a genetic screen targeting flea-upregulated genes, complemented by in vitro analyses and flea infection models, we show that the LysR-type transcriptional regulator HdfR activates *maoP*, encoding a nucleoid-associated protein-like factor, to orchestrate this dual adaptation. Strikingly, this regulatory logic is evolutionarily conserved, as heterologous expression of *Escherichia coli* HdfR and MaoP fully rescues all tested phenotypes in *Y. pestis*. Together, these findings define a parsimonious molecular hub that prepares bacteria for abrupt host transitions and provides a mechanistic framework for understanding how pathogens navigate complex life cycles.

## Results

### The HdfR-MaoP axis coordinates biofilm formation and innate immune resistance across host environments

**Fig. 1:**
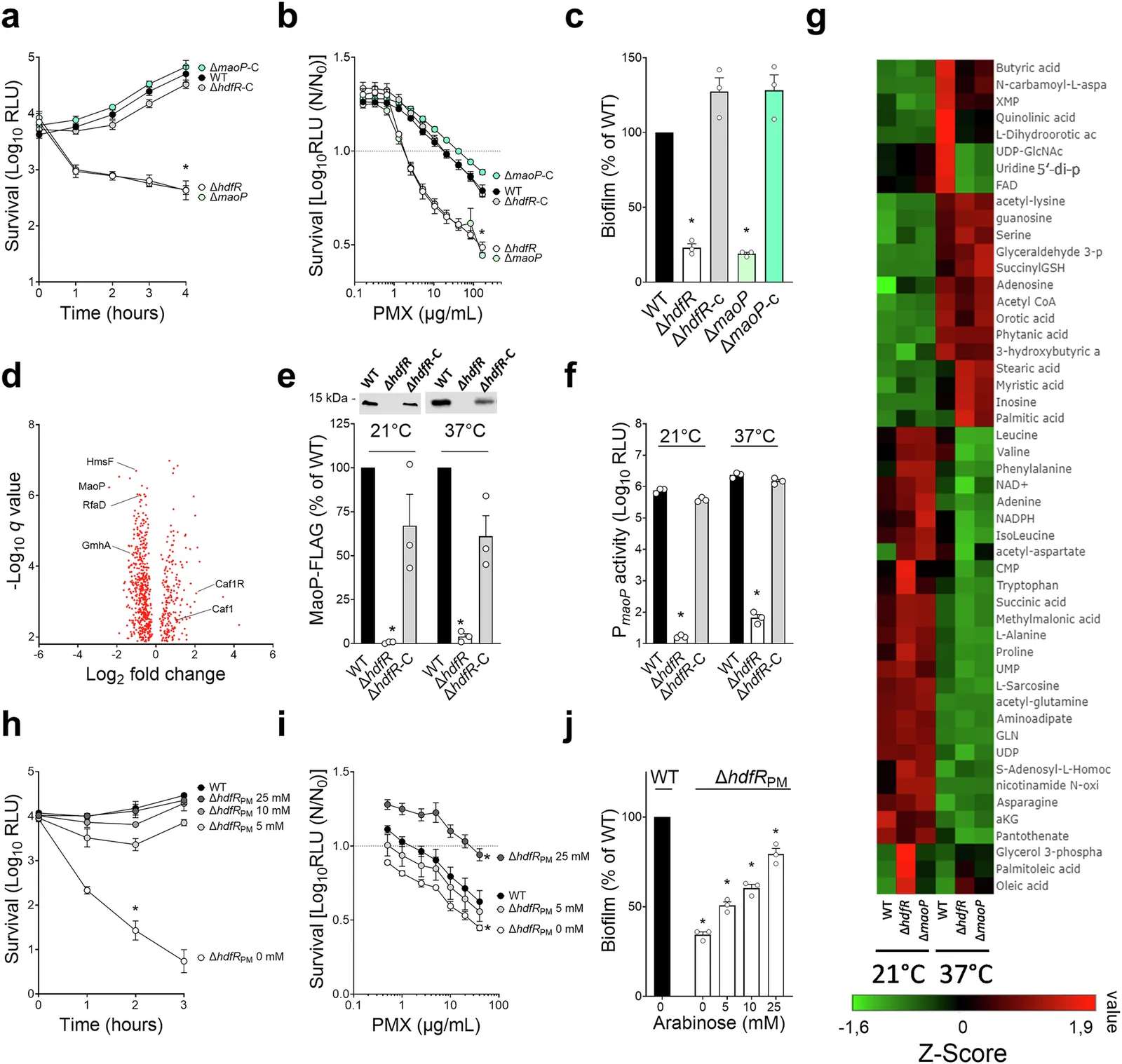
A hierarchical HdfR-MaoP module integrates biofilm formation with resistance to serum complement and antimicrobial peptides. **a**, **b** Mean ± SD survival of WT, ∆*hdfR*, ∆*maoP* and complemented (-c) strains after exposure to normal human serum (**a**) or polymyxin B (PMX) (**b**). Survival in serum **a** was quantified as Log_10_ relative light units (RLU) over time using bacteria grown at 21 °C, then exposed to serum at 37 °C. Survival to polymyxin B **b** was quantified as Log_10_ RLU (N/N_0_) using bacteria grown at 37 °C, then exposed to increasing concentrations of polymyxin B at 37 °C, where N corresponds to luminescence after exposure and N_0_ to luminescence before exposure. For **a**, exact n values were WT, Δ*hdfR*, Δ*hdfR*-C and Δ*maoP*: *n* = 4 from 0 to 3 h and *n* = 3 at 4 h; Δ*maoP*-C: *n* = 3 at all time points. For **b**, *n* = 3 for each strain and PMX concentration. **c** Mean ± SD biofilm formation after a 24-h incubation at 21 °C, expressed as a percentage relative to WT (*n* = 3 per strain). **d** A Volcano plot showing differentially produced proteins in ∆*hdfR* versus WT grown at 37 °C (*n* = 5 independent bacterial cultures per condition). Each dot represents one protein. Proteins selected for further analysis are highlighted. **e** An immunoblot of total protein extracts from WT, ∆*hdfR* and complemented strains expressing a functional MaoP-FLAG (Supplementary Fig. 4a, b), grown at 21 °C or 37 °C. Bars indicate mean ± SD MaoP-FLAG levels relative to WT, normalized to total protein by Ponceau staining (*n* = 3 independent immunoblot experiments). **f** Mean ± SD *maoP* promoter activity in WT, ∆*hdfR* and complemented strains carrying a chromosomal *maoP*-lux reporter and cultured at 21 °C or 37 °C, expressed as Log_10_ RLU (*n* = 3 per condition). **g** Heatmap of the 50 metabolites most significantly altered in ∆*hdfR* or ∆*maoP* strains cultured at 21 °C or 37 °C (*n* = 5 independent bacterial cultures per condition). Colors indicate Z-score-normalized relative abundance. **h**, **i** Same experimental setup as in (**a**, **b**), except that the ∆*hdfR* strain expresses *maoP* from an arabinose-inducible promoter (pBAD30-*maoP*-FLAG; _PM_) and bacteria were grown in the presence of increasing arabinose concentrations prior to serum (**h**) or polymyxin B (**i**) exposure; survival was quantified as in (**a**, **b**). For **h**, *n* = 3 except for Δ*hdfR*_PM_ 5 mM and 10 mM at 2 h and Δ*hdfR*_PM_ 0, 5, 10 and 25 mM at 3 h, where *n* = 2. Statistical comparisons in (**h**) were performed at 2 h for WT, Δ*hdfR*_PM_ 0 mM and Δ*hdfR*_PM_ 25 mM, for which *n* = 3 per condition. For **i**, *n* = 3 for each strain/arabinose concentration and PMX concentration. **j** Mean ± SD biofilm formation under the same conditions as in (**c**), except that the ∆*hdfR* strain expresses *maoP* from an arabinose-inducible promoter (pBAD30-*maoP*-FLAG; _PM_) and bacteria were grown in the presence of increasing arabinose concentrations (*n* = 3 per condition). For **a**–**c**, **f**, and **h**–**j**, n denotes independent biological experiments. For **e**, *n* denotes independent biological experiments analyzed by immunoblotting from independently prepared bacterial samples. For **d** and **g**, *n* denotes independent bacterial cultures. Asterisks denote statistically significant differences. For **a**, **b** and **i**, curve differences were assessed using two-way ANOVA followed by Tukey’s multiple comparisons test. For **c**, **e**, **f**, **h** and **j**, comparisons were assessed using ordinary one-way ANOVA followed by Dunnett’s multiple comparisons test. Exact adjusted *P* values for the comparisons indicated by asterisks were: **a** WT versus each mutant, Δ*hdfR* and Δ*maoP*, both *P* < 0.0001; **b** WT versus each mutant, Δ*hdfR* and Δ*maoP*, both *P* < 0.0001; **c** WT versus each mutant, Δ*hdfR* and Δ*maoP*, both *P* < 0.0001; **e** WT versus Δ*hdfR*, *P* < 0.0001 at both 21 °C and 37 °C; **f** WT versus Δ*hdfR*, *P* < 0.0001 at both 21 °C and 37 °C; **h** WT versus Δ*hdfR*_PM_ 0 mM, *P* = 0.001, and Δ*hdfR*_PM_ 25 mM vs Δ*hdfR*_PM_ 0 mM, *P* = 0.0018; **i** WT versus each Δ*hdfR*_PM_ condition, 0 and 25 mM, and Δ*hdfR*_PM_ 25 mM vs Δ*hdfR*_PM_ 0 mM, all *P* < 0.0001; and **j** WT versus Δ*hdfR*_PM_ condition (0, 5, 10 and 25 mM), and Δ*hdfR*_PM_ 25 mM vs Δ*hdfR*_PM_ 0 mM all *P* < 0.0001.

Vector-borne pathogens must reconcile the conflicting demands of persistence in the arthropod vector and survival in the mammalian host. To identify genes capable of supporting adaptation in both environments, we screened a library of *Y. pestis* deletion mutants targeting genes previously reported as upregulated in the flea for susceptibility to human serum. This screen identified twelve candidates, including a mutant spanning the *mlgA-araH10H11-hdfR* locus, which exhibited marked sensitivity to native but not heat-inactivated serum, consistent with complement-mediated killing. Focusing on the transcriptional regulator HdfR, we found that deletion of *hdfR* was sufficient to reproduce this phenotype when bacteria were exposed to serum at 37 °C after being grown under either flea-like (21 °C) or mammalian host-like (37 °C), indicating that HdfR supports complement resistance both after flea-like temperature adaptation and under mammalian host-like conditions (Fig. 1a and Supplementary Fig. 2a). By contrast, in cross-temperature antimicrobial peptide assays designed to distinguish prior temperature adaptation from exposure temperature, the Δ*hdfR* mutant displayed heightened susceptibility to antimicrobial peptides only after growth at 37 °C, regardless of the temperature used during peptide exposure (Fig. 1b and Supplementary Fig. 2b). Beyond these defects in innate immune resistance, the Δ*hdfR* mutant also exhibited reduced biofilm and exopolysaccharide production at 21 °C (Fig. 1c and Supplementary Fig. 2c). All defects were fully restored by genetic complementation, establishing HdfR as a pleiotropic regulator required for both vector-associated and mammalian-stage phenotypes (Fig. 1 and Supplementary Fig. 2).

Corroborating the pleiotropic role of HdfR and providing mechanistic insight, quantitative proteomic analyses performed at both 21 °C and 37 °C uncovered extensive HdfR-dependent changes in the protein landscape across temperatures, with 319 proteins differentially produced (Fig. 1d and Supplementary Fig. 2d). The majority of these proteins were less abundant in the Δ*hdfR* mutant, notably those involved in central metabolism and membrane architecture, with stronger effects observed at 37 °C (Supplementary Figs. 2d and 3). Despite this broad impact, inspection of the dataset suggested that HdfR primarily acts through the activation of a single downstream factor, MaoP. Indeed, *maoP* expression was abolished in the Δ*hdfR* mutant at both temperatures (Fig. 1e, f), and deletion of *maoP* phenocopied all Δ*hdfR* defects, including serum sensitivity, antimicrobial peptide susceptibility, impaired biofilm formation, and the associated metabolic signature (Fig. 1a-c, g). Consistent with this hierarchical regulatory architecture, titrated expression of *maoP* from an arabinose-inducible promoter in the Δ*hdfR* background restored all phenotypes in a dose-dependent manner (Fig. 1h–j and Supplementary Fig. 4c–e), whereas overexpression of *hdfR* failed to rescue the Δ*maoP* mutant (Supplementary Fig. 5a, b). These data establish MaoP as the indispensable downstream effector of the HdfR regulon for the phenotypes tested here. In addition, HdfR positively autoregulated its own transcription, whereas MaoP negatively autoregulated *maoP* expression (Supplementary Fig. 5c, d), revealing feedback loops consistent with a self-stabilizing regulatory module capable of maintaining coordinated output across environmental conditions. Consistent with the condition-dependent phenotypes controlled by the HdfR-MaoP module, *hdfR* expression was higher at 37 °C than at 21 °C (Supplementary Fig. 5e), estimated MaoP abundance was approximately threefold higher at 37 °C than at 21 °C on a per-cell basis, and shifting bacteria from 21 °C to 37 °C increased MaoP protein abundance over the first hour, whereas MaoP levels remained stable in bacteria maintained at 21 °C (Supplementary Fig. 5f, g).

Together, these results define the HdfR-MaoP module as a quantitative regulatory hub that integrates flea-stage biofilm capacity with mammalian-stage resistance to innate immune defenses, thereby contributing to bacterial adaptation across distinct host-associated conditions.

### HdfR-MaoP promotes flea blockage through a hierarchical regulatory cascade targeting the *hmsHFRS* operon

**Fig. 2:**
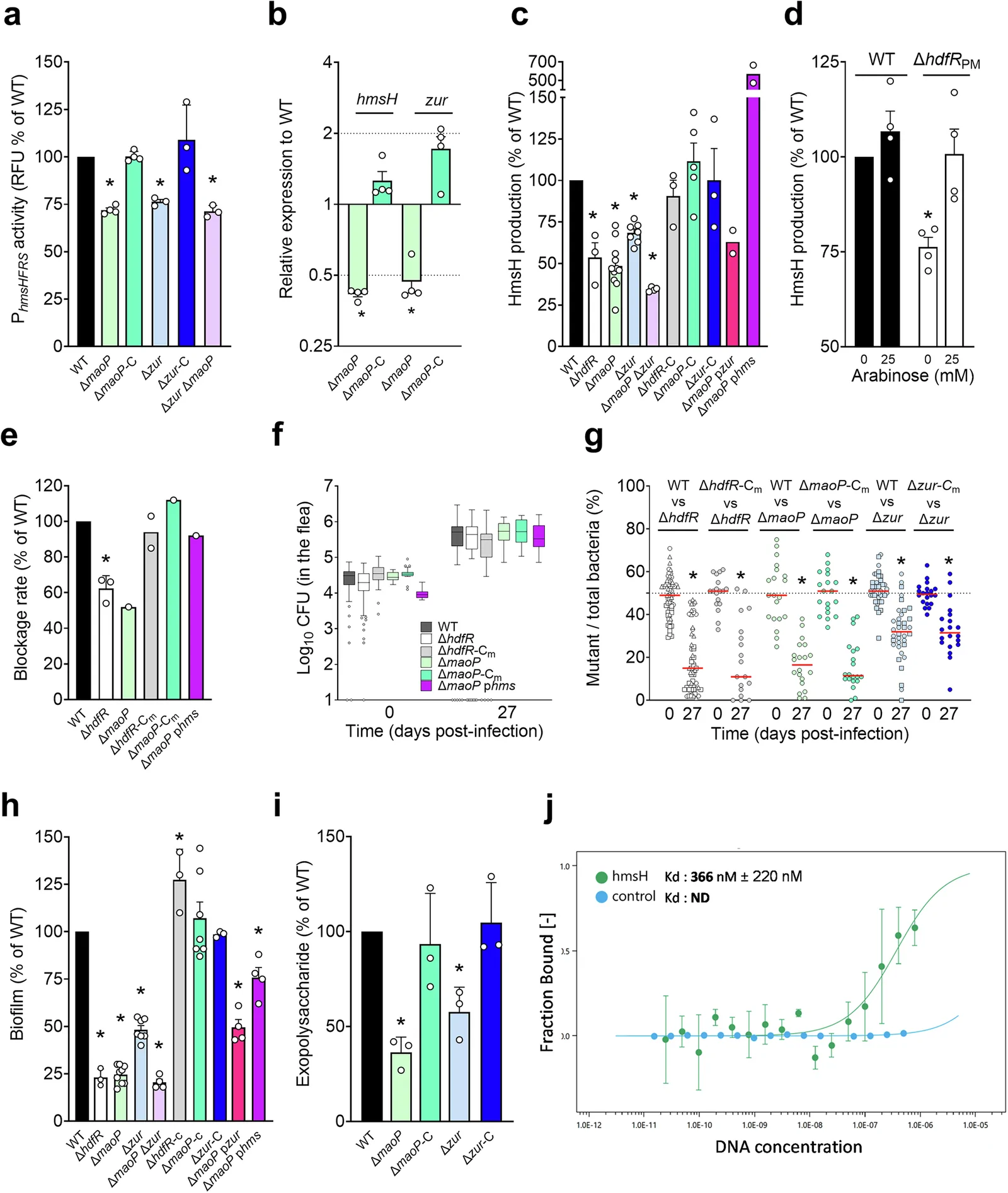
The HdfR-MaoP module controls flea gut blockage through Zur-dependent and -independent regulation of the *hmsHFRS* operon. **a** Mean ± SD *hmsHFRS* promoter activity in WT, ∆*maoP*, ∆*zur*, ∆*maoP* ∆*zur* and complemented (-c) strains cultured at 21 °C and carrying an *hmsHFRS*-*gfp* reporter, expressed as relative fluorescence units (RFU) relative to WT. Data are from *n* = 4 for WT, Δ*maoP* and Δ*maoP-*c and *n* = 3 for ∆*zur*, ∆*maoP* ∆*zur* and ∆*zur-*c. **b** Mean ± SD relative transcript levels of *hmsHFRS* and *zur*, measured by RT–qPCR, in ∆*maoP* and complemented strains grown at 21 °C, expressed relative to WT. Data are from *n* = 4 per strain. **C**, **D** Mean ± SD production of HmsH in ∆*hdfR*, ∆*maoP*, ∆*zur*, ∆*maoP* ∆*zur* and complemented strains, as well as in the ∆*maoP* strain expressing *hmsHFRS* (p*hms*) or *zur (*p*zur)* from a high-copy-number plasmid (**c**), or in the ∆*hdfR* strain expressing *maoP* from an arabinose-inducible promoter (pBAD30-*maoP*-FLAG; _PM_) and grown at 21 °C in the absence or presence of arabinose (25 mM) (**d**). HmsH levels, normalized to total protein by Coomassie blue staining and expressed relative to WT, were quantified by immunoblotting. For **c**, exact n values were: WT, *n* = 13; Δ*zur*, *n* = 7; Δ*zur*-C, *n* = 3; Δ*maoP*, *n* = 11; Δ*maoP*-C, *n* = 5; Δ*hdfR*, *n* = 3; Δ*hdfR*-C, *n* = 3; Δ*maoP* Δ*zur*, *n* = 4; Δ*maoP* p*zur*, *n* = 2; and Δ*maoP* p*hms*, *n* = 2. Conditions with *n* = 2 in (**c**) were excluded from statistical testing and are shown descriptively. For **d**, *n* = 4 independent immunoblot experiments per condition. **e** Mean blockage rate in fleas infected with ∆*hdfR*, ∆*maoP* and complemented strains (-c_m_) or with ∆*maoP* harboring p*hms*, measured over a 4-week period and expressed relative to WT. Mean ± SD is given only for the Δ*hdfR* mutant. Data are from *n* = 4 for WT, *n* = 3 for Δ*hdfR*, *n* = 2 for Δ*hdfR*-C_m_, and *n* = 1 for Δ*maoP*, Δ*maoP*-C_m_ and Δ*maoP* p*hms*. Conditions supported by fewer than three independent flea infection experiments were excluded from experiment-level statistical testing. **f** Box-and-whisker plots show bacterial loads (Log_10_ CFU) in fleas infected with WT, ∆*hdfR*, Δ*hdfR*-C_m_, ∆*maoP*, Δ*maoP*-C_m_ or Δ*maoP* p*hms* strains, measured immediately after infection (day 0) and 27 days post-infection. Each box-and-whisker plot summarizes bacterial loads measured in individual fleas, with n corresponding to the number of fleas analyzed. The center line indicates the median, box limits indicate the 25th and 75th percentiles, and whiskers extend to the most extreme values within 1.5× the interquartile range. Dots outside the whiskers indicate individual values outside this range. Data points on the x-axis corresponding to fleas with bacterial loads below the detection limit were plotted at the detection limit for visualization. Exact sample sizes, shown as day 0/day 27, were: WT, *n* = 98/79 fleas; Δ*hdfR*, *n* = 79/60 fleas; Δ*maoP*, *n* = 20/20 fleas; Δ*hdfR*-C_m_, *n* = 40/40 fleas; Δ*maoP*-C_m_, *n* = 20/20 fleas; and Δ*maoP* p*hms*, *n* = 20/20 fleas. These data were obtained from 5/4, 4/3, 2/2, 1/1, 1/1 and 1/1 independent flea infection experiments, respectively. **g** Competitive index assays in fleas co-infected with equal numbers of WT and mutant strains, or mutant and complemented strains. The proportion of the mutant strain relative to the total bacterial load is shown at day 0 and 27 days post-infection. Red lines indicate the median, and the dashed line marks the 50% value. Each dot represents an individual female flea; up to 20 female fleas were analyzed per experiment. Exact sample sizes, shown as day 0/day 27, were: WT versus Δ*hdfR*, *n* = 58/55 fleas; Δ*hdfR* versus Δ*hdfR*-Cm, *n* = 18/19 fleas; WT versus Δ*maoP*, *n* = 20/20 fleas; Δ*maoP* versus Δ*maoP*-Cm, *n* = 19/20 fleas; WT versus Δ*zur*, *n* = 40/36 fleas; and Δ*zur* versus Δ*zur*-Cm, *n* = 20/20 fleas. These data were obtained from 3, 1, 1, 1, 2 and 1 independent co-infection experiments, respectively. Independent co-infection experiments are indicated by distinct symbols. **h** Mean ± SD biofilm formation by WT, ∆*hdfR*, ∆*maoP*, ∆*zur*, ∆*maoP*∆*zur* and complemented strains, as well as by ∆*maoP* strains harboring p*zur* or p*hms*, after a 24-h incubation at 21 °C, expressed as a percentage relative to WT. Exact *n* values were: WT, *n* = 9; Δ*hdfR*, *n* = 3; Δ*maoP*, *n* = 9; Δ*zur*, *n* = 7; Δ*maoP* Δ*zur*, *n* = 4; Δ*hdfR*-c, *n* = 3; Δ*maoP*-c, *n* = 7; Δ*zur*-C, *n* = 3; Δ*maoP* pzur, *n* = 4; and Δ*maoP* phms, *n* = 4. **I** Mean ± SD Congo red binding, used as a proxy for exopolysaccharide production, of the WT, ∆*maoP*, ∆*zur* and complemented (-c) strains, expressed as a percentage relative to WT (*n* = 3 per strain). **j** Binding of Zur to the *hmsH* promoter measured by microscale thermophoresis. The fraction bound is plotted as a function of DNA concentration. Data points represent mean ± SD from independent measurements, *n* = 3 for Zur binding to the *hmsH* promoter, *n* = 2 for Zur binding to a non-specific control DNA fragment. Solid lines indicate fits to a single-site binding model. The apparent dissociation constant (Kd) for Zur binding to *hmsH* is indicated. A non-specific DNA fragment was used as a negative control (Kd not determined). Here, n denotes independent biological experiments for (**a**, **b**, **h** and **i**); independent biological experiments analyzed by immunoblotting from independently prepared bacterial samples for (**c**, **d**); independent flea infection experiments for (**e**); individual fleas for (**f**, **g**), with the number of independent flea infection experiments indicated separately; and independent MST measurements for (**j**). Asterisks denote statistically significant differences. For **a**, **c**, **h** and **i**, comparisons were assessed using ordinary one-way ANOVA followed by Dunnett’s multiple comparisons test. For **d**, comparisons were assessed using ordinary one-way ANOVA followed by Tukey’s multiple comparisons test. For **b** and **e**, comparisons were assessed using a two-sided one-sample t-test against the WT-normalized value of 1 or 100%, respectively. For **g**, day 0 and day 27 distributions were compared using a two-sided Mann-Whitney U test, with individual fleas as the statistical unit. The *P* values for the comparisons indicated by asterisks were: **a** WT versus Δ*maoP*, *P* = 0.0002, WT versus Δ*zur*, *P* = 0.0022, and WT versus Δ*maoP* Δ*zur*, *P* = 0.0004; **b** Δ*maoP* versus the WT-normalized value, *P* < 0.0001 for *hmsH*; and Δ*maoP* versus the WT-normalized value, *P* = 0.0017 for *zur*; **c** WT versus Δ*zur*, *P* = 0.0001, and WT versus each Δ*maoP*, Δ*hdfR* and Δ*maoP* Δ*zur*, all *P* < 0.0001; **d** WT 0 mM versus Δ*hdfR*_PM_ 0 mM, *P* = 0.0122, and Δ*hdfR*_PM_ 25 mM versus Δ*hdfR*_PM_ 0 mM, *P* = 0.0099; **e** Δ*hdfR* versus the WT-normalized value of 100%, *P* = 0.0121; **g** day 0 versus day 27 for each co-infection pair, all *P* < 0.0001; **h** WT versus each Δ*hdfR*, Δ*maoP*, Δ*zur*, Δ*maoP* Δ*zur* and Δ*maoP* pzur condition, all *P* < 0.0001; WT versus Δ*hdfR*-c, *P* = 0.0023; and WT versus Δ*maoP* phms, *P* = 0.0027 and **i** WT versus Δ*maoP*, *P* = 0.0031, and WT versus Δ*zur*, *P* = 0.0354.

Proteomic analyses revealed that HmsF, encoded within the *hmsHFRS* biofilm operon, was markedly less abundant in the absence of the HdfR-MaoP module (Fig. 1d), supporting a role for this regulatory axis in controlling the biofilm machinery required for efficient flea foregut obstruction^7,8^. Consistent with this hypothesis, deletion of either *hdfR* or *maoP* markedly reduced *hmsHFRS* promoter activity and HmsH protein accumulation under flea-like conditions (21 °C) (Fig. 2a–c). Ectopic expression of *maoP* in the Δ*hdfR* background fully restored HmsH production, establishing MaoP as the direct mediator of HdfR-dependent activation of biofilm genes (Fig. 2d and Supplementary Fig. 4f). At the biological level, these molecular defects translated into a reduced ability to produce a transmissible infection. Both Δ*hdfR* and Δ*maoP* mutants blocked fewer fleas than the wild type in mono-infections (Fig. 2e), despite comparable bacterial loads in the flea gut (Fig. 2f), and were strongly outcompeted in 1:1 co-infection assays (Fig. 2g). Importantly, restoration of wild-type *hmsHFRS* expression in the Δ*maoP* background rescued biofilm formation (Fig. 2h) and flea blockage to near wild-type levels (Fig. 2e). Together, these findings demonstrate that HdfR-MaoP sits at the top of a regulatory cascade controlling biofilm formation through the *hmsHFRS* operon.

Further dissection of this regulatory cascade examined how MaoP controls *hmsHFRS* expression. Among genes downregulated in the Δ*hdfR* background, the zinc-uptake regulator *zur* emerged as a key intermediary. The transcription of *zur* was reduced in the absence of MaoP (Fig. 2b), and purified Zur bound directly to the *hmsH* promoter (Fig. 2j). Consistently, deletion of *zur* reduced *hmsHFRS* transcription to levels comparable to those observed in Δ*maoP* (Fig. 2a). By contrast, the resulting defects in biofilm formation, exopolysaccharide production, and flea fitness were less severe in the Δ*zur* mutant (Fig. 2g–i). This divergence became most apparent in the Δ*maoP* Δ*zur* double mutant, which exhibited the lowest levels of HmsH accumulation and biofilm production (Fig. 2c, h). Furthermore, overexpression of *zur* in the Δ*maoP* background partially restored HmsH levels and biofilm formation (Fig. 2c, h). Together, these data reveal a hierarchical regulatory architecture in which MaoP activates *hmsHFRS* through parallel Zur-dependent and Zur-independent pathways, ensuring robust biofilm production and reliable flea blockage across fluctuating environmental conditions.

Small RNAs have been reported to activate translation of *hmsHFRS* and *hmsT*, which encodes a diguanylate cyclase that produces c-di-GMP required for HmsHFRS activity, providing a plausible molecular basis for the Zur-independent branch^23–25^. However, c-di-GMP levels were unchanged in the Δ*hdfR* mutant, and deletion of *maoP* did not reduce expression of the tested sRNA genes (Supplementary Fig. 6). The mechanism underlying the Zur-independent regulation of *hmsHFRS* therefore remains to be defined.

### Distinct envelope-associated mechanisms underlie antimicrobial peptide and complement resistance

**Fig. 3:**
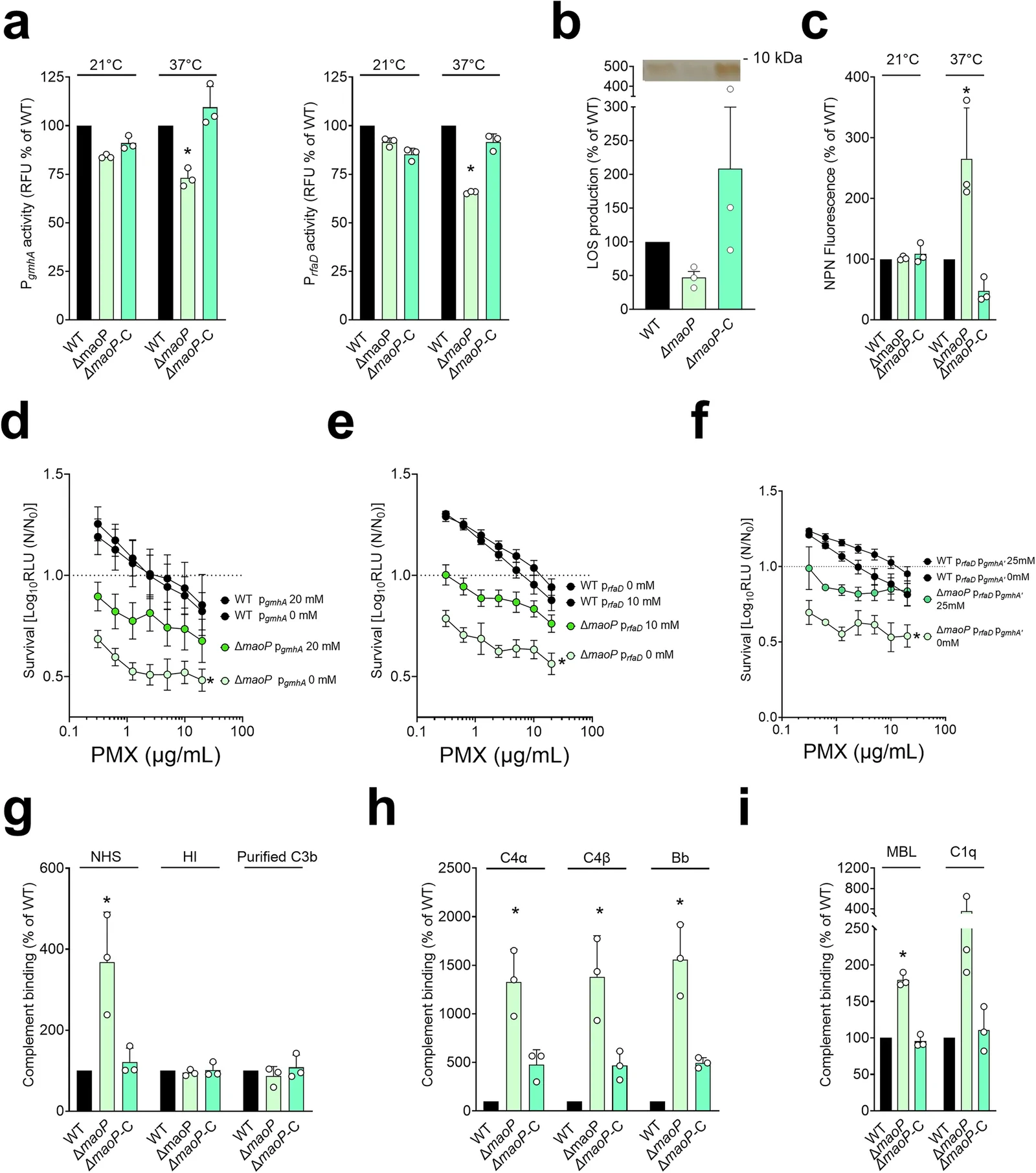
MaoP modulates outer membrane properties to resist antimicrobial peptides and complement-mediated killing. **a** Mean ± SD *gmhA* and *rfaD* promoter activity in WT, ∆*maoP* and complemented (-c) strains cultured at 21 °C or 37 °C and carrying *gmhA*- or *rfaD*-*gfp* reporters, expressed as relative fluorescence units relative to WT. Equivalent numbers of bacteria were used per condition (*n* = 3 per strain and condition). **b** DOC-PAGE analysis of lipooligosaccharide (LOS) from WT, ∆*maoP* and complemented (-c) strains grown at 37°C. Equal amounts of bacterial material were loaded. Bars indicate the mean ± SD relative intensity of the Lipid A band relative to WT (*n* = 3 per strain). **c** Mean ± SD fluorescence intensity of WT, ∆*maoP* and complemented (-c) strains grown at 21 °C or 37 °C after a 30-min exposure to N-phenyl-1-naphthylamine (NPN) and relative to WT. Equivalent numbers of bacteria were used per condition (*n* = 3 per strain and condition). **d–f** Mean ± SD survival of WT and ∆*maoP* strains expressing or not *gmhA* **d**, *rfaD* **e** or both *gmhA* and *rfaD* **f** from an arabinose-inducible promoter (pBAD30’-*gmhA*; p_*gmhA*_’ or pBAD30-*rfaD*; p_*rfaD*_), grown at 37 °C in the presence of increasing arabinose concentrations and then exposed at 37 °C to increasing concentrations of polymyxin B for 180 min. Survival was quantified as Log_10_ RLU (N/N_0_), where N corresponds to luminescence after exposure and N_0_ to luminescence before exposure. Data are from *n* = 4 for all strains and conditions, except for WT p*gmhA* 0 and 20 mM arabinose, for which *n* = 3. **g–i** Immunoblot analysis of complement components bound to WT, ∆*maoP* and complemented strains grown at 21 °C and after a 60-min incubation at 37 °C. Panel G shows C3b binding after incubation with normal (NHS) or heat-inactivated (HI) serum, or purified C3b. Panels H and I show C4α, C4β, Bb, mannose-binding lectin (MBL), and C1q binding after incubation with NHS. Bars indicate the mean ± SD relative to WT band intensity. Data are shown as mean ± SD from three independent experiments. Here, *n* denotes independent biological experiments for (**a**–**f**) and independent biological experiments analyzed by immunoblotting from independently prepared bacterial samples for (**g**–**i**). Asterisks denote statistically significant differences. For **a**, **c**, **g**, **h** and **i**, comparisons were assessed using ordinary one-way ANOVA followed by Dunnett’s multiple comparisons test with WT as the control, separately for each temperature, promoter, serum condition or complement component, as appropriate. For **d**–**f**, survival curves across polymyxin B concentrations were compared using two-way ANOVA followed by Dunnett’s multiple comparisons test. The P values for the comparisons indicated by asterisks were: **a** WT versus Δ*maoP* at 37 °C, *P* = 0.0049 for *gmhA* promoter activity and *P* < 0.0001 for *rfaD* promoter activity; **c** WT versus Δ*maoP* at 37°C, *P* = 0.0117; **d**–**f** Δ*maoP* strain carrying the indicated plasmid construct and grown without arabinose (0 mM) versus the corresponding WT strains grown without or with arabinose, and versus the Δ*maoP* strain carrying the indicated plasmid construct and grown with the indicated arabinose concentration; all *P* < 0.0001; **g** WT versus Δ*maoP* after incubation with NHS, *P* = 0.0079; **h** WT versus Δ*maoP*, *P* = 0.0008 for C4α, *P* = 0.0017 for C4β and *P* = 0.0003 for Bb; and **i**, WT versus Δ*maoP* for MBL binding, *P* < 0.0001.

Proteomic analyses of the Δ*hdfR* mutant revealed reduced abundance of enzymes involved in core lipopolysaccharide (LOS) biosynthesis, namely GmhA and RfaD (Fig. 1d), suggesting a potential contribution of the HdfR-MaoP module to antimicrobial peptide resistance via envelope-associated mechanisms^26,27^. In agreement with these proteomic data, LOS analysis of the Δ*hdfR* mutant showed reduced LOS production at 37 °C but not at 21 °C (Supplementary Fig. 7a). Consistent with MaoP acting downstream of HdfR, deletion of *maoP* resulted in a reduction in *gmhA* and *rfaD* promoter activity at 37 °C (Fig. 3a), correlating with decreased LOS production (Fig. 3b) and increased outer membrane permeability (Fig. 3c). Strikingly, these defects were not detected at 21 °C, mirroring the temperature dependence of polymyxin sensitivity in the Δ*maoP* mutant (Fig. 3a–c). In support of a role for HdfR-MaoP-dependent regulation of *gmhA* and *rfaD* in peptide resistance, ectopic expression of either gene in the Δ*maoP* mutant restored polymyxin resistance, albeit partially (Fig. 3d, e). Consistent with the coordinated contribution of both enzymes to LOS core biosynthesis, combined expression of *gmhA* and *rfaD* further restored polymyxin resistance, most notably at higher polymyxin concentrations (Fig. 3f). Inspection of the remaining LOS and lipid A biosynthesis genes revealed no reduced expression in Δ*maoP* (Supplementary Fig. 7b, c). In addition, canonical lipid A modification pathways associated with polymyxin resistance were not transcriptionally reduced, including the *pmrF* operon and upstream genes involved in aminoarabinose modification, whereas *eptA* was increased (Supplementary Fig. 7b). These data support the conclusion that peptide sensitivity stems from impaired LOS core maturation through reduced GmhA and RfaD output rather than from a broader disruption of LPS assembly or reduced expression of canonical lipid A modification pathways.

By contrast, changes in the regulation of LOS biosynthesis pathways were unlikely to fully account for the complement sensitivity of HdfR-MaoP-deficient strains, because complement sensitivity was observed after growth at both 21 °C and 37 °C, whereas reduced *gmhA*/*rfaD* expression and detectable LOS defects were observed primarily at 37 °C. Mechanistic dissection of the complement activation system showed increased deposition of C3b, C4 fragments, and factor Bb on the surface of the Δ*maoP* mutant in normal human serum (Fig. 3g, h), while recruitment of complement inhibitors, including vitronectin, factor H, and C4-binding protein, was comparable to wild-type (Supplementary Fig. 7d). Moreover, exposure to purified C3b or heat-inactivated serum did not increase C3b deposition, excluding enhanced docking of alternative pathway components as the primary cause of complement sensitivity (Fig. 3g). Instead, the Δ*maoP* mutant exhibited increased binding of mannose-binding lectin and C1q (Fig. 3i), implicating heightened recognition by the lectin and classical pathways. To identify potential determinants of this increased recognition, we screened 27 candidate surface-associated genes selected from our omics datasets. None of the corresponding mutants individually recapitulated the complement sensitivity phenotype, supporting a distributed effect on surface ligand exposure rather than masking of one dominant determinant (Supplementary Fig. 7e).

Together, these findings indicate that the HdfR-MaoP module supports innate immune resistance through distinct envelope adaptation strategies. It reinforces LOS biosynthesis at mammalian host temperature to limit antimicrobial peptide susceptibility, while restricting complement activation through a distinct mechanism that limits recognition by lectin and classical pathway sensors. Although subtle or localized LOS alterations cannot be excluded, this dual control ensures continuity of vector-stage transmission while enabling survival in the mammalian host.

### Plasmid-borne virulence-associated genes are integrated into the HdfR-MaoP regulatory network

Proteomic analyses of the Δ*hdfR* mutant revealed increased abundance of Caf1R and Caf1 (Fig. 1d), both encoded on the pMT1 plasmid. Caf1R activates the *caf1M1A1* operon at mammalian host temperature, leading to production of the F1 capsule, a major *Y. pestis* virulence factor^28,29^. Consistent with the hierarchical organization of the HdfR-MaoP module, deletion of *maoP* increased Caf1 production under the conditions tested, suggesting that HdfR’s effect on Caf1 abundance is mediated, at least in part, by MaoP (Supplementary Fig. 8a). This effect did not extend to all plasmid-encoded virulence factors, as production of the pPla-encoded virulence factor Pla was unchanged in the Δ*maoP* mutant (Supplementary Fig. 8b). The potential impact of HdfR-MaoP on the virulence pCD1-encoded functions could not be assessed because the strain used in this study lacks this plasmid. Altogether, these data indicate that HdfR-MaoP regulation can extend to specific plasmid-borne functions, such as Caf1 production on pMT1, suggesting that horizontally acquired elements can be integrated into host-associated regulatory networks governed by this module.

### MaoP functions as a nucleoid-associated protein with broad DNA-binding activity

**Fig. 4:**
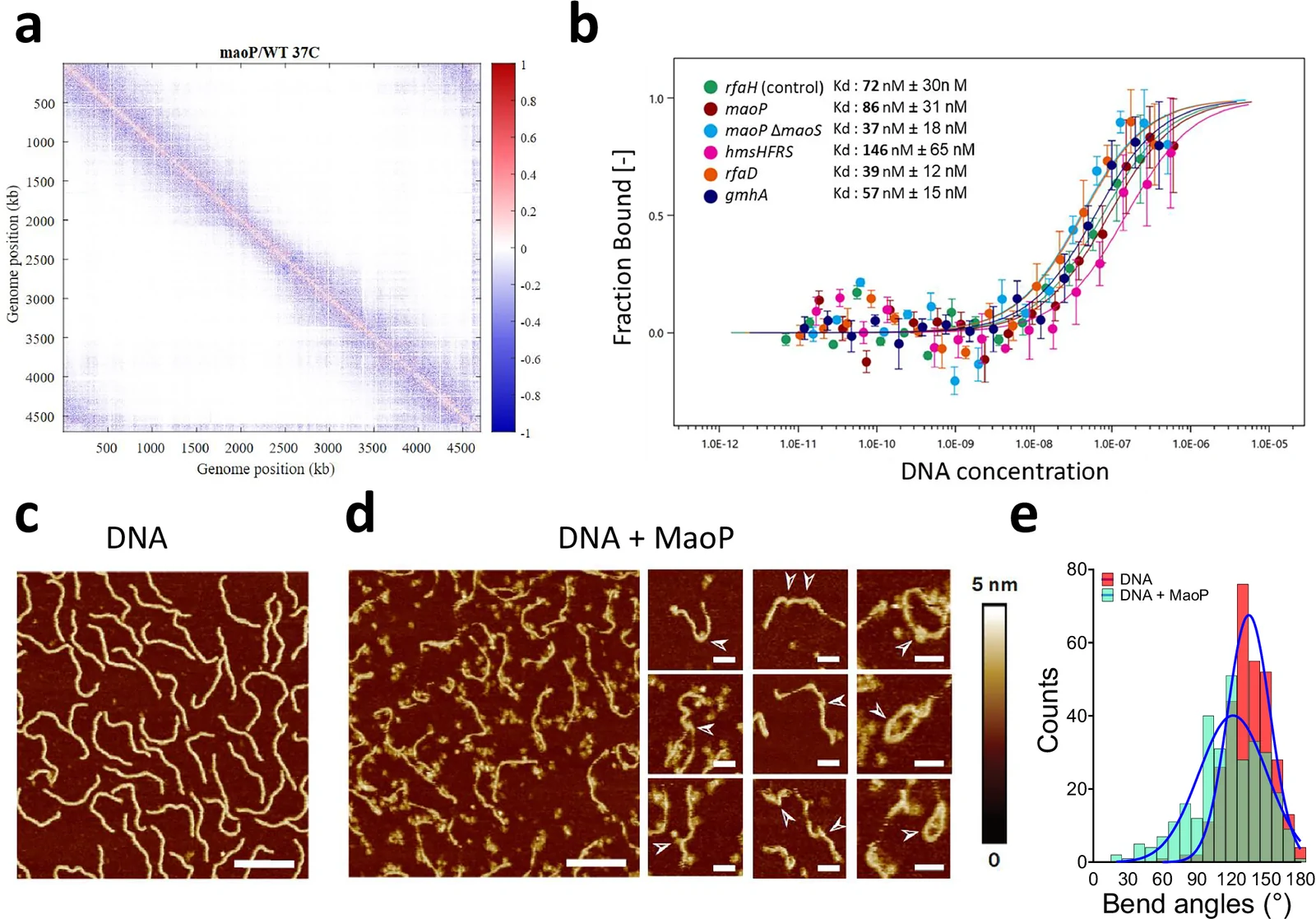
MaoP exhibits properties consistent with a nucleoid-associated protein. **a** Ratio of normalized Hi-C contact maps for ∆*maoP* ∆*pla* and WT ∆*pla* grown at 37 °C. A decrease or increase in contacts in ∆*maoP* ∆*pla* cells compared with WT ∆*pla* cells is represented with a blue or red color, respectively. White indicates no differences between the strains. No major changes in chromosome conformation were observed under the conditions tested. **b** DNA binding of MaoP assessed by MST using promoter fragments from MaoP-regulated and non-regulated genes. The fraction bound is plotted as a function of DNA concentration. Data points represent mean ± SD from independent measurements, with *n* = 3 per DNA fragment except for *maoP* and *hmsHFRS*, for which *n* = 4. Solid lines indicate fits to a single-site binding model. The apparent dissociation constant (Kd) is indicated. **c, d** AFM imaging of linear DNA molecules (395-bp) in the absence (**c**) or presence (**d**) of MaoP. Compared to DNA molecules alone (**c**), DNA molecules in the presence of MaoP (**d**) exhibit changes in conformation. Arrows highlight representative regions of altered DNA conformation. Overview images and representative zoomed views are shown. Scale bars, 100 nm (overview images) and 25 nm (zoomed views). MaoP molecules are not directly resolved under these conditions. **e** Distribution and Gaussian fitting (blue curves) of bend angles of the linear DNA molecules in the absence (red, *n* = 314) or presence (green, *n* = 300) of MaoP.

Previous studies in *E. coli* have proposed diverse functions for MaoP, including interactions with RNA polymerase, RNA binding, or organization of the Ori region through the MaoS site^30–32^. We therefore systematically evaluated these possibilities. Under the experimental conditions tested, MaoP did not interact with RNA polymerase in bacterial two-hybrid assays (RpoN, RpoB, RpoZ, RpoS, RpoE, RpoD) (Supplementary Fig. 9a), did not enrich for RNA above background in RIP experiments, and did not induce significant changes in local or global chromosome folding in vivo, as assessed by Hi-C contact maps and contact-probability decay as a function of genomic distance (Fig. 4a and Supplementary Fig. 9b). Deletion of the MaoS site, located in the intergenic region between *hdfR* and *maoP*, abolished endogenous *maoP* transcription and translation without affecting *hdfR* transcription (Supplementary Fig. 10a, b) and phenocopied the Δ*maoP* mutant (Supplementary Fig. 10c–e). However, the MaoS site was not required for MaoP-dependent phenotypes once *maoP* expression was restored, as expression of *maoP* from an inducible promoter in a ΔMaoS site background fully restored resistance to antimicrobial peptides and serum, as well as biofilm formation (Supplementary Fig. 10f–k). Mutation of the ribosome-binding site phenocopied the Δ*maoP* mutant, demonstrating that MaoP-dependent phenotypes are mediated by the protein product rather than by a non-coding RNA (Supplementary Fig. 11). MaoP is a small (14-kDa), non-highly basic DNA-binding protein that ranks among the top 150 most abundant proteins detected in our proteomic analysis, with an estimated abundance of approximately 1.4 × 10^4^ and 4.4 × 10^4^ molecules per cell at 21 °C and 37 °C respectively (Supplementary Fig. 5f and Supplementary Data 1). ChIP-seq revealed MaoP occupancy at discrete promoter regions, including its own locus, the zinc uptake regulator *zur*, the biofilm operon *hmsHFRS* and the pMT1-borne *caf1R*-*caf1M1A1* region (Supplementary Data 2). However, microscale thermophoresis measurements demonstrated that MaoP binds DNA without detectable sequence specificity under the conditions tested (Fig. 4b). In vitro, this sequence-agnostic DNA-binding activity correlated with local changes in DNA topology, as visualized by atomic force microscopy (Fig. 4c–e).

Together, these findings support the designation of MaoP as a nucleoid-associated protein involved in modulating local DNA conformation and transcriptional outputs, with in vivo occupancy likely shaped by local chromosomal context rather than strict sequence-specific recruitment.

### The HdfR-MaoP regulatory axis is functionally conserved across Enterobacterales

Comparative genomic analyses revealed that the *hdfR*-*maoP* locus is broadly conserved across Enterobacterales, being present in hundreds of sequenced genomes spanning pathogenic, commensal, and environmental species associated with terrestrial and aquatic habitats (Supplementary Fig. 12a, b). Despite substantial sequence divergence outside conserved domains, alignment of HdfR homologs from *Y. pestis* and *E.coli* showed that 85% of identical residues cluster within the predicted DNA-binding domain (Supplementary Fig. 12a), consistent with conservation of regulatory function. To assess whether this conservation extends to functional interchangeability, *E. coli hdfR* and *maoP* were expressed in the corresponding *Y. pestis* deletion mutants. Despite only ~60% (HdfR) and ~90% (MaoP) overall amino acid identity, heterologous expression of either gene fully restored resistance to serum complement and antimicrobial peptides, as well as biofilm formation, to wild-type levels (Supplementary Fig. 12c–h). Conversely, deletion of *hdfR* or *maoP* in *E. coli* impaired biofilm formation, and expression of the corresponding *Y. pestis* homolog restored this defect, indicating reciprocal functional conservation for this shared biofilm-associated output (Supplementary Fig. 12i). These findings indicate that the HdfR-MaoP module constitutes an evolutionarily conserved regulatory unit, with conserved genomic organization, functional interchangeability, and shared biofilm-associated regulatory activity, while immune-resistance outputs may reflect species- and niche-specific deployment of this regulatory scaffold.

### Pharmacological perturbation of the HdfR-MaoP regulatory module

**Fig. 5:**
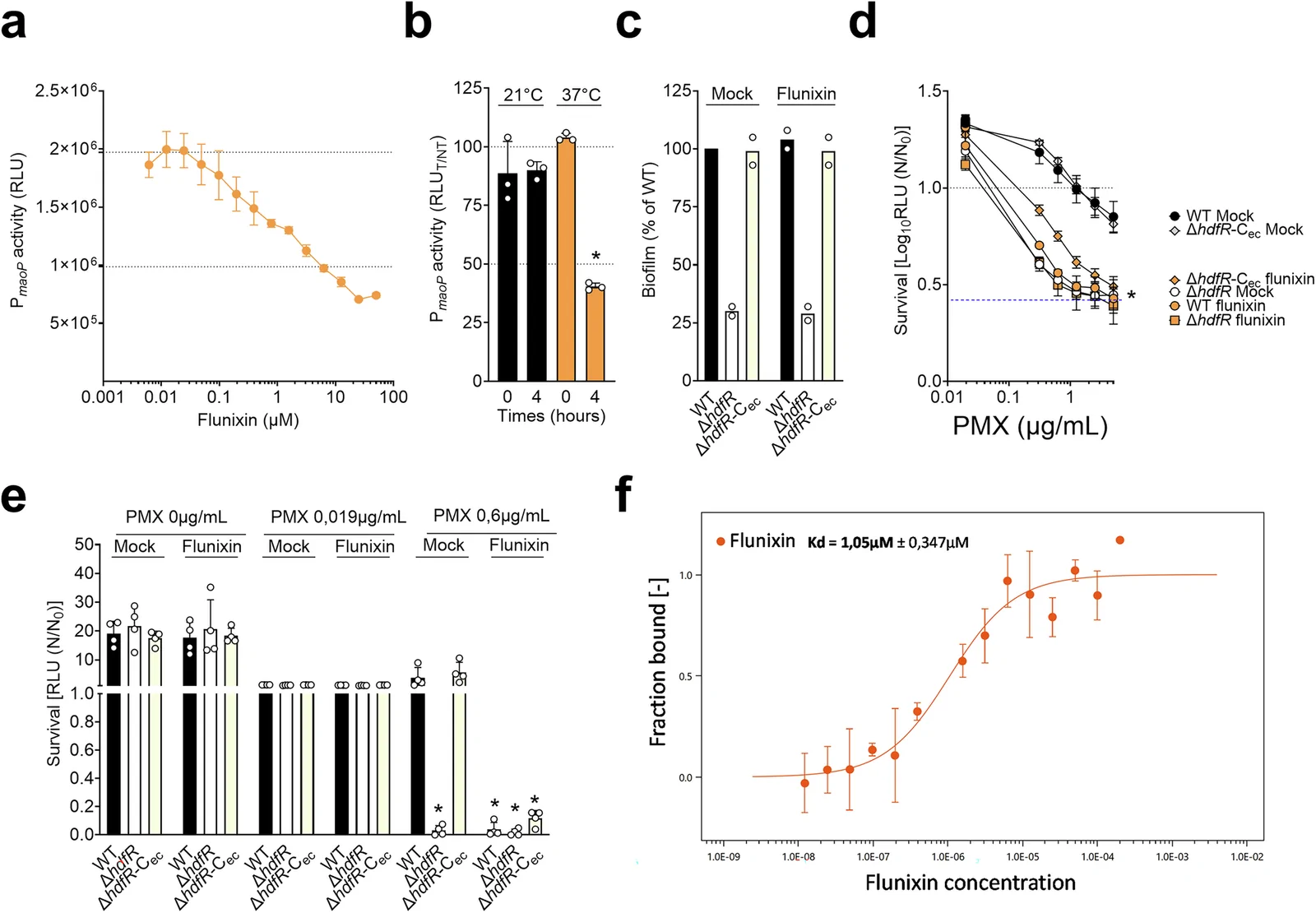
Pharmacological inhibition of the HdfR-MaoP module by flunixin sensitizes *Y. pestis* to antimicrobial peptides. **a**, **b** Mean ± SD *maoP* promoter activity in the *Y. pestis* wild-type strain carrying a chromosomal P_*maoP*_-lux reporter after a 4 h incubation at 37 °C in the presence of increasing concentrations of flunixin (*n* = 3 for each concentration) (**a**) or after 4 h at 21 °C or 37 °C in the presence of 25 µM flunixin (*n* = 3 for each temperature and time point) (**b**), expressed as relative luminescence units (RLU), normalized to OD_595_. Data are from *n* = 3 independent biological experiments. **c** Mean biofilm formation after a 24-h incubation at 21 °C, expressed as a percentage relative to WT in the absence (Mock) or presence of 25 µM flunixin (*n* = 2 per strain and condition). **d**, **e** Mean ± SD survival of WT, ∆*hdfR* and Δ*hdfR* complemented with 6xHis-tagged *hdfR* from *E. coli* (-c_Ec_) strains after exposure to flunixin (25 µM) and increasing concentrations of polymyxin B (**d**) or fixed concentrations of polymyxin B, 0 µg/mL, 0.019 µg/mL or 0.6 µg/mL (**e**), alone or in combination, in the presence of the DMSO vehicle control (Mock) (*n* = 4 per strain and condition). Survival was quantified as Log_10_ RLU (N/N_0_) (D) or RLU (N/N_0_) (**e**) using bacteria grown and exposed at 37 °C, where N corresponds to luminescence after exposure and N_0_ to luminescence before exposure. **f** Flunixin binding assessed by MST using purified HdfR. Fraction bound is plotted as a function of flunixin concentration. Data points represent mean ± SD from *n* = 3 independent measurements, and solid lines indicate fits to a single-site binding model. The apparent dissociation constant (Kd) is indicated. Here, n denotes independent biological experiments for (**a**–**e**) and independent MST measurements for (**f**). Asterisks denote statistically significant differences. For (B), promoter activity at 0 and 4 h was compared within each temperature using two-way ANOVA followed by Šídák’s multiple comparisons test. For **d**, survival curves across polymyxin B concentrations were compared using two-way ANOVA followed by Tukey’s multiple comparisons test for the indicated curve comparisons. For **e**, fixed-concentration survival values were compared for each strain using two-way ANOVA followed by Šídák’s multiple comparisons test, with polymyxin B concentration and flunixin treatment as factors. The *P* values for the comparisons indicated by asterisks were: **b** 0 h versus 4 h at 37°C, *P* < 0.0001; **d** WT Mock versus WT flunixin, Δ*hdfR* Mock, Δ*hdfR* flunixin and Δ*hdfR*-cEc flunixin, all *P* < 0.0001; **e** at 0.6 µg/mL polymyxin B, WT (Mock) versus Δ*hdfR* (Mock), *P* = 0.0015; at 0.6 µg/mL polymyxin B, WT (Mock) vs WT, Δ*hdfR*, Δ*hdfR*c_Ec_ with flunixin, all *P* < 0.0001.

To assess whether the HdfR-MaoP regulatory module is amenable to chemical perturbation, we monitored *maoP* promoter activity in wild-type *Y. pestis* in the presence of a library of FDA-approved compounds. This screen identified flunixin as a compound capable of reducing *maoP* promoter activity in a dose-dependent manner at 37 °C, with up to a twofold decrease under the conditions tested (Fig. 5a). At the highest concentration active at 37 °C, flunixin did not reduce *maoP* promoter activity at 21 °C (Fig. 5b). Consistent with this temperature-restricted transcriptional effect, flunixin exposure did not reproducibly impair biofilm formation under flea-like conditions (21 °C) (Fig. 5c), but increased susceptibility of *Y. pestis* to antimicrobial peptides under mammalian host-like conditions (37 °C) (Fig. 5d, e). This peptide-sensitizing effect required HdfR, as flunixin did not further increase Δ*hdfR* susceptibility, even at polymyxin concentrations where mutant survival remained above the lower detection plateau (Fig. 5d, e). In line with this genetic requirement, heterologous expression of *E. coli hdfR* restored flunixin-dependent peptide sensitization (Fig. 5d, e). Furthermore, at the biochemical level, microscale thermophoresis using purified HdfR showed direct binding of flunixin to HdfR, with an apparent Kd of approximately 1 µM (Fig. 5f). Together, these results indicate that flunixin provides a chemical entry point to pharmacologically perturb the HdfR-MaoP regulatory module through a conserved HdfR-dependent mechanism.

## Discussion

**Fig. 6:**
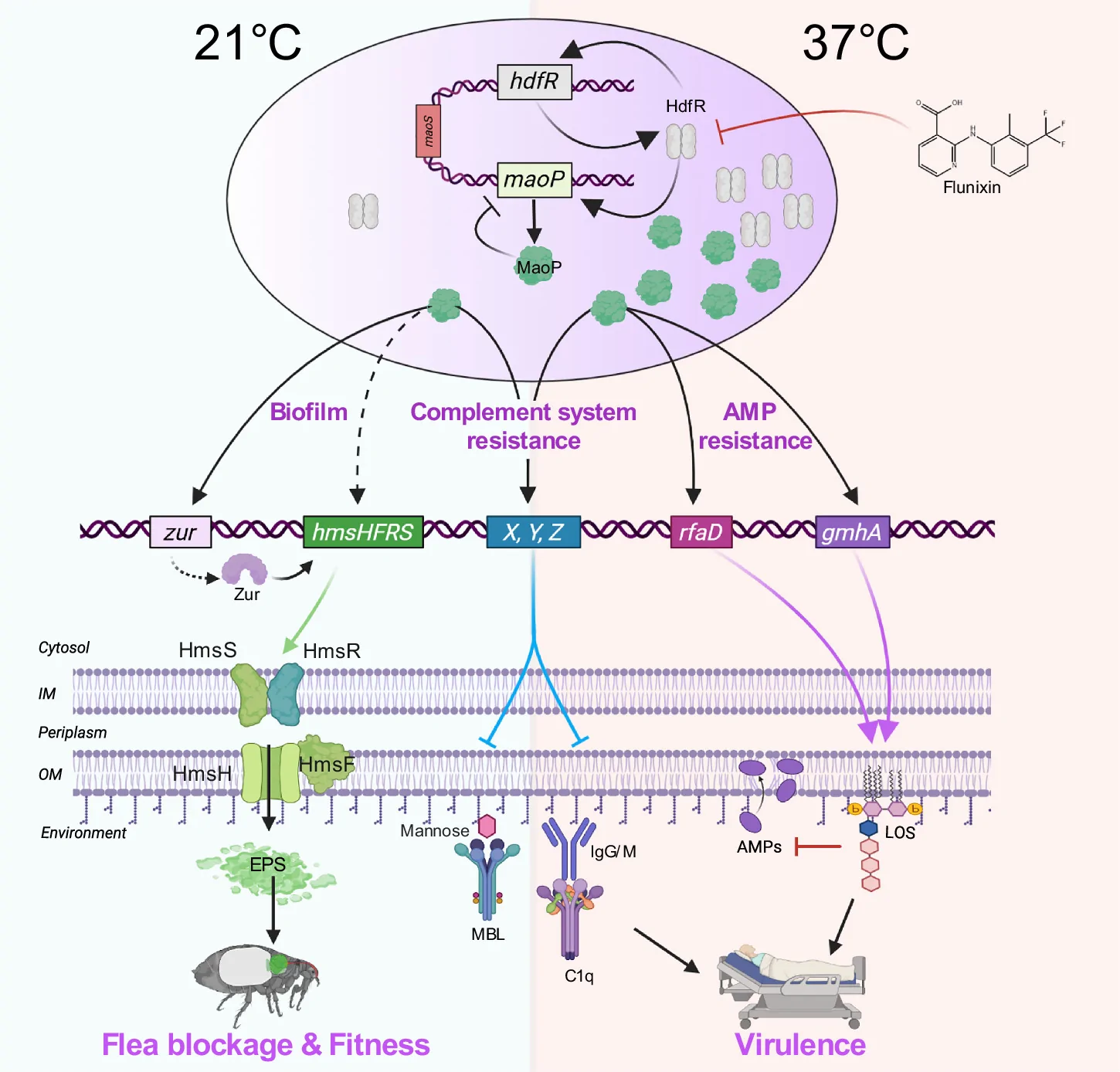
Model summarizing the HdfR-MaoP regulatory cascade and its role in host and vector adaptation. HdfR activates MaoP, forming a hierarchical regulatory module that controls biofilm-associated genes through both Zur-dependent and Zur-independent pathways and coordinates additional envelope adaptation programs. These coordinated molecular outputs support flea blockage and promote resistance to antimicrobial peptides and complement, thereby facilitating bacterial adaptation across vector and mammalian host environments. Temperature-dependent regulation highlights distinct roles of the HdfR-MaoP module under flea-like (21 °C) and mammalian (37 °C) conditions. Solid arrows indicate positive regulation, whereas blunt-ended lines indicate inhibitory effects. IM Inner membrane, OM outer membrane. Created in BioRender. Alexandre, B. (2026) https://BioRender.com/nitckrk.

Vector-borne pathogens face sharply contrasting selective pressures in arthropod vectors and mammalian hosts, requiring coordinated physiological reprogramming across abrupt environmental transitions^1,5^. How such opposing requirements are integrated at the regulatory level has remained incompletely understood. Here, using *Y. pestis* as a model of flea-borne transmission and mammalian infection, we identify the HdfR-MaoP module as a compact, temperature-responsive regulatory module that coordinates vector- and host-associated programs across successive stages of the bacterial life cycle (Fig. 6).

At the core of this regulatory architecture lies MaoP, whose expression is controlled by the transcriptional regulator HdfR. In this hierarchy, HdfR links temperature-dependent host-associated cues to *maoP* expression, whereas MaoP acts downstream as a central effector, and its effects depend on both its expression level and the environmental condition. Through this arrangement, the HdfR-MaoP module couples biofilm formation required for flea-borne transmission with envelope-associated adaptations that enhance resistance to innate immune defenses in the mammalian host. These coordinated outputs support a role for HdfR-MaoP as a regulatory hub for integrating the opposing selective pressures inherent to vector-borne life cycles.

Our mechanistic analyses clarify MaoP’s molecular function within this framework. MaoP is a small, highly abundant protein with a basic central region and the capacity to autoregulate its own expression. It binds DNA without detectable sequence specificity and alters DNA conformation in vitro, as shown by MST and AFM. By contrast, Hi-C contact maps and contact-probability decay analyses did not reveal major changes in chromosome folding in vivo. This difference likely reflects the different scales probed by the two approaches, with AFM detecting local single-molecule DNA conformational changes, whereas Hi–C reports population-averaged chromosomal contacts at kilobase resolution, as well as potentially dynamic or heterogeneous MaoP-DNA interactions, as previously reported for H-NS^33^. Thus, transient, heterogeneous, or sub-kilobase MaoP-DNA interactions may not produce detectable changes in global Hi-C profiles. This scale-dependent behavior is consistent with MaoP acting as a local architectural DNA-binding factor rather than as a global nucleoid organizer, a role classically associated with abundant NAPs such as HU or IHF^34,35^. Together, these biochemical and functional properties place MaoP within the NAP functional spectrum. However, rather than acting as an autonomous global nucleoid organizer, MaoP appears to function as a quantitatively tuned architectural effector embedded in an HdfR-controlled, temperature-responsive regulatory module. How MaoP functionally interfaces with established nucleoid-associated proteins and DNA-binding regulators remains to be defined. These factors may act together with MaoP to modulate virulence-associated gene expression under specific conditions.

The difference between MaoP’s broad DNA-binding capacity in vitro and its promoter occupancy in vivo helps reconcile its sequence-agnostic DNA binding in vitro with its discrete promoter occupancy in vivo. Controlled *maoP* expression in the Δ*hdfR* background restores all tested phenotypes, indicating that HdfR is not required as a promoter-specific cofactor for MaoP activity. Instead, HdfR appears to coordinate the module by tuning MaoP abundance, thereby linking MaoP’s broad DNA-binding capacity to vector- and host-aligned molecular programs. Consistent with this model, MaoP binds DNA in vitro without detectable sequence specificity, its ChIP-seq peaks lack a clear consensus motif, and bacterial two-hybrid assays detected no interaction with the RNA polymerase subunits or sigma factors tested. These findings argue against strict sequence-specific recognition or protein-mediated recruitment as the main determinants of MaoP occupancy. Rather, MaoP occupancy at discrete promoters likely emerges from broad DNA binding constrained by local chromosomal accessibility, topology, and physiological state, as described for NAPs such as H-NS^34,36^. In this view, MaoP illustrates how architectural DNA-binding proteins can be integrated into environmentally responsive regulatory circuits to coordinate adaptive outputs across changing host-associated niches. In this context, HdfR-mediated tuning of MaoP abundance may ensure that MaoP-dependent phenotypes are expressed under appropriate temperature conditions. Autoregulatory loops at both the HdfR and MaoP levels may further reinforce this robustness.

Beyond immune evasion per se, the HdfR-MaoP module coordinates anticipatory adaptations across host transitions. Through this regulatory axis, envelope properties are preconfigured under flea-like conditions and selectively reinforced upon entry into the mammalian host, supporting resistance to distinct arms of innate immunity. By limiting exposure of immunogenic surface determinants while strengthening membrane robustness under host-associated conditions, HdfR-MaoP enables *Y. pestis* to withstand both complement-mediated attack and antimicrobial peptide stress. This temperature-gated coordination may allow the bacterium to anticipate, rather than merely react to, host-associated immune pressures during transmission and early infection.

The HdfR-MaoP module also illustrates how conserved regulators can incorporate horizontally acquired virulence genes. In *S. enterica*, HdfR directly regulates the horizontally acquired *std* fimbrial operon^37^, showing that conserved regulators can be wired to species-specific virulence programs. In *Y. pestis*, HdfR-MaoP-dependent regulation similarly extends beyond chromosomal loci to selected plasmid-borne virulence-associated genes, including the pMT1-encoded *caf* locus. Thus, horizontally acquired functions can be incorporated into host-responsive regulatory architectures governed by conserved modules.

The widespread conservation of the *hdfR*-*maoP* locus across Enterobacterales indicates that this regulatory architecture predates the specialized flea-mammal life cycle of *Y. pestis*. In diverse bacterial species, components of this module have been linked to distinct physiological outputs, including virulence, biofilm-associated traits, motility and mutagenic repair of DNA breaks^38–43^. Consistent with this functional diversification, HdfR has been shown to bind directly to promoters controlling adaptive traits in other *Enterobacterales*, including the flagellar master regulator *flhDC* in *E. coli* and the *std* fimbrial operon in *S. enterica*^37,44^. Our data connect these observations by showing that the organization and regulatory hierarchy of HdfR-MaoP have been retained despite substantial sequence divergence. The functional conservation of this scaffold is supported by prior evidence that MaoP expression is HdfR-dependent in *E. coli*^45^, and by our cross-species complementation experiments showing that HdfR and MaoP from *E. coli* restore all tested phenotypes in the corresponding *Y. pestis* mutants. Conversely, HdfR and MaoP from *Y. pestis* restore biofilm formation in the corresponding *E. coli* mutants, despite the absence of the HdfR-MaoP-regulated *Y. pestis hmsHFRS* biofilm genes in *E. coli*. Together, these findings suggest that HdfR-MaoP is an ancient regulatory module whose outputs have diversified across ecological contexts, with downstream outputs shaped by species-specific physiology and niche-associated pressures. In *Y. pestis*, this scaffold appears to have been integrated into regulatory programs that coordinate flea-borne transmission and mammalian host adaptation.

In conclusion, the HdfR-MaoP module functions as a compact regulatory architecture that resolves how opposing host-specific programs are integrated during vector-borne life cycles characterized by abrupt transitions between ectothermic and mammalian environments. Through this mechanism, *Y. pestis* can efficiently navigate transitions between its arthropod vector and mammalian host. By coupling biofilm-mediated transmission with anticipatory envelope-associated programs that limit complement recognition, while reinforcing resistance to antimicrobial peptides, this circuitry provides a mechanistic link between transmission-associated biofilm formation and host-associated envelope adaptation, allowing *Y. pestis* to meet the conflicting demands imposed by alternating vector and mammalian environments. The widespread conservation of this architecture across Gammaproteobacteria highlights an ancient and versatile strategy for physiological plasticity and positions HdfR-MaoP as a conserved regulatory hub coordinating adaptation across distinct ecological contexts. Consistent with this view, the identification of flunixin as a chemical probe provides a means to experimentally manipulate this conserved regulatory architecture, offering tractable entry points to modulate host- and environment-associated physiological states in Gram-negative bacteria.

## Methods

### Bacterial strains, mutant construction, complementation, and growth conditions

All bacterial strains and plasmids used in this study are listed in Supplementary Data 3. *Y. pestis* strains were grown in Brain Heart Infusion (BHI) or Lysogeny Broth (LB) at 21 °C or 37 °C for 15 h without shaking unless otherwise specified. When required, media were supplemented with MgCl_2_ (4 mM), CaCl_2_ (4 mM), and carbenicillin (100 µg/mL), kanamycin (50 µg/mL), trimethoprim (25 µg/mL) or streptomycin (50 µg/mL). Mutations were generated using the λ Red recombinase system as previously described^46^. Briefly, the target sequence was replaced with an antibiotic resistance cassette amplified from the vectors listed in Supplementary Data 3 using the primer pairs shown in Supplementary Data 4. All constructs were verified by PCR. Complementation strategies were adapted to the experimental purpose. For in vitro assays, the gene of interest under its native promoter was cloned using the CloneJET PCR Cloning Kit (ThermoFisher Scientific), and the primer sets are listed in Supplementary Data 4. Alternatively, the coding sequence without its promoter was cloned into the pBAD30 expression vector for arabinose-inducible expression. For flea infection experiments, the gene under the control of its native promoter was integrated at the chromosomal attTn*7* site. For this, PCR products were first cloned into the suicide vector pUC18R6KT-mini-Tn*7*T in *E. coli* DH5α *λ*-pir. Recombinant constructs were then co-electroporated with the helper plasmid pTNS2 into *Y. pestis*, enabling site-specific integration at the att Tn*7* site^47^. Correct integration was confirmed by PCR.

### Antimicrobial peptide and serum resistance assay

For both assays, 5 × 10^5^ bioluminescent *Y. pestis* grown in LB at 21 °C or 37 °C were suspended in 10 µL of PBS (serum assay) or LB (antimicrobial peptide assay) and added to white 96-well plates (Fisher Scientific) containing 90 µL of the appropriate test solution, for a final volume of 100 µL per well. Plates were immediately sealed with a transparent film (ThinSeal™ ClearLine) and incubated at 21 °C or 37 °C without shaking. Luminescence was recorded at different incubation times using a Centro XS³ LB 960 luminometer (Berthold) and the Microwin 2010 5.24 software or a Victor™ X3 multilabel plate reader (PerkinElmer) and the PerkinElmer 2030 Manager 4.0. For the antimicrobial peptide assay, the test solution consisted of LB supplemented with increasing concentrations of polymyxin B (0–84 µg/mL; Sigma-Aldrich). For the serum resistance assay, the test solution consisted of either normal human serum, heat-inactivated serum (56 °C, 30 min)^48^; serum was purchased from the Établissement Français du Sang (EFS).

### Biofilm quantification and Congo red binding assays

Biofilm formation was quantified by crystal violet staining. A total of 2 × 10^6^ *Y. pestis* from overnight cultures grown at 21 °C with shaking were inoculated into 24-well flat-bottom plates containing 1 mL of LB supplemented with 4 mM MgCl_2_ and 4 mM CaCl_2_ per well. After 24 h incubation at 21 °C with shaking, the medium was removed, and the wells were washed three times with water. Adherent biofilms were stained with 0.01% crystal violet for 15 min at room temperature, washed three times, and bound dye was solubilized with 1.5 mL ethanol-acetone (80:20). Aliquots were transferred to transparent 96-well round-bottom plates, and absorbance was measured at 540 nm using a Biotek ELx800 spectrophotometer. Exopolysaccharide production was quantified by Congo red binding as previously described^49^. Bacteria grown at 21 °C with shaking in LB supplemented with 4 mM MgCl_2_ and 4 mM CaCl_2_ were centrifuged and resuspended in the same medium containing 0.2% galactose and 3% Congo red to reach 10^9^ CFU/mL. After 2.5 h of incubation at 21 °C without shaking, cultures were centrifuged, and the optical density of the supernatant was measured at 490 nm to quantify unbound dye.

### Flea infection

*The Xenopsylla cheopis* fleas used in this study were originally obtained from the Rocky Mountain Laboratories, National Institutes of Health (RML/NIH, USA), and have been maintained in our laboratory. Flea infections were performed as previously described^8^. Starved *X. cheopis* were randomly selected from the same laboratory colony and allowed to feed for 1 h on heparinized male OF-1 mouse blood (Charles River Laboratories) containing 5 × 10^8^ CFU/mL of *Y. pestis* grown overnight at 37 °C in BHI, using an artificial feeding system. Immediately after feeding, fully engorged fleas were randomly selected to generate cohorts of 100 fleas, either composed of equal numbers of males and females or of females only, and were selected and maintained in the dark at 21 °C and 75% relative humidity in a climatic chamber (Eratis). Fleas were fed twice weekly for 1 h on 2–5 day-old NMRI mouse neonates for up to 4 weeks. Neonates were obtained from an in-house breeding colony maintained under standard animal facility conditions: 12-h light/12-h dark cycle, 22 ± 2°C ambient temperature and 55 ± 10% relative humidity. Mouse sex was not determined because it could not be reliably assessed at this developmental stage. Mixed-sex cohorts were monitored twice weekly immediately after feeding to determine the blockage rate; blockage being defined as the presence of fresh red blood in the foregut but not in the midgut. Cohorts composed of females only were used to quantify the time course of gut colonization. At selected time points post-infection, fleas were surface-sterilized by sequential immersion in 1 mL of 3% hydrogen peroxide and 1 mL of absolute ethanol for 2 min each, rinsed in 10 mL of sterile PBS, and air-dried. Each flea was transferred into a Lysing Matrix H tube (MP Biomedicals) containing 1 mL of PBS and homogenized using a FastPrep-24 instrument (30 s, speed 6.0 m/s). Homogenates were mixed with LB agar supplemented with 1 µg/mL Irgasan and 10 µg/mL hemin and poured onto a base layer of the same medium. After drying, a second agar layer was added. Plates were incubated for 48 h at 28 °C before CFU enumeration. For co-infection experiments, fleas were infected with an equal mixture of two *Y. pestis* strains. Individual fleas were processed as described above, and homogenates were plated on non-selective supplemented LB agar to determine total bacterial loads and on selective supplemented LB agar to enumerate the antibiotic-resistant strain. The abundance of the competing strain was inferred by subtracting selective CFU counts from total CFU counts, and bacterial competition was quantified as the relative proportion of each strain within the total bacterial population recovered from each individual flea. All animal procedures were approved by the local Animal Care and Use Committee (Comité d’éthique en expérimentation animale n°75; reference: 2015102609372221 and 2022020316019750) and notified to the relevant French authorities, in accordance with current national legislation (Government Decree No. 2017-1411; Articles R.214-87 to R.214-126).

### Proteomic analysis

Wild-type and Δ*hdfR Y. pestis* strains grown in LB at 21 °C or 37 °C were harvested by centrifugation at 4 °C, washed twice with cold PBS, resuspended in PBS supplemented with an EDTA-free protease inhibitor cocktail (Roche) and then lysed by sonication using a Branson Digital Sonifier (2 cycles, 45 s on / 5 s off, 70% power, 4 °C). Lysates were clarified by centrifugation at 10,000 g for 20 min at 4 °C, and the supernatants were ultracentrifuged at 100,000 g for 40 min at 4 °C (Beckman Colter Optima XE-90). Protein concentrations were determined using the Bradford assay (Bio-Rad). Samples were dried in a SpeedVac™ concentrator (Thermo Scientific) and processed at the PISSARO proteomics platform (Université de Rouen Normandie, France) as already described^50^. Peptides were analyzed by nLC-MS/MS (RSLC Ultimate 3000 system coupled to an Eclipse mass spectrometer (Thermo Scientific)). MS/MS spectra were exported for peptide identification against the *Y. pestis* KIM10+ proteome (4079 proteins, Uniprot UP000002490) and containing 4079 sequences) using Proteome Discoverer 1.4 and Mascot 2.6.0 (Matrix Science). Database searches were performed with the following parameters: 1 missed trypsin cleavage site allowed; variable modifications: carbamidomethylation of cysteine and oxidation of methionine. Mass tolerances for precursor and fragment ions were set at 5 ppm and 0.02 Da respectively. The FDR was set at 1%. Quantification was performed using Progenesis 4.1 LC-MS/MS software (Waters) under a label-free quantification workflow. Peptides and proteins with *P*-value and q-value < 0.05 and statistical power > 0.8 were retained. Only proteins identified with ≥2 peptides and showing a fold change >2 between conditions were considered significant. Five biological replicates per strain and condition were analyzed.

### Bioinformatic analyses

Functional annotation and pathway classification of differentially regulated proteins were performed using the KEGG, NCBI, and UniProt databases.

### Metabolomic study

Metabolite profiling of *Y. pestis* strains was performed as described previously^51^. Briefly, bacteria grown in LB at 21 °C or 37 °C were harvested by centrifugation at 4 °C, and pellets were snap-frozen in liquid nitrogen. Metabolites were extracted at −20 °C using methanol/acetonitrile/water (50:30:20). Extracts were vortexed for 5 min at 4 °C, centrifuged at 16,000 *g* for 15 min at 4 °C, and supernatants were analyzed by LC-MS using a SeQuant ZIC-pHILIC column (Millipore). Chromatographic separation was performed using a linear gradient from 80% organic to 80% aqueous over 15 min at 200 µL/min, with the column maintained at 48 °C. The aqueous mobile phase contained 20 mM ammonium carbonate with 0.1% ammonium hydroxide, and the organic phase was acetonitrile. Mass spectrometry was performed on a Q-Exactive Plus instrument (Thermo Fisher Scientific) operating in electrospray ionization with polarity switching, at 35,000 resolution at m/z 200 over an m/z range of 75-1000. Lock masses ensured mass accuracy <5 ppm. Peak areas were extracted using TraceFinder (Thermo Fisher Scientific) based on the exact mass of the singly charged ion and known retention times on the HPLC column. Statistical analyses were performed using MetaboAnalyst 4.0^52^. Hierarchical clustering was performed using a Pearson correlation-based distance metric and Ward linkage to group metabolites according to abundance patterns.

### Promoter activity assays

Promoter activity was quantified using luciferase- or GFP-based transcriptional reporters. For the luciferase assay, a *Y. pestis* strain harboring at the att Tn*7* site a promoterless *luxCDABE* operon downstream of a kanamycin-resistance cassette preceded by a single I-*Sce*I restriction site was used^53^. The promoters of interest were amplified by PCR and inserted in place of the kanamycin cassette and the I-*SceI* restriction site using the *λ* Red recombinase system with I-*Sce*I counter-selection^8^, employing the plasmids and primer pairs listed in Supplementary Data 3 and 4. All constructs were verified by PCR and sequencing. Cultures grown in LB at 21 °C or 37 °C were transferred into white 96-well plates (Fisher), and luminescence was recorded for 1s using a Centro XS³ LB 960 luminometer (Berthold). Luminescence values were normalized to OD_600_. For the GFP assay, the promoters amplified by PCR were cloned into the pPromX-GFP plasmid. *Y. pestis* harboring the recombinant plasmids were grown in LB at 21 °C or 37 °C, washed three times in PBS and transferred into black 96-well plates (Fisher) using excitation/emission settings of 488/535 nm. GFP fluorescence was immediately recorded for 1s using a Victor™ X3 multilabel plate reader (PerkinElmer) and normalized to OD_600_ measured after the final wash.

### Western-blotting

*Y. pestis* was directly resuspended in Laemmli sample buffer (Bio-Rad) containing β-mercaptoethanol after being grown in LB or after three washes with PBS when grown in human serum. Samples were boiled at 95 °C for 10 min and whole-cell lysates were separated by SDS-PAGE. Proteins were transferred onto nitrocellulose membranes (Amersham), which were then blocked in PBS containing 0.5% Tween-20 (PBS-T) and 5% skim milk. Membranes were incubated with the appropriate primary antibody (Supplementary Data 5), washed four times in PBS-0.5% Tween-20 and incubated for 45 min at room temperature with the corresponding HRP-conjugated secondary antibody (Supplementary Data 5). After four additional washes, signals were detected using SuperSignal West Pico (Thermo Fisher). Images were acquired using a LAS-3000 imager (Fujifilm) or an ImageQuant 800 system (Cytiva) and analyzed with the Multigauge 2.1.1 and ImageQuant TL 10.2, respectively (Supplementary Data 5). Band intensities were normalized to the RNA polymerase alpha subunit, to Ponceau or Coomassie blue-stained gels. Specific antibody conditions are provided in Supplementary Data 5.

### DOC-PAGE assay

DOC-PAGE was performed as previously described^54^. A total of 7.5 × 10^8^ *Y. pestis* grown at 21 °C or 37 °C were harvested by centrifugation, washed once in PBS, and resuspended in DOC lysis buffer (2% sodium deoxycholate, 4% 2-mercaptoethanol, 10% glycerol, and 0.002% bromophenol blue in 1 M Tris-HCl buffer, pH 6.8). Suspensions were heated for 10 min at 100 °C, treated with Proteinase K (80 μg/mL) for 3 h at 55 °C, and the reaction was stopped by an additional 10 min incubation at 100 °C. Equivalent amounts of material equivalent to 2 × 10^7^ bacteria per lane were loaded onto DOC-PAGE gels, and LOS was visualized by silver staining.

### Outer membrane permeability assay

Outer membrane permeability was assessed using the 1-N-phenylnaphthylamine (NPN) uptake assay as previously described^55^. *Y. pestis* grown in LB at 21 °C or 37 °C was harvested, washed three times in PBS, and adjusted to 10^9^ CFU/mL. NPN (10 µM final) was added to the suspension and fluorescence was recorded after 30 min incubation in the dark at 21 °C or 37 °C (excitation 350 nm, emission 420 nm) using a Victor™ X3 multilabel plate reader (PerkinElmer). Background fluorescence (PBS + NPN) was subtracted from all readings, and values were normalized to the bioluminescence of the same suspensions, used as a proxy for bacterial load.

### Detection of deposited serum components

Deposition of complement factors and serum proteins (C3b, C4, C4BP, factor H, factor B, vitronectin, C1q and MBL) on the bacterial surface was assessed by Western blot as previously described^56^, using the antibodies listed in Supplementary Data 5. *Y. pestis* was incubated with normal or heat-inactivated human serum at a final concentration of 90% (50% for assays involving factor H) using ~7.5 × 10^6^ bacteria per reaction (~10^9^ for factor H because higher bacterial input was required for detection). At the indicated time points, samples were chilled on ice and centrifuged at 4 °C, washed three times in cold PBS, resuspended in Laemmli sample buffer containing β-mercaptoethanol, and analyzed by Western blot as described above.

### RNA extraction and RT-qPCR

Total RNA was extracted by resuspending *Y. pestis* pellets in TRI Reagent (Thermo Fisher Scientific), followed by the addition of chloroform (20% final; vortexed and incubated at room temperature for 10 min). After phase separation, the aqueous phase was mixed 1:1 with ethanol, and RNA was pelleted by centrifugation. Pellets were washed with 75% ethanol, centrifuged again, air-dried and resuspended in RNase-free water. Residual genomic DNA was removed by DNase I treatment (Merck). RNA concentration and purity were measured spectrophotometrically, and RNA integrity was verified by agarose gel electrophoresis. cDNA synthesis was performed using SuperScript III Reverse Transcriptase (Thermo Fisher Scientific) and random hexamers. Quantitative PCR was carried out using Takyon Rox SYBR MasterMix dTT Blue (Eurogentec) on a Stratagene Mx3005P instrument (Agilent Technologies), with fluorescence acquisition and Ct determination performed using MxPro 4.10 software. Relative transcript levels were calculated using the ΔΔCt method with *proC* as the internal reference^57^. Primer sequences are listed in Supplementary Data 4.

### High-throughput chromosome conformation capture (Hi-C)

*Y. pestis* (Δ*pla*) cells crosslinking and Hi-C library preparation were performed as described in^58^. Hi-C sequencing data were processed starting with adapter trimming using Cutadapt 3.2. Paired-end reads were then aligned to the *Y. pestis* KIM10+ genome (accession number: PRJNA288) using Bowtie2 (v2.4.2). Hi-C data were further analyzed using the OCHA pipeline in R (https://github.com/js2264/OHCA), as described in^59^. For visualization, the contact maps were displayed at 1 kb bin resolution. To qualitatively compare normalized contact maps, we performed a ratio of matrices. This ratio is computed for each bin of the map by dividing the amount of contacts it presents in Δ*maoP* by the amount of contacts in the WT, and applying a log2 transformation to the resulting matrix. The log2 of the ratio is plotted with a Gaussian filter (H = 1). The color code reflects a decrease or an increase in contacts in one condition compared to the other (blue or red signal, respectively). No change is represented by a white signal. To quantitatively compare chromosome organization between strains, contact probability was calculated as a function of genomic distance from normalized Hi–C contact matrices. Contact-probability decay curves were compared between WT Δ*pla* and Δ*maoP* Δ*pla* strains to assess potential changes in short- and long-range chromosomal contacts.

### ChIP-seq

ChIP-seq was performed as previously described with minor modifications^60^, using three independent biological replicate cultures per strain. *Y. pestis* lacking pCD1 and *pla*, while expressing a C-terminal FLAG-tagged MaoP protein (MaoP-FLAG), together with the corresponding untagged parental strains (negative controls) were grown in LB at 37 °C; MaoP-FLAG were being functional (Supplementary Fig. 4a, b). Cultures were crosslinked with 1% formaldehyde for 30 min at 37 °C under gentle agitation, quenched with glycine, washed with TBS, pelleted and resuspended in IP buffer. Chromatin was sheared to 100–300 bp fragments using a Bioruptor sonicator (40 cycles, 30 s on / 30 s off, high power, 4 °C). Complete compositions of buffers are provided in Supplementary Data 6. Lysates were incubated overnight at 4 °C with anti-FLAG magnetic beads (GenScript). Beads were washed, and bound DNA was eluted with alkaline elution buffer prior to reverse-crosslinking Protein-DNA complexes overnight at 65 °C. DNA was purified by phenol:chloroform:isoamyl alcohol (25:24:1, pH 4.0) extraction, precipitated in precipitation buffer (30:1, v/v) overnight at −20 °C, washed with ethanol 70% and resuspended in TE buffer. 50 ng of fragmented double-stranded DNA was used for library preparation using the MicroPlex Library Preparation kit v3 (Diagenode). Dual-indexed libraries were sequenced on an Illumina NextSeq 500 sequencer. Reads were processed using the Galaxy server^61^, mapped to the *Y. pestis* KIM genome with Bowtie2 2.5.5 + galaxy0^62^, and peaks were called with MACS2 2.2.9.1 + galaxy0^63^ using default settings.

### Purification of recombinant proteins

The *maoP* and *zur* genes from *Y. pestis* were amplified by PCR and cloned into the pET24d or pET28a vectors to generate 6His-tag fusion constructs, using the primers and plasmids listed in Supplementary Data 3 and 4. Complete compositions of TNTI, TNI, and TN buffers, as well as all reagent formulations used for expression and purification, are provided in Supplementary Data 6. The recombinant plasmids were transformed into *E. coli* C41(DE3). Cultures were grown in LB at 37 °C to an OD_600_ of ~0.4, induced with 1 mM IPTG for 3 h at 37 °C, harvested, resuspended in PBS and lysed using a French press (Constant Systems). Clarified lysates were loaded onto HisPur™ affinity columns (Thermo Fisher) packed with complete His-Tag Purification Resin (Roche). Proteins were washed with TNTI and TNI buffers, and eluted with TNI containing 500 mM imidazole. Proteins were concentrated and exchanged into TN or TN AFM buffer using Amicon Ultra-15 devices (Merck). Purity was verified by SDS-PAGE.

### Microscale thermophoresis (MST)

MST measurements were performed on a Monolith NT.115 Pico instrument (NanoTemper Technologies). Purified MaoP and Zur proteins were exchanged into TNG buffer (Supplementary Data 6) and quantified by BCA assay. Proteins were diluted to 200 nM in PBS-T (NanoTemper) and labeled with 100 nM RED-tris-NTA dye for 30 min at room temperature in the dark, then diluted to a final concentration of 10 nM. DNA ligands consisted of purified PCR products (amplified from sequences listed in Supplementary Data 4). PCR products were purified using a PCR cleanup kit (Macherey Nagel), quantified by spectrophotometry, and serially diluted in a 1:1 ratio across 16 concentrations to generate the ligand titration series. Equal volumes of labeled protein and diluted DNA were mixed and incubated for 5 min at room temperature before loading into standard capillaries. Thermophoresis was recorded using medium MST power and 20% LED. Data were acquired with MO. Control 1.6.1 and analyzed with MO.Affinity Analysis 2.3 (NanoTemper) (Supplementary Data 6).

### RNA immunoprecipitation (RIP)

RIP was performed on *Y. pestis* lacking pPla and expressing the *E. coli* MaoP-3×FLAG from the pAK004 plasmid. Cultures were grown overnight in LB at 37 °C supplemented with 10 mM arabinose, harvested, washed once in cold PBS and lysed by bead beating (Biospec) in ice-cold lysis buffer (Tris-HCl pH 7.4, 150 mM NaCl, 1 mM EDTA, 0.5% Triton X-100). Lysates were clarified by centrifugation and incubated with anti-FLAG magnetic beads (GenScript) for 2 h at 4 °C with gentle rotation. Beads were washed four times with TBS, and MaoP-RNA complexes were eluted with RIP elution buffer using a thermomixer (70 °C, 800 rpm, 5 min). RNA was extracted with phenol:chloroform:isoamyl alcohol (25:24:1, pH 4.0), precipitated overnight at -20°C with precipitation buffer, washed with 70% ethanol, air-dried, then resuspended in nuclease-free water. RNA samples were treated with DNase I (Merck) prior to being assessed for integrity by agarose gel electrophoresis and quantity with a Qubit RNA High Sensitivity kit (Thermo Fisher). Details on buffer compositions, washing conditions, and centrifugation parameters are provided in Supplementary Data 6.

### c-di-GMP quantification

c-di-GMP was extracted and quantified by HPLC as described previously^64^, with minor modifications. Briefly, bacterial cultures were fixed with 0.18% formaldehyde, pelleted, washed, and resuspended in distilled water. An aliquot was used for protein normalization by BCA assay, while the remaining sample was heated at 99 °C and extracted sequentially with 95% and 70% ethanol. Dried extracts were resuspended in nanopure water and analyzed on a Dionex Chromeleon 7 HPLC system equipped with a reverse-phase C18 column. c-di-GMP was eluted using ammonium acetate-based water/methanol solvents, detected at 253 nm, quantified using a commercial c-di-GMP standard curve, and normalized to total protein content. Details on buffer compositions are provided in Supplementary Data 6.

### Determination of MaoP copy number per bacteria

MaoP copy number per bacterium was estimated by quantitative immunoblotting. Clarified lysates were incubated with anti-FLAG magnetic beads (GenScript) for 2 h at 4 °C with gentle rotation. Beads were washed four times with Wash buffer, and MaoP-FLAG was eluted using the Alkaline elution buffer. After neutralization as recommended by the manufacturer, the purified proteins were quantified by BCA assay. Purified MaoP-FLAG was used as a calibration standard and loaded alongside whole-cell extracts prepared from 2.5 × 10^8^ *Y. pestis* grown at 21 °C or 37 °C. Samples were resolved by electrophoresis, transferred, and probed with an anti-FLAG antibody. Band intensities were quantified and compared with the purified MaoP-FLAG standard curve to determine the amount of MaoP-FLAG in each extract. Molecule numbers were derived using the predicted molecular weight of MaoP-FLAG (14,065 kDa) and Avogadro’s constant, then normalized to bacterial cell counts. Details on magnetic beads and Antibody Supplementary Data 5, as well as buffer compositions, are provided in Supplementary Data 6.

### Bacterial two-hybrid (BACTH) assay

Protein-protein interactions were assessed using the adenylate cyclase-based BACTH system (Euromedex) following the manufacturer’s instructions. Candidate genes were cloned into the pKT25 vector, and *maoP* was cloned into the pUT18 vector using the primers listed in Supplementary Data 4. The resulting plasmid pairs were co-transformed into *E. coli* DHM01 (Δ*cyaA*). Transformants were plated on MacConkey agar supplemented with maltose 1%, ampicillin and kanamycin, and incubated at 30 °C until colony color discrimination was evident. The pKT25-Zip and pUT18C-Zip plasmid pair was used as the positive control.

### Atomic force microscopy

DNA and protein samples were prepared and imaged in liquid according to a previously published protocol^65^. DNA was obtained after PCR using primer sets in Supplementary Data 4, and subsequently was extracted with phenol:chloroform:isoamyl alcohol (25:24:1, pH 4.0), precipitated overnight at −20 °C, washed with ethanol 70% and resuspended in nuclease-free water. Purified MaoP in TN AFM buffer was obtained as described above. Samples were immobilized onto freshly cleaved mica (10 mm, Ted Pella, US) by incubating 4 µl of 10 ng/µl solution in a 70 µl drop of divalent cations buffer during 10 min for DNA or 30 min for DNA mixed with purified MaoP. Samples were then washed four times in the same buffer to remove unbound material and rinsed in Ni buffer to stabilize DNA-protein complexes during AFM scanning. Imaging was conducted on a FastScan Bio microscope (Bruker, US) operating NanoScope 9.4 software in Peak Force Tapping mode, using FastScan-D probes (Bruker, US). Engage and Peak Force imaging setpoints were minimized to [40–100] pN. Peak Force parameters were as follows: 2 kHz PF frequency, [10–20] nm PF amplitude, [150–1000] μm scan size, [256–512] pixels, [1–3] Hz scan rate, [5–10] nm lift height, [2–5] gain. Sync distances were adjusted at engagement and kept constant throughout imaging. Images were plane-fitted, cropped (if needed), and exported using NanoScope Analysis 1.9. Complete compositions of divalent cations buffer, Ni Buffer and TN AFM buffers, as well as all reagent formulations used, are provided in Supplementary Data 6. For DNA Bend angles analysis, only DNA molecules that were completely visible on the image and could be unambiguously traced were taken for analysis. DNA bend angles were measured using ImageJ 1.52, a software. The angles were defined by manually determining the path of the DNA segments. By convention, the DNA bend angle is defined as the deviation from straight.

### Identification of compounds inhibiting the HdfR-MaoP module

Inhibitors of the HdfR-MaoP module were identified using *Y. pestis* carrying a chromosomal *maoP*-*luxCDABE* reporter integrated at the attTn*7* site. Approximately 10^6^ bacteria were dispensed into 384-well plates containing 50 µL of LB medium with or without individual compounds from an FDA-approved compound library (Tocris and Prestwick). Plates were incubated at 37 °C, and luminescence (RLU) and optical density (OD_595_) were measured at 0, 4, and 8 h using a Victor™ X3 plate reader (PerkinElmer). Luminescence values were normalized to OD_595_ to control for bacterial growth. Compounds that reduced *maoP* promoter activity by at least twofold relative to DMSO controls, without affecting OD_595_, were selected for dose-response analyses. Selected compounds were tested at concentrations ranging from 6 nM to 50 µM, performed in triplicate. The lead compound was validated by assessing *Y. pestis* survival after exposure to polymyxin B (1.25 µg/mL) and the lead compound (25 µM), alone or in combination (in the presence of the DMSO vehicle control), as described in the antimicrobial peptide susceptibility assays section.

#### Statistical analysis

Statistical analyses were performed using GraphPad Prism 10 (GraphPad Software). For experiments comparing multiple groups to a single control, an ordinary one-way analysis of variance (ANOVA) followed by Dunnett’s multiple comparisons test was used. For antimicrobial peptide resistance assays, comparisons between strains across tested doses were analyzed using a two-way ANOVA followed by Tukey’s multiple comparisons test. Data are presented as mean ± standard deviation (SD). n values correspond to distinct biological samples or independent experimental units, not repeated measurements of the same sample. A *P* value < 0.05 was considered statistically significant. ns, not significant; *P < 0.05.

### Reporting summary

Further information on research design is available in the Nature Portfolio Reporting Summary linked to this article.

## Supplementary information

Supplementary Information

Description of Additional Supplementary Files

Supplementary Data 1

Supplementary Data 2

Supplementary Data 3

Supplementary Data 4

Supplementary Data 5

Supplementary Data 6

Reporting Summary

Transparent Peer Review file

## Source data

Source data

## Data Availability

The mass spectrometry proteomics data generated in this study have been deposited with the ProteomeXchange Consortium via the PRIDE^66^ partner repository under dataset identifier [PXD071830] (https://www.ebi.ac.uk/pride/archive/projects/PXD071830). The processed proteomics identification and quantification data generated in this study have been deposited in Zenodo under (https://doi.org/10.5281/zenodo.18209150). The metabolomics data generated in this study have been deposited in Zenodo under (https://doi.org/10.5281/zenodo.17786388). The ChIP-seq data generated in this study have been deposited in NCBI under BioProject accession [PRJNA1372475] (https://www.ncbi.nlm.nih.gov/bioproject/PRJNA1372475). The pPromX-GFP sequence generated in this study has been deposited in GenBank under accession number [PX762416](https://www.ncbi.nlm.nih.gov/nuccore/PX762416). The Hi-C data generated in this study have been deposited in the Sequence Read Archive (SRA) under BioSample accessions [SAMN54411906] (https://www.ncbi.nlm.nih.gov/biosample/SAMN54411906) and [SAMN54411907] (https://www.ncbi.nlm.nih.gov/biosample/SAMN54411907). Source data are provided with this paper. Oligonucleotide sequences and plasmid information are provided in Supplementary Data 3 and 4, respectively. Newly generated materials are available from the corresponding author upon request, subject to applicable institutional biosafety regulations, material transfer agreements and regulatory requirements. Source data are provided with this paper.
