# Supplementary material for: A dual-host regulatory hub integrating biofilm formation and innate immunity resistance
Source: Nat Commun. 2026 Sep 1;17:10385. doi: 10.1038/s41467-026-77307-4 (PMC13627719; doi:10.1038/s41467-026-77307-4)
Supplement: Supplementary file 1 [file 41467_2026_77307_MOESM10_ESM.pdf]

nature portfolio

Peer Review File

A dual-host regulatory hub integrating biofilm formation and innate immunity resistance

Corresponding Author: Dr Florent Sebbane

This file contains all reviewer reports in order by version, followed by all author rebuttals in order by version.

Attachments originally included by the reviewers as part of their assessment can be found at the end of this file.

Version 0:

Reviewer comments:

Reviewer #1

(Remarks to the Author)
Summary and significance
The authors describe a regulatory module in Yersinia pestis consisting of HdfR, a flea-induced transcriptional regulator, and MaoP, a nucleoid-associated protein. They propose that this HdfR–MaoP module coordinates adaptation across flea and mammalian host environments by promoting biofilm formation in the flea vector while altering outer membrane architecture during mammalian infection to support immune evasion. In this model, the circuit integrates environmental cues, particularly temperature, to drive context-specific phenotypic outputs. The authors further suggest that this regulatory module is conserved among enteric bacteria and demonstrate that it can be pharmacologically targeted through small-molecule inhibition of HdfR-dependent activation. Collectively, they argue that the HdfR–MaoP module represents an essential component of the vector-borne life cycle of Y. pestis and a broadly conserved adaptive strategy.

The idea that a conserved regulatory module links nucleoid-associated regulation with host-specific adaptation is conceptually compelling. If fully substantiated, the work would be of broad interest to the fields of bacterial pathogenesis, regulatory biology, host–pathogen interactions, and evolutionary microbiology. The proposed integration of environmental sensing with downstream regulatory outputs represents a potentially important conceptual advance.

However, the mechanistic depth and integrative framework currently fall short of supporting the broader claims made regarding life-cycle coordination and evolutionary conservation.

Major Comments:
Despite the strong conceptual framework, the mechanistic evidence presented does not yet fully support the breadth of the claims regarding coordinated life-cycle regulation and rapid host transition. While many experiments are technically sound, several conclusions extend beyond what is directly demonstrated.

A key concern is the regulatory logic of the module. Transcriptional control of maoP by HdfR is presented as the central regulatory node, yet MaoP appears expressed in both flea and mammalian conditions without a clear explanation for how its activity is differentially modulated. It remains unclear whether maoP is upregulated in the flea as predicted and how differences in transcript levels alone account for distinct phenotypic outputs. If MaoP is present across conditions, additional regulatory layers—such as altered DNA binding, protein–protein interactions, localization, or post-translational modulation—may be required to explain environment-specific effects. Similarly, the proposed role of the module in rapid flea-to-mammal transition is not directly tested, as most analyses rely on steady-state temperature comparisons rather than acute transition models.

The study uses an appropriate experimental toolkit, including transcriptional profiling, genetic perturbation, phenotypic assays, Hi-C analysis, and pharmacological inhibition. However, the work relies heavily on expression-level correlations, with limited direct mechanistic interrogation of MaoP's functional activity. Some interpretive claims—such as temperature-dependent mutant phenotypes, the exclusion of LOS pathway effects in complement sensitivity, the absence of significant Hi-C changes without quantitative metrics, and assertions of conservation and "core functional logic"—would benefit from clearer supporting data and definition. The pharmacological experiments also require stronger specificity controls and

broader phenotypic validation.

Overall, the manuscript is ambitious and generally well written, but several claims are framed more strongly than the supporting evidence warrants. Clearer articulation of regulatory hierarchy, direct testing of transition dynamics, stronger quantitative rigor, and deeper contextualization within the literature on nucleoid-associated proteins and bacterial adaptation would substantially strengthen the work. In its current form, the manuscript would benefit from substantial mechanistic clarification and additional validation before the central claims can be fully supported.

Minor/Line Comments:
Lines 83-85: Was maoP also identified as being upregulated in the flea? The proposed model would seemingly predict this to be true but is not seemingly specified here or elsewhere.

Line 100: The statement “Despite this broad impact…” implies that MaoP is the primary downstream factor, but this is not obvious from the presentation.

Line 100: Proteomics data is shown for 37°C but screen identified HdfR as being upregulated in the flea. So it was not clear how examination of the proteomics at the mammalian temperature was the most-relevant condition to examine.

Line 158: Extended Data Figure 6a is cited as supporting temperature-dependence of maoP mutant, but supporting data is shown for hdfR mutant and it is not clear is this faithfully applies also to the maoP mutant. Does hdfR mutation also reduce LOS production at 37°C?

Line 165: The conclusions presented in statement “By contrast…” are not obvious. It is not clear how LOS pathway perturbation was ruled out as an underlying cause of complement sensitivity.

Lines 186-187: Data is not shown for RIP and two-hybrid assays which are important findings given the systematic evaluation of MaoP function and its proposed role in chromosomal origin maintenance.

Line 186: It is stated that there are no significant changes to Hi-C map despite apparent signatures on the comparative interaction map. No metrics were provided to quantify changes, which made it hard to evaluate this claim.

Lines 188-189: Would be useful to be explicit about location of MaoS site. It was not immediately obvious that this was present in the intergenic region between hdfR and maoP until a thorough consultation of previous work.

Line 191: Not clear what is meant by conservation of MaoP function in this context. Does the author mean to imply that function of MaoP is independent of the MaoS site? My guess is that this is related to downstream arguments for the protein product being the primary driver of MaoP-dependent phenotypes.

Line 231: What is the baseline sensitivity in this assay? Is it possible that the lack of additive sensitivity in the hdfR mutant is due to a floor in the sensitivity? Additionally, data is only shown for one promoter. It is formally possible that the drug is not specific to a single promoter, which would alter the interpretation of these findings. It would have also been more compelling to see how this drug affects other relevant phenotypes shown such as biofilm formation.

Line 262: Not clear what characteristics of canonical nucleoid-associated proteins are not displayed by MaoP. These distinctions are presented as central to the novelty of this module, so these differences should be more explicitly highlighted.

Line 264: Transcriptional regulation of maoP by HdfR is presented as a key aspect of the HdfR-MaoP module, but it is unclear how this is specifically coordinating activity with respect to the module. It appears that MaoP is presented and expressed (albeit possibly at different levels) across temperatures, but is not clear how this level of regulation explains differences in apparent activities of this module in both conditions. It appears that this is an important aspect of the module that is missing from the data presented.

Line 284: Not clear what is implied by “core functional logic”.

Line 285: The HdfR-dependence of MaoP expression appears to be important foundational work for this study but is only cursorily mentioned here.

Line 289: What data supports role of this module in other bacteria?

Figure 2c-d: At what temperature are these experiments performed?

Figure 2f: Data points on the X-axis for Wt, hdfR mutant, and complement at 27 days post infection.

Figure 3: Panel d is awkwardly placed in vertical arrangement.

Line 581: Could not find how differential CFU counts were used in these assays.

Line 710: Reference 48 is now published.

Reviewer Expertise
My expertise is in bacterial regulatory networks and chromosomal organization in pathogenic bacteria. My comments therefore focus on mechanistic sufficiency, regulatory logic, and integration of transcriptional and structural data.

Reviewer #2

(Remarks to the Author)
This manuscript describes the identification and characterisation of a regulatory hub in Y. pestis involved in controlling the transmission from flea to mammal. The manuscript shows that the HdfR-MaoP hub is required by Y. pestis for normal resistance to mammalian innate immunity effectors such as AMPs and biofilm formation (in the flea gut). As such the authors propose that this minimal regulatory hub "provides a mechanistic framework for overcoming the conflicting demands imposed by alternating vector and mammalian environments".

Comments

line 184: MaoS sites are mentioned but the location of these sites is not indicated anywhere in the manuscript.
line 199: MaoP apparently binds DNA in a sequence-agnostic manner but, nonetheless, in discrete promoter regions. The authors argue that this specificity may be due to local chromosomal topology. Does HdfR (a bona fide transcriptional regulator) have a recognition sequence that perhaps imparts specificity to a HdfR-MaoP complex?? Are there HdfR-regulated genes that are dependent and independent of MaoP??
Figure 3b: the DOC-PAGE presented is not of great quality. Do all LOS bands have the same mobility?

Reviewer #3

(Remarks to the Author)
This work led by Baillez et. al. identifies the HdfR-MaoP module as a putative regulatory hub governing host transition in Yersinia pestis. The authors argue that HdfR-dependent activation of MaoP coordinates flea-associated biofilm formation with resistance to mammalian host immunity, and further propose that this regulatory logic is conserved across enteric bacteria. The study combines extensive multi-omics data, biochemical analyses, and infection models to investigate this pathway, and the broader evolutionary framework is conceptually appealing. However, several key mechanistic gaps remain unresolved, and addressing these issues would substantially strengthen the manuscript.
1. The major weakness of the work is the lack of mechanistic gaps and discrepancies between assays, and some areas requiring further clarification. My major concern is the function and mechanism of the nucleoid-associated protein-like factor MaoP, which sits in the central part in the proposed regulatory switch. The authors demonstrate via microscale thermophoresis that the MaoP binds DNA in a sequence-agnostic manner in Fig4b. However, ChIP-seq reveals MaoP occupancy at discrete promoter regions (such as its own locus, zur, and hmsHFRS). It is not sufficiently explained how a sequence-agnostic DNA-binding protein achieves this highly specific transcriptional targeting. The manuscript would benefit from a proposed model or additional experiments explaining how MaoP is recruited to specific promoters (e.g., through protein-protein interactions with other specific transcription factors, despite the negative bacterial two-hybrid results with RNA polymerase).
2. There is a notable discrepancy regarding MaoP's effect on DNA topology. In vitro atomic force microscopy (AFM) visualizes local changes in DNA conformation upon MaoP binding. Conversely, in vivo Hi-C reveals no major changes in global or local chromosome folding. While the authors speculate this may be due to highly dynamic or heterogeneous MaoP-DNA interactions, they should discuss whether the resolution of their Hi-C mapping (1kb bins) is sufficient to detect the fine, local topological bends observed via AFM, or if other in vivo assays could bridge this gap.
3. The proteomic data indicate that MaoP is a relatively abundant DNA-binding protein, yet the manuscript does not discuss this abundance relative to established Yersinia nucleoid-associated factors such as H-NS, YmoA/Hha, IscR. Such comparison would be valuable, particularly because it remains unclear whether MaoP performs overlapping, complementary, or distinct regulatory functions. At minimum, the authors should discuss whether these small DNA-binding proteins are deployed in different environmental niches or act cooperatively to fine-tune virulence-associated programs during the complex life cycle of Y. pestis.
4. The manuscript focuses primarily on chromosomal regulatory effects, but does not address whether MaoP-dependent DNA binding and topological changes could also extend to the virulence plasmids of Y. pestis. This is an important omission, given that the type III secretion system is encoded on pCD1 and that additional plasmids, including pMT1 and pPCP1/pPla, are central to pathogenicity and flea-mammal life circle. The authors should discuss whether MaoP might affect plasmid topology and thereby influence plasmid-encoded virulence functions.
5. The conclusion that MaoP regulates hmsHFRS through parallel Zur-dependent and Zur-independent pathways raises an important mechanistic question that is left unresolved. Because the Δzur mutant displays a less severe phenotype than ΔmaoP, while the double mutant shows the strongest defect, the authors infer the existence of an additional Zur-independent route connecting MaoP to biofilm regulation. However, no hypothesis is offered. In addition, the function of Zur itself deserves more careful consideration. I wonder if Zur is simply a zinc uptake regulator, the authors should discuss the environmental contexts or host niches in which Zur-dependent control would be expected to operate during the Y. pestis life cycle. It would also be useful to clarify whether Zur acts solely in zinc homeostasis here or may intersect more broadly with other metal-responsive regulatory networks, particularly those related to iron, which could be more physiologically relevant in infection settings.
6. The authors provide convincing evidence that MaoP limits complement activation by restricting the binding of mannose-binding lectin (MBL) and C1q. They conclude that the HdfR-MaoP module "masks surface ligands across temperatures to

restrict complement activation". However, the actual identity of these immunogenic surface determinants remains unknown. Is it possible to identify which specific outer membrane components or surface ligands are being masked to prevent classical and lectin pathway activation?
7. In Fig 3, the authors links AMP susceptibility at 37°C to impaired lipooligosaccharide (LOS) core maturation, specifically through reduced expression of gmhA and rfaD. However, ectopic expression of either gmhA or rfaD in the ΔmaoP mutant only partially restored polymyxin resistance. This partial rescue strongly suggests that other unknown factors within the MaoP regulon are required for full AMP resistance. The authors should address this and discuss what other HdfR-MaoP-dependent envelope modifications might be contributing to this phenotype.
8. The authors identify the FDA-approved drug flunixin as a chemical inhibitor that reduces maoP promoter activity in an HdfR-dependent manner, thereby sensitizing Y. pestis to AMPs. While an exciting finding, the molecular mechanism is entirely unexplored. It is unclear if flunixin directly binds to the HdfR protein to inhibit its transcriptional activation function, or if it acts upstream. Providing biochemical evidence of direct binding (e.g., using the purified Y. pestis HdfR in microscale thermophoresis or surface plasmon resonance assays) would substantially elevate the pharmacological relevance of this finding.
9. The full complementation of the mutant phenotype by E. coli HdfR and MaoP is particularly striking. In fact, this result appears to challenge, rather than support, the authors' broader hypothesis that the HdfR-MaoP regulatory hub evolved specifically in Y. pestis to coordinate adaptation between arthropod and mammalian hosts, given that E. coli does not share this dual-host lifestyle. What is the physiological function of the HdfR-MaoP system in E. coli and in other Enterobacteriaceae? A clearer discussion of its ancestral role would help determine whether Y. pestis has uniquely adapted a conserved regulatory circuit for vector-host transition, or whether the manuscript is overstating the degree of lifestyle-specific specialization.

Version 1:

Reviewer comments:

Reviewer #1

(Remarks to the Author)
I thank the authors for their careful and thoughtful revision of the manuscript. The revised version addresses my major concerns and substantially improves the clarity of the study, particularly with respect to the presentation of the regulatory model and the interpretation of the data. The authors have appropriately clarified several points that were previously unclear and have generally aligned their conclusions more closely with the evidence presented.

While some mechanistic questions regarding the HdfR–MaoP regulatory module remain open, I view these as opportunities for future investigation rather than deficiencies that limit the current study. The manuscript now provides sufficient support for its principal conclusions and represents a valuable contribution to our understanding of regulatory adaptation in Yersinia pestis.

I have no further major concerns and support publication of the manuscript in its current form.

Reviewer #3

(Remarks to the Author)
I appreciate the authors thoroughly addressed my comments in the revised version, and have no further comments.

Open Access This Peer Review File is licensed under a Creative Commons Attribution 4.0 International License, which permits use, sharing, adaptation, distribution and reproduction in any medium or format, as long as you give appropriate credit to the original author(s) and the source, provide a link to the Creative Commons license, and indicate if changes were made.
In cases where reviewers are anonymous, credit should be given to 'Anonymous Referee' and the source.
The images or other third party material in this Peer Review File are included in the article's Creative Commons license, unless indicated otherwise in a credit line to the material. If material is not included in the article's Creative Commons license and your intended use is not permitted by statutory regulation or exceeds the permitted use, you will need to obtain permission directly from the copyright holder.
To view a copy of this license, visit https://creativecommons.org/licenses/by/4.0/

We thank the reviewers for the careful and constructive evaluation of our manuscript. We have thoroughly revised the work to address the points raised, incorporating substantial new experimental data and refining the presentation of our findings. In our response, we have included the revised text (highlighted in brown for clarity) below each of our replies.

Reviewer #1 (Remarks to the Author):

Summary and significance

The authors describe a regulatory module in Yersinia pestis consisting of HdfR, a flea-induced transcriptional regulator, and MaoP, a nucleoid-associated protein. They propose that this HdfR–MaoP module coordinates adaptation across flea and mammalian host environments by promoting biofilm formation in the flea vector while altering outer membrane architecture during mammalian infection to support immuchne evasion. In this model, the circuit integrates environmental cues, particularly temperature, to drive context-specific phenotypic outputs. The authors further suggest that this regulatory module is conserved among enteric bacteria and demonstrate that it can be pharmacologically targeted through small-molecule inhibition of HdfR-dependent activation. Collectively, they argue that the HdfR–MaoP module represents an essential component of the vector-borne life cycle of Y. pestis and a broadly conserved adaptive strategy.

The idea that a conserved regulatory module links nucleoid-associated regulation with host-specific adaptation is conceptually compelling. If fully substantiated, the work would be of broad interest to the fields of bacterial pathogenesis, regulatory biology, host–pathogen interactions, and evolutionary microbiology. The proposed integration of environmental sensing with downstream regulatory outputs represents a potentially important conceptual advance. However, the mechanistic depth and integrative framework currently fall short of supporting the broader claims made regarding life-cycle coordination and evolutionary conservation.

Major Comments:

Despite the strong conceptual framework, the mechanistic evidence presented does not yet fully support the breadth of the claims regarding coordinated life-cycle regulation and rapid host transition. While many experiments are technically sound, several conclusions extend beyond what is directly demonstrated.

A key concern is the regulatory logic of the module. Transcriptional control of maoP by HdfR is presented as the central regulatory node, yet MaoP appears expressed in both flea and mammalian conditions without a clear explanation for how its activity is differentially modulated. It remains unclear whether maoP is upregulated in the flea as predicted and how differences in transcript levels alone account for distinct phenotypic outputs. If MaoP is present across conditions, additional regulatory layers—such as altered DNA binding, protein–protein interactions, localization, or post-translational modulation—may be required to explain environment-specific effects. Similarly, the proposed role of the module in rapid flea-to-mammal transition is not directly tested, as most analyses rely on steady-state temperature comparisons rather than acute transition models.

We thank the reviewer for this important comment, which raises key questions regarding the regulatory logic of the HdfR-MaoP module, the role of *maoP* expression across conditions, and the relevance of our data to host transition dynamics. We have carefully revised the manuscript and strengthened our experimental framework to address these points.

1. Regulatory logic and condition-dependent MaoP activity. To clarify how MaoP activity is differentially modulated, we have strengthened the experimental support for a hierarchical model in which HdfR controls *maoP* expression and MaoP acts as the central effector. We agree, however, that the HdfR-MaoP module should not be interpreted as a simple on/off switch in which MaoP is restricted to a single host-associated condition. Rather, our data support a quantitative and context-dependent model. New data demonstrate that *hdfR* expression increases at 37°C relative to 21°C (Extended Data 6e), and that shifting bacteria from 21°C to 37°C leads to an increase in MaoP protein abundance within 1 hour, whereas no such change occurs in bacteria maintained at 21°C (Extended Data 6f). This is consistent with our previous observations (Extended Data Fig. 10b), which also show increased MaoP levels at 37°C relative to 21°C. These results directly link environmental transitions to quantitative modulation of the module. Together, these data indicate that environmental cues modulate MaoP activity through HdfR-dependent quantitative control of its expression level. We further show that HdfR positively autoregulates its own expression and that MaoP negatively autoregulates *maoP* transcription, revealing feedback loops within the module (Extended Data 6c-d). For clarity, we have provided two new panels (Extended Data 6e-f) and their legends, and revised the manuscript to explicitly highlight these effects.

In addition, we initially showed that controlled expression of *maoP* in the absence of HdfR restores all tested phenotypes in a dose-dependent manner, demonstrating that MaoP is sufficient to drive the observed outputs once expressed (Figure 1h-j and Extended Data Fig. 4c-e). At the same time, these outputs are strongly condition-dependent, with distinct phenotypes and target genes expressed under flea-like versus mammalian conditions. This indicates that MaoP activity leads to distinct regulatory outcomes depending on the environmental context in which it operates. Thus, the distinct flea-like and mammalian-stage phenotypes do not require MaoP to be exclusively expressed in one condition, but are consistent with a model in which MaoP is present across conditions while its abundance and regulatory consequences differ according to temperature-dependent cellular states and target-gene contexts.

Taken together, these results support a model in which MaoP-dependent regulation is shaped by HdfR-mediated quantitative modulation of MaoP abundance, with autoregulatory feedback loops enabling fine-tuning rather than a simple on/off response. While additional regulatory, including changes in DNA-binding behavior, protein partners, localization or post-translational regulation, may further refine MaoP activity in vivo, our data support a parsimonious model in which MaoP abundance and environmental context account for the phenotypic outputs measured under the conditions tested.

Now it reads:

<u>*In the results section*</u>

- **Lines 89-99:** Focusing on the transcriptional regulator HdfR, we found that deletion of *hdfR* was sufficient to reproduce this phenotype when bacteria were exposed to serum at 37°C after being grown under either flea-like (21°C) or mammalian host-like (37°C), indicating that HdfR supports complement resistance both after flea-like temperature adaptation and under mammalian host-like conditions (Fig. 1a and Extended Data Fig. 2a). By contrast, in cross-temperature antimicrobial peptide assays designed to distinguish prior temperature adaptation from exposure temperature, the Δ*hdfR* mutant displayed heightened susceptibility to antimicrobial peptides only after growth at 37°C, regardless of the temperature used during peptide exposure (Fig. 1b and Extended Data Fig. 2b). Beyond these defects in innate immune resistance, the Δ*hdfR* mutant also exhibited reduced biofilm and exopolysaccharide production at 21°C (Fig. 1c and Extended Data Fig. 2c).
- **Lines 117-128:** In addition, HdfR positively autoregulated its own transcription, whereas MaoP negatively autoregulated *maoP* expression (Extended Data Fig. 5c,d), revealing feedback loops consistent with a self-stabilizing regulatory module capable of maintaining coordinated output across environmental conditions.

Consistent with the condition-dependent phenotypes controlled by the HdfR-MaoP module, *hdfR* expression was higher at 37°C than at 21°C (Extended Data Fig. 5e), estimated MaoP abundance was approximately threefold higher at 37°C than at 21°C on a per-cell basis, and shifting bacteria from 21°C to 37°C increased MaoP protein abundance over the first hour, whereas MaoP levels remained stable in bacteria maintained at 21°C (Extended Data Fig. 5f,g).

Together, these results define the HdfR-MaoP module as a quantitative regulatory hub that integrates flea-stage biofilm capacity with mammalian-stage resistance to innate immune defenses, thereby contributing to bacterial adaptation across distinct host-associated conditions.

In the Discussion

- **Lines 311-314**: At the core of this regulatory architecture lies MaoP, whose expression is controlled by the transcriptional regulator HdfR. In this hierarchy, HdfR links temperature-dependent host-associated cues to *maoP* expression, whereas MaoP acts downstream as a central effector, and its effects depend on both its expression level and the environmental condition.

In the Figure Legend

- **Lines 1191-1192**: Extended Data Figure 5. Hierarchical, autoregulation and temperature-dependent control of the HdfR-MaoP module.
- **Lines 1204-1208**: (E) Mean ± SD relative *hdfR* expression at 37°C compared with 21°C, measured by RT-qPCR (n = 3). (F) Mean ± SD estimated MaoP abundance per cell after incubation at 21°C or 37°C (n = 3), together with the standard curve used for MaoP quantification. (G) Mean ± SD relative MaoP abundance at different time points after temperature shifts from 21°C to 21°C or from 21°C to 37°C (n = 3).

2. Expression of *maoP* and interpretation of transcript levels. We clarify that the initial dataset used for candidate selection was based on genes previously reported as upregulated under flea-relevant conditions using microarray analyses, which served as an entry point for the screen rather than as definitive evidence of differential expression in our experimental system. In this context, *hdfR* was included among candidate genes, while *maoP* was subsequently identified through functional analyses downstream of HdfR. Importantly, *maoP* was not identified as flea-upregulated in the initial dataset , and we have revised the manuscript to avoid implying that *maoP* itself was selected on this basis. Our conclusions are therefore not based solely on transcript-level data, but on combined functional and protein-level evidence, including HdfR-dependent *maoP* promoter activity, MaoP protein abundance, autoregulatory feedback loops, temperature-dependent modulation of HdfR and MaoP levels, and dose-dependent phenotypic restoration upon controlled *maoP* expression. The manuscript has been revised accordingly to clarify these points.

Now it reads:

- **Lines 84-86:** To identify genes capable of supporting adaptation in both environments, we screened a library of *Y. pestis* deletion mutants targeting genes previously reported as upregulated in the flea for susceptibility to human serum.

3. Relevance to flea-to-mammal transition dynamics. We acknowledge that the original manuscript did not explicitly address the kinetics of acute host transitions. To address this point more directly, we have incorporated new experimental data showing that a shift from 21°C to 37°C increases MaoP protein abundance over the first hour, providing molecular evidence that the HdfR-MaoP module responds dynamically to a host-associated temperature shift (Extended Data 6g). In addition, our experimental design includes cross-temperature assays in which bacteria grown at one temperature were exposed to host-relevant stresses under another condition. These assays capture functional aspects of environmental transition, particularly the influence of prior physiological state on subsequent stress resistance. We have revised the manuscript to better distinguish cross-temperature

functional assays from molecular temperature-shift kinetics. Specifically, the revised text now clarifies that Δ*hdfR* strain susceptibility to antimicrobial peptides depends on prior growth at 37°C, regardless of the temperature used during peptide exposure, indicating that the HdfR-dependent phenotype reflects the physiological state established before stress exposure. In addition, we added new data showing that shifting bacteria from 21°C to 37°C increases MaoP protein abundance over the first hour, demonstrating that the module responds dynamically to a host-associated temperature shift.

Now it reads:

- **Lines 93-97:** By contrast, in cross-temperature antimicrobial peptide assays designed to distinguish prior temperature adaptation from exposure temperature, the Δ*hdfR* mutant displayed heightened susceptibility to antimicrobial peptides only after growth at 37°C, regardless of the temperature used during peptide exposure (Fig. 1b and Extended Data Fig. 2b).
- **Lines 123-125:** …and shifting bacteria from 21°C to 37°C increased MaoP protein abundance over the first hour, whereas MaoP levels remained stable in bacteria maintained at 21°C (Extended Data Fig. 5f,g).

The study uses an appropriate experimental toolkit, including transcriptional profiling, genetic perturbation, phenotypic assays, Hi-C analysis, and pharmacological inhibition. However, the work relies heavily on expression-level correlations, with limited direct mechanistic interrogation of MaoP's functional activity. Some interpretive claims—such as temperature-dependent mutant phenotypes, the exclusion of LOS pathway effects in complement sensitivity, the absence of significant Hi-C changes without quantitative metrics, and assertions of conservation and "core functional logic"—would benefit from clearer supporting data and definition. The pharmacological experiments also require stronger specificity controls and broader phenotypic validation.

We thank the reviewer for this constructive comment, which highlights the need to clearly distinguish between correlation-based observations and functionally supported conclusions, and to better define the mechanistic scope of the study. We have revised the manuscript accordingly to clarify these points and strengthen the experimental support.

Functional and mechanistic support beyond transcriptional correlations. Our conclusions are primarily supported by convergent genetic, functional, and biochemical evidence rather than expression-level correlations alone. Deletion of *hdfR* or *maoP* leads to similar defined phenotypic alterations, including defects in biofilm formation, serum resistance, and antimicrobial peptide resistance, which are restored upon genetic complementation. Critically, controlled expression of *maoP* in a Δ*hdfR* background restores all tested phenotypes in a dose-dependent manner (Figure 1h-j and Extended Data Fig. 4c-e), whereas *hdfR* overexpression does not rescue the Δ*maoP* mutant (Extended Data Fig. 6a-b), demonstrating that MaoP acts as the central effector of this regulatory module.

To further define the mechanistic basis of MaoP activity, we provide direct biochemical evidence beyond transcriptional profiling. Microscale thermophoresis and atomic force microscopy show that MaoP binds DNA in a sequence-independent manner and alters DNA conformation *in vitro* (Figure 4). Consistently, although ChIP-seq identifies MaoP-associated chromosomal regions *in vivo*, *in vitro* binding occurs independently of the MaoS site, indicating that MaoP does not function as a classical sequence-specific transcriptional regulator. This is consistent with a nucleoid-associated protein mode of action, in which regulatory outputs arise from context-dependent interactions with the chromosome. In addition, we did not detect interaction with RNA polymerase in bacterial two-hybrid assays, nor RNA enrichment in RIP experiments, and Hi-C analyses (now complemented by contact-probability decay as a function of genomic distance) did not reveal major local or large-scale changes

in chromosome organization under the conditions tested. Together, these results support a model in which MaoP acts as a DNA-binding, nucleoid-associated protein whose activity is linked genetically and functionally to the HdfR-dependent phenotypes

Now it reads

In the method section

- **Lines 852-856:** To quantitatively compare chromosome organization between strains, contact probability was calculated as a function of genomic distance from normalized Hi-C contact matrices. Contact-probability decay curves were compared between WT Δ*pla* and Δ*maoP* Δ*pla* strains to assess potential changes in short- and long-range chromosomal contacts.

In the result section

- **Lines 250-252:** MaoP is a small (14-kDa), non-highly basic DNA-binding protein, with an estimated abundance of approximately 1.4 x 10^4 and 4.4 x 10^4 molecules per cell at 21°C and 37°C respectively (Extended Data Fig. 5f).
- **Lines 255-257:** However, microscale thermophoresis measurements demonstrated that MaoP binds DNA without detectable sequence specificity under the conditions tested (Fig. 4b).
- **Lines 236-241:** Under the experimental conditions tested, MaoP did not interact with RNA polymerase in bacterial two-hybrid assays (RpoN, RpoB, RpoZ, RpoS, RpoE, RpoD) (Extended Data Fig. 9a), did not enrich for RNA above background in RIP experiments, and did not induce significant changes in local or global chromosome folding *in vivo*, as assessed by Hi-C contact maps and contact-probability decay as a function of genomic distance (Fig. 4a and Extended Data Fig. 9b).

In the discussion section

- **Lines 323-324:** By contrast, Hi-C contact maps and contact-probability decay analyses did not reveal major changes in chromosome folding *in vivo*.

In the Legend Figure section

- **Lines 1244-1253: Extended Data Figure 9. MaoP does not detectably interact with RNA polymerase or alter large-scale chromosome organization.** (**A**) Bacterial two-hybrid analysis testing interactions between MaoP and RNA polymerase subunits or sigma factors. Representative colonies are shown for the indicated combinations. The Zip-Zip pair was used as a positive control. No interaction was detected between MaoP and the tested RNA polymerase components under these conditions. (**B**) Contact-probability decay as a function of genomic distance calculated from normalized Hi-C contact matrices of WT Δ*pla* and Δ*maoP* Δ*pla* strains grown at 37°C. Curves are shown separately for chromosome and plasmid contacts. No major difference in contact-probability decay was observed between WT and Δ*maoP*, supporting the absence of detectable large-scale chromosome organization defects under the conditions tested.

Clarification of specific interpretive points. We have refined several aspects of the manuscript to more precisely reflect the scope of our data. Temperature-dependent phenotypes are now explicitly interpreted in the context of cross-temperature assays, in which bacteria are pre-adapted to one temperature before exposure to host-relevant stresses at either the same or a different temperature. Regarding LOS-related effects, we now indicate that while our data do not support major global alterations in LOS biosynthesis as the primary driver of the observed complement-sensitivity phenotype, more subtle or localized changes cannot be excluded. Similarly, our Hi-C analyses, now complemented by contact-probability decay as a function of genomic distance, did not reveal detectable local or large-scale changes in chromosome organization at the resolution tested.

We have also refined our description of conservation and replaced the broad term "core functional logic" with a more explicit framework distinguishing conserved genomic organization, functional interchangeability of HdfR and MaoP, and species- or niche-specific downstream outputs. Specifically, we now distinguish between conservation of the *hdfR*-*maoP* genomic organization and conservation of their functional relationship. Comparative analyses indicate that the *hdfR*-*maoP* locus is conserved across representative Enterobacterales, supporting the idea that this regulatory architecture is evolutionarily maintained. Importantly, by extending our analysis to *E. coli*, we now show that HdfR is required for biofilm formation in this species, and that this phenotype can be restored by expression of the *Y. pestis hdfR* gene. We further show that deletion of *maoP* in *E. coli* leads to a biofilm defect that is complemented by the *Y. pestis maoP* gene, demonstrating functional conservation of MaoP across species. Together, the conserved genomic context and the functional interchangeability observed for HdfR and MaoP support the existence of a shared regulatory framework for biofilm-associated regulation, while indicating that additional downstream outputs may be species- or niche-specific. We have provided a new figure (Extended Data Fig. 12i) and revised the manuscript accordingly to clarify this distinction and avoid overgeneralization.

Now it reads:

Regarding the temperature-dependent mutant phenotypes

- **Lines 89-99:** Focusing on the transcriptional regulator HdfR, we found that deletion of *hdfR* was sufficient to reproduce this phenotype when bacteria were exposed to serum at 37°C after being grown under either flea-like (21°C) or mammalian host-like (37°C), indicating that HdfR supports complement resistance both after flea-like temperature adaptation and under mammalian host-like conditions (Fig. 1a and Extended Data Fig. 2a). By contrast, in cross-temperature antimicrobial peptide assays designed to distinguish prior temperature adaptation from exposure temperature, the Δ*hdfR* mutant displayed heightened susceptibility to antimicrobial peptides only after growth at 37°C, regardless of the temperature used during peptide exposure (Fig. 1b and Extended Data Fig. 2b). Beyond these defects in innate immune resistance, the Δ*hdfR* mutant also exhibited reduced biofilm and exopolysaccharide production at 21°C (Fig. 1c and Extended Data Fig. 2c).

- **Lines 117-125:** In addition, HdfR positively autoregulated its own transcription, whereas MaoP negatively autoregulated *maoP* expression (Extended Data Fig. 5c,d), revealing feedback loops consistent with a self-stabilizing regulatory module capable of maintaining coordinated output across environmental conditions.

  Consistent with the condition-dependent phenotypes controlled by the HdfR-MaoP module, *hdfR* expression was higher at 37°C than at 21°C (Extended Data Fig. 5e), estimated MaoP abundance was approximately threefold higher at 37°C than at 21°C on a per-cell basis, and shifting bacteria from 21°C to 37°C increased MaoP protein abundance over the first hour, whereas MaoP levels remained stable in bacteria maintained at 21°C (Extended Data Fig. 5f,g).

Regarding the exclusion of LOS pathway effects in complement sensitivity

- **Lines 192-195:** By contrast, changes in the regulation of LOS biosynthesis pathways were unlikely to fully account for the complement sensitivity of HdfR-MaoP-deficient strains, because complement sensitivity was observed after growth at both 21°C and 37°C, whereas reduced *gmhA*/*rfaD* expression and detectable LOS defects were observed primarily at 37°C.

- **Lines 210-215:** It reinforces LOS biosynthesis at mammalian host temperature to limit antimicrobial peptide susceptibility, while restricting complement activation through a distinct mechanism that limits recognition by lectin and classical pathway sensors. Although subtle or localized LOS alterations cannot be excluded, this dual control ensures continuity of vector-stage transmission while enabling survival in the mammalian host.

Regarding, the absence of significant Hi-C changes without quantitative metrics

- **Lines 239-241:** …and did not induce significant changes in local or global chromosome folding *in vivo*, as assessed by Hi-C contact maps and contact-probability decay as a function of genomic distance (Fig. 4a and Extended Data Fig. 9b).

- **Lines 852-856:** To quantitatively compare chromosome organization between strains, contact probability was calculated as a function of genomic distance from normalized Hi-C contact matrices. Contact-probability decay curves were compared between WT Δ*pla* and Δ*maoP* Δ*pla* strains to assess potential changes in short- and long-range chromosomal contacts.

Regarding the assertions of conservation and "core functional logic

In the result section

- **Lines 276-282:** Conversely, deletion of *hdfR* or *maoP* in *E. coli* impaired biofilm formation, and expression of the corresponding *Y. pestis* homolog restored this defect, indicating reciprocal functional conservation for this shared biofilm-associated output (Extended Data Fig. 12i). These findings indicate that the HdfR-MaoP module constitutes an evolutionarily conserved regulatory unit, with conserved genomic organization, functional interchangeability, and shared biofilm-associated regulatory activity, while immune-resistance outputs may reflect species- and niche-specific deployment of this regulatory scaffold.

In the discussion

- **Lines 380-392:** Our data connect these observations by showing that the organization and regulatory hierarchy of HdfR-MaoP have been retained despite substantial sequence divergence. The functional conservation of this scaffold is supported by prior evidence that MaoP expression is HdfR-dependent in *E. coli*[45], and by our cross-species complementation experiments showing that HdfR and MaoP from *E. coli* restore all tested phenotypes in the corresponding *Y. pestis* mutants. Conversely, HdfR and MaoP from *Y. pestis* restore biofilm formation in the corresponding *E. coli* mutants, despite the absence of the HdfR-MaoP-regulated *Y. pestis hmsHFRS* biofilm genes in *E. coli*. Together, these findings suggest that HdfR-MaoP is an ancient regulatory module whose outputs have diversified across ecological contexts, with downstream outputs shaped by species-specific physiology and niche-associated pressures. In *Y. pestis*, this scaffold appears to have been integrated into regulatory programs that coordinate flea-borne transmission and mammalian host adaptation.

In the Figure Legend

- **Lines 1298-1306: Extended Data Figure 12. Conservation of the HdfR-MaoP module across diverse bacterial species and cross-species functional complementation between *Y. pestis* and *E. coli*. (A)** Pairwise sequence alignments of HdfR and MaoP from *Y. pestis* and *E. coli*. Conserved residues are indicated, with the helix-turn-helix (HTH) DNA-binding domain of HdfR highlighted in green and the predicted basic central region of MaoP highlighted in blue. (**B**) Genomic synteny of the *hdfR-maoP* gene pair in representative Gammaproteobacteria, illustrating conserved gene organization and orientation across species. (**C-D**) Mean ± SD survival of *Y. pestis* WT, Δ*hdfR*, and Δ*hdfR* complemented with 6xHis-tagged *hdfR* from *E. coli* (-c_{Ec})

- **Lines 1311-1313:** (**E**) Biofilm formation after a 24-h incubation at 21°C by *Y. pestis* WT, Δ*hdfR* and Δ*hdfR* complemented with 6xHis-tagged *hdfR* from *E. coli*, expressed as a percentage relative to WT (mean ± SD, n = 3; *, p < 0.05).

- **Lines 1320-1322:** (**I**) Biofilm formation after a 24-h incubation at 37°C by *E. coli* WT, Δ*hdfR*, and Δ*maoP* strains, with the mutant strains complemented with their respective *Y. pestis* orthologs (-C_{Yp}), expressed as a percentage relative to WT (mean ± SD, n = 3; , p < 0.05).

Pharmacological specificity and phenotypic scope. We have strengthened the interpretation of the pharmacological data by incorporating additional controls. First, we now provide direct biochemical evidence that flunixin interacts with purified HdfR, as demonstrated by microscale thermophoresis, with a dissociation constant (Kd) of approximately 1 µM. The effect of flunixin on antimicrobial peptide sensitivity is strictly HdfR-dependent, as no additional effect is observed in the Δ*hdfR* mutant. Conversely, heterologous expression of *E. coli hdfR* restores flunixin-dependent antimicrobial peptide sensitization, further supporting an HdfR-dependent mode of action. We also tested its impact on biofilm formation and did not observe a robust or reproducible effect under our conditions. Notably, biofilm formation was assessed at 21°C, a condition under which flunixin does not inhibit *maoP* transcription, providing a potential explanation for the absence of effect on this phenotype. Accordingly, we restrict our conclusions to flunixin-mediated perturbation of an HdfR-dependent pathway affecting antimicrobial peptide resistance, rather than presenting it as a general inhibitor of all HdfR-MaoP-dependent phenotypes. These data strengthen the specificity of the pharmacological perturbation while defining its phenotypic scope. We have provided a new figure 5 and revised the manuscript.

Now it reads:

- **Lines 285-301:** To assess whether the HdfR-MaoP regulatory module is amenable to chemical perturbation, we monitored maoP promoter activity in wild-type Y. pestis in the presence of a library of FDA-approved compounds. This screen identified flunixin as a compound capable of reducing *maoP* promoter activity in a dose-dependent manner at 37°C, with up to a twofold decrease under the conditions tested (Fig. 5a). At the highest concentration active at 37°C, flunixin did not reduce *maoP* promoter activity at 21°C (Fig. 5b). Consistent with this temperature-restricted transcriptional effect, flunixin exposure did not reproducibly impair biofilm formation under flea-like conditions (21°C) (Fig. 5c), but increased susceptibility of *Y. pestis* to antimicrobial peptides under mammalian host-like conditions (37°C) (Fig. 5d,e). This peptide-sensitizing effect required HdfR, as flunixin did not further increase Δ*hdfR* susceptibility, even at polymyxin concentrations where mutant survival remained above the lower detection plateau (Fig. 5d,e). In line with this genetic requirement, heterologous expression of *E. coli hdfR* restored flunixin-dependent peptide sensitization (Fig. 5d,e). Furthermore, at the biochemical level, microscale thermophoresis using purified HdfR showed direct binding of flunixin to HdfR, with an apparent Kd of approximately 1 µM (Fig. 5f). Together, these results indicate that flunixin provides a chemical entry point to pharmacologically perturb the HdfR-MaoP regulatory module through a conserved HdfR-dependent mechanism.

Now it reads in the Figure Legends:

- **Lines 628-642:** (**A-B**) *maoP* promoter activity in the *Y. pestis* wild-type strain carrying a chromosomal P_{maoP}-lux reporter after a 4 hours incubation at 37°C in the presence of increasing concentrations of flunixin (A) or at 21°C or 37°C in the presence of 25 µM flunixin (B), expressed as relative luminescence units (RLU), normalized to the OD_{595}. (**C**). Biofilm formation after a 24-h incubation at 21°C, expressed as a percentage relative to WT (n = 2) in the presence of 25 µM flunixin (**D-E**) Survival of WT, Δ*hdfR* and Δ*hdfR* complemented with 6xHis-tagged *hdfR* from *E. coli* (-c_{Ec}) strains after exposure to flunixin (25 µM) and increasing concentrations of polymyxin B (D) or fixed concentrations of polymyxin B, 0 µg/mL, 0.019 µg/mL or 0.6 µg/mL (E), alone or in combination, in the presence of the DMSO vehicle control (Mock) (n = 4; *, $p < 0.05$). Survival was quantified as Log_{10} RLU (N/N_0) (D) or RLU (N/N_0) (E) using bacteria grown and exposed at 37°C, where N corresponds to luminescence after exposure and N_0 to luminescence before exposure. (**F**) Flunixin binding assessed by MST using purified HdfR. Fraction bound is plotted as a function of flunixin concentration. Data points represent mean ± SD from three independent measurements, and solid lines indicate fits to a single-site binding model.

Overall, the manuscript is ambitious and generally well written, but several claims are framed more strongly than the supporting evidence warrants. Clearer articulation of regulatory hierarchy, direct testing of transition dynamics, stronger quantitative rigor, and deeper contextualization within the

literature on nucleoid-associated proteins and bacterial adaptation would substantially strengthen the work. In its current form, the manuscript would benefit from substantial mechanistic clarification and additional validation before the central claims can be fully supported.

We sincerely thank the reviewer for this thoughtful and constructive evaluation, which has prompted us to critically reassess the clarity, precision, and alignment between our conclusions and the supporting evidence. We have therefore revised the manuscript along four main directions, including careful moderation of claims where appropriate to avoid overinterpretation.

1. **Clarification of the regulatory hierarchy.** We now explicitly define HdfR as an input-responsive regulator controlling *maoP* expression, with MaoP as the central downstream effector. This framework is supported by integrated functional evidence, including temperature-dependent modulation of *hdfR* expression and MaoP abundance, the increase in MaoP levels following a 21°C-to-37°C temperature shift, autoregulatory feedback, and dose-dependent phenotypic rescue upon controlled *maoP* expression.

2. **Refinement of transition dynamics.** We have clarified the interpretation of host-associated transitions by distinguishing condition-dependent phenotypes from temperature-shift responsiveness and broader host-transition dynamics. In addition, we incorporated new data demonstrating that a temperature shift from 21°C to 37°C increases MaoP protein abundance, providing direct evidence that the HdfR-MaoP module responds dynamically to a host-associated temperature cue.

3. **Improved quantitative rigor and data interpretation.** We have strengthened the analysis and presentation of specific datasets to ensure that all conclusions are fully supported. In particular, temperature-dependent phenotypes are now interpreted more explicitly in light of cross-temperature assays, Hi-C results are complemented by contact-probability decay as a function of genomic distance, and statements regarding LOS-related contributions to complement sensitivity have been moderated to avoid over-interpretation while acknowledging potential subtle or localized effects.

4. **Strengthened conceptual framework and contextualization.** We have refined the definition of a conserved regulatory architecture and expanded our analysis to *E. coli*, where we demonstrate functional interchangeability of HdfR and MaoP homologs for shared biofilm-associated regulation. We have also expanded the discussion to better position MaoP within the broader literature on nucleoid-associated proteins, emphasizing its high abundance, broad DNA-binding activity, chromosomal occupancy, effects on DNA conformation, and absence of detectable large-scale chromosome reorganization under the conditions tested. This revised framing distinguishes MaoP from classical sequence-specific transcription factors while placing it within the broader repertoire of bacterial chromosomal regulators involved in environmental adaptation.

Overall, these revisions ensure that the central conclusions of the study are supported by the experimental evidence, while maintaining the scope and significance of the work without overstating its implications.

Minor/Line Comments:

Lines 83-85: Was maoP also identified as being upregulated in the flea? The proposed model would seemingly predict this to be true but is not seemingly specified here or elsewhere.

maoP was not identified as upregulated under flea conditions in the transcriptomic dataset used for candidate selection. Rather, *hdfR* was included among the flea-upregulated candidates, and *maoP* was subsequently identified as the major downstream effector of HdfR through functional and regulatory analyses. Importantly, our model does not require *maoP* to be specifically upregulated in the flea, as its regulatory output is determined by HdfR-dependent control, MaoP abundance, and environmental context rather than transcript levels alone. We have revised the manuscript to clarify this point and avoid any ambiguity.

Now it reads:

- **Lines 84-86:** To identify genes capable of supporting adaptation in both environments, we screened a library of *Y. pestis* deletion mutants targeting genes previously reported as upregulated in the flea for susceptibility to human serum.

And the reader can read later :

- **Lines 108-109:** ….inspection of the dataset suggested that HdfR primarily acts through the activation of a single downstream factor, MaoP.

Line 100: The statement "Despite this broad impact…" implies that MaoP is the primary downstream factor, but this is not obvious from the presentation.

We agree that the initial wording could have prematurely suggested a strong hierarchical relationship. We have revised the text to avoid implying that MaoP is the primary downstream effector at this stage and to introduce this concept more progressively. Specifically, we now write that inspection of the proteomic dataset "suggested" that HdfR primarily acts through MaoP, and we reserve the stronger conclusion, "These data establish MaoP as the indispensable downstream effector of the HdfR regulon" (Lines 115-116) for the subsequent genetic and functional evidence, including Δ*maoP* phenocopy, dose-dependent rescue by controlled *maoP* expression, and the inability of *hdfR* overexpression to rescue the Δ*maoP* mutant.

Now it reads:

- **Lines 107-109:** Despite this broad impact, inspection of the dataset suggested that HdfR primarily acts through the activation of a single downstream factor, MaoP.

Line 100: Proteomics data is shown for 37°C but screen identified HdfR as being upregulated in the flea. So it was not clear how examination of the proteomics at the mammalian temperature was the most-relevant condition to examine.

Proteomic analyses were performed at both 21°C and 37°C (Extended Data Fig. 2d). While HdfR-dependent changes were detected under both conditions, a larger number of differentially produced proteins was observed at 37°C, providing greater resolution to identify candidate downstream factors. For this reason, the 37°C dataset was shown in the main figure, whereas the comparison across both temperatures is provided in Extended Data Fig. 2d. We have revised the text to clarify this point.

Now it reads:

- **Lines 102-107:** Corroborating the pleiotropic role of HdfR and providing mechanistic insight, quantitative proteomic analyses performed at both 21°C and 37°C uncovered extensive HdfR-dependent changes in

the protein landscape across temperatures, with 319 proteins differentially produced (Fig. 1d and Extended Data Fig. 2d). The majority of these proteins were less abundant in the Δ*hdfR* mutant, notably those involved in central metabolism and membrane architecture, with stronger effects observed at 37°C (Extended Data Fig. 2d and 3).

Line 158: Extended Data Figure 6a is cited as supporting temperature-dependence of maoP mutant, but supporting data is shown for hdfR mutant and it is not clear is this faithfully applies also to the maoP mutant. Does hdfR mutation also reduce LOS production at 37°C?

Extended Data Fig. 6a originally showed LOS profiles for the Δ*hdfR* mutant at 21°C, whereas LOS alterations for the Δ*maoP* mutant at 37°C were shown in Fig. 3b. We agree that this could create ambiguity regarding whether the LOS phenotype observed in Δ*maoP* also applied to Δ*hdfR*, despite the strong phenotypic concordance between the two mutants. To address this directly, we added the corresponding LOS analysis for Δ*hdfR* grown at 37°C to the revised Extended Data Fig. 7a. This analysis shows that Δ*hdfR* exhibits reduced LOS production at 37°C but not at 21°C, in agreement with the proteomic reduction of GmhA and RfaD. Consistent with MaoP acting downstream of HdfR, the Δ*maoP* mutant similarly displays reduced *gmhA* and *rfaD* promoter activity, decreased LOS production, and increased outer membrane permeability at 37°C. We have revised the Results to present the Δ*hdfR* LOS analysis first, followed by the Δ*maoP* mechanistic analysis, thereby clarifying the hierarchy and temperature dependence of this envelope phenotype.

Now it reads:

- **Lines 173-177:** In agreement with these proteomic data, LOS analysis of the Δ*hdfR* mutant showed reduced LOS production at 37°C but not at 21°C (Extended Data Fig. 7a). Consistent with MaoP acting downstream of HdfR, deletion of *maoP* resulted in a reduction in *gmhA* and *rfaD* promoter activity at 37°C (Fig. 3a), correlating with decreased LOS production (Fig. 3b) and increased outer membrane permeability (Fig. 3c).

Now it reads in the Figure legend

- **Lines 1220-1229: Extended Data Figure 7. MaoP- and HdfR-dependent phenotypes are not associated with global alterations in LOS biosynthesis, complement inhibitor recruitment, or individual omics-based candidate genes.** (**A**) DOC-PAGE analysis of LOS from WT, Δ*hdfR* and complemented (-c) strains grown at 21°C or 37°C. Equal amounts of bacterial material were loaded. No difference was observed under flea-like (21°C) conditions, whereas a decreases in LOS quantity was observed in Δ*hdfR* under mammalian host-like (37°C) conditions. (**B**) Mean ± SD promoter activity of genes involved in LOS biosynthesis and export pathways, as well as canonical lipid A modification pathways associated with polymyxin resistance, in WT and Δ*maoP* strains cultured at 37°C and carrying *gfp* reporters, expressed as relative fluorescence units (RFU) relative to WT (n = 2).

- **Lines 1234-1238:** (E) Mean ± SD survival of WT, Δ*hdfR*, and mutants lacking genes identified as putatively regulated by the HdfR-MaoP module in the present omics analysis, after exposure to normal human serum. Survival was quantified as Log_{10} RLU (N/N_0) using bacteria grown at 21°C and then exposed to serum at 37°C for 180 min, where N corresponds to luminescence after exposure and N_0 to luminescence before exposure.

Line 165: The conclusions presented in statement "By contrast…" are not obvious. It is not clear how LOS pathway perturbation was ruled out as an underlying cause of complement sensitivity.

We agree that this point was not sufficiently explicit. Complement sensitivity in HdfR-MaoP-deficient strains is observed after growth at both 21°C and 37°C, whereas reduced *gmhA*/*rfaD* expression and detectable LOS defects were observed primarily at 37°C, indicating that these temperature-dependent

LOS changes alone cannot account for the phenotype. In addition, we did not detect evidence for a global defect in LOS biosynthesis in the Δ*maoP* mutant, as LOS biosynthesis/export gene expression and LpxC production were not reduced compared to WT (Extended Data Fig. 7b,c). Finally, complement-deposition assays showed increased binding of MBL and C1q to the Δ*maoP* mutant (Figure 3i), supporting increased recognition by lectin and classical pathway sensors as a distinct contributor to complement activation. We have revised the manuscript to clarify this point and now explicitly acknowledge that subtle or localized LOS alterations cannot be excluded.

Now it reads

- **Lines 192-195:** By contrast, changes in the regulation of LOS biosynthesis pathways were unlikely to fully account for the complement sensitivity of HdfR-MaoP-deficient strains, because complement sensitivity was observed after growth at both 21°C and 37°C, whereas reduced *gmhA*/*rfaD* expression and detectable LOS defects were observed primarily at 37°C.
- **Lines 210-215:** It reinforces LOS biosynthesis at mammalian host temperature to limit antimicrobial peptide susceptibility, while restricting complement activation through a distinct mechanism that limits recognition by lectin and classical pathway sensors. Although subtle or localized LOS alterations cannot be excluded, this dual control ensures continuity of vector-stage transmission while enabling survival in the mammalian host.

Lines 186-187: Data is not shown for RIP and two-hybrid assays which are important findings given the systematic evaluation of MaoP function and its proposed role in chromosomal origin maintenance.

The bacterial two-hybrid data are now explicitly included in the revised manuscript (Extended Data Fig. 9a), showing no detectable interaction between MaoP and the tested RNA polymerase subunits or sigma factors under the conditions tested. Regarding the RIP experiments, we did not detect RNA enrichment associated with MaoP above background under our experimental conditions, arguing against a stable MaoP-RNA association in this assay. We have revised the text accordingly and added the missing two-hybrid data to avoid ambiguity.

Now it reads:

- **Lines 236-239:** Under the experimental conditions tested, MaoP did not interact with RNA polymerase in bacterial two-hybrid assays (RpoN, RpoB, RpoZ, RpoS, RpoE, RpoD) (Extended Data Fig. 9a), did not enrich for RNA above background in RIP experiments…

Line 186: It is stated that there are no significant changes to Hi-C map despite apparent signatures on the comparative interaction map. No metrics were provided to quantify changes, which made it hard to evaluate this claim.

Our initial intention was to indicate that MaoP does not induce major local or large-scale reorganization of chromosome architecture under the conditions tested. We agree that this conclusion required quantitative support. We have therefore added contact-probability decay analysis as a function of genomic distance (Extended Data Fig. 9b). This analysis showed highly similar profiles between WT and Δ*maoP* strains across genomic scales, supporting the absence of detectable large-scale chromosome reorganization. We have revised the manuscript to clarify that, although modest local variations may be visible on comparative contact maps, the data do not support major changes in chromosome architecture under the conditions tested.

Now it reads

In the method section

- **Lines 852-856:** To quantitatively compare chromosome organization between strains, contact probability was calculated as a function of genomic distance from normalized Hi-C contact matrices. Contact-probability decay curves were compared between WT Δ*pla* and Δ*maoP* Δ*pla* strains to assess potential changes in short- and long-range chromosomal contacts.

In the result section

- **Lines 239-241:** …did not induce significant changes in local or global chromosome folding *in vivo*, as assessed by Hi-C contact maps and contact-probability decay as a function of genomic distance (Fig. 4a and Extended Data Fig. 9b).

In the discussion

- **Lines 323-324:** By contrast, Hi-C contact maps and contact-probability decay analyses did not reveal major changes in chromosome folding *in vivo*.

Lines 188-189: Would be useful to be explicit about location of MaoS site. It was not immediately obvious that this was present in the intergenic region between hdfR and maoP until a thorough consultation of previous work.

We now explicitly indicate that the MaoS site is located in the intergenic region between *hdfR* and *maoP*. In addition, Extended Data Fig. 10 has been revised to show the position of the MaoS site within the conserved genomic context of the *hdfR*-*maoP* locus in representative Gammaproteobacteria.

Now it reads:

- **Lines 241-243:** Deletion of the MaoS site, located in the intergenic region between *hdfR* and *maoP*, abolished endogenous *maoP* transcription and….

Line 191: Not clear what is meant by conservation of MaoP function in this context. Does the author mean to imply that function of MaoP is independent of the MaoS site? My guess is that this is related to downstream arguments for the protein product being the primary driver of MaoP-dependent phenotypes.

MaoP-dependent phenotypes do not require the MaoS site once *maoP* expression is restored under the conditions tested. Expression of *maoP* from an inducible promoter in a ΔMaoS background restores the tested phenotypes (Extended Data Fig. 10f–k), indicating that these effects are driven by the MaoP protein product rather than by the MaoS DNA element itself. The manuscript has been revised to clarify this point.

Now it reads

- **Lines 244-248:** However, the MaoS site was not required for MaoP-dependent phenotypes once *maoP* expression was restored, as expression of *maoP* from an inducible promoter in a ΔMaoS site background fully restored resistance to antimicrobial peptides and serum, as well as biofilm formation (Extended Data Fig. 10f-k).

Line 231: What is the baseline sensitivity in this assay? Is it possible that the lack of additive sensitivity in the *hdfR* mutant is due to a floor in the sensitivity? Additionally, data is only shown for one promoter. It is formally possible that the drug is not specific to a single promoter, which would alter the

interpretation of these findings. It would have also been more compelling to see how this drug affects other relevant phenotypes shown such as biofilm formation.

The baseline sensitivity has now been clarified in the revised figure. Importantly, we now show that the absence of an additional flunixin effect in the Δ*hdfR* mutant is observed not only at high polymyxin concentrations, where survival approaches the lower plateau, but also at lower concentrations, where survival remains above this level. This argues against a floor effect and supports an HdfR-dependent mechanism. We agree that a single promoter assay does not establish pathway specificity on its own. However, specificity is supported by genetic analyses showing that flunixin has no additional effect in the Δ*hdfR* mutant and that flunixin-dependent peptide sensitization is restored upon heterologous expression of *E. coli hdfR*. These results indicate that the compound acts through a conserved HdfR-dependent pathway rather than through a general toxic effect. This interpretation is further supported by new biochemical data demonstrating direct binding of flunixin to HdfR (Kd ~1 µM).

We also assessed an additional HdfR-MaoP-dependent phenotype and did not observe a reproducible effect of flunixin on biofilm formation. Notably, biofilm formation was assessed at 21°C, a condition under which flunixin does not inhibit *maoP* transcription, which may account for the absence of effect on this phenotype. We have therefore revised the manuscript to limit our conclusions accordingly and now describe flunixin as providing a chemical entry point to pharmacologically perturb the HdfR-MaoP module through a conserved HdfR-dependent mechanism, with the phenotypic effect demonstrated here restricted to antimicrobial peptide sensitization. We have provided a new figure and revised the manuscript accordingly.

Now it reads

- **Lines 285-301:** To assess whether the HdfR-MaoP regulatory module is amenable to chemical perturbation, we monitored maoP promoter activity in wild-type Y. pestis in the presence of a library of FDA-approved compounds. This screen identified flunixin as a compound capable of reducing *maoP* promoter activity in a dose-dependent manner at 37°C, with up to a twofold decrease under the conditions tested (Fig. 5a). At the highest concentration active at 37°C, flunixin did not reduce *maoP* promoter activity at 21°C (Fig. 5b). Consistent with this temperature-restricted transcriptional effect, flunixin exposure did not reproducibly impair biofilm formation under flea-like conditions (21°C) (Fig. 5c), but increased susceptibility of *Y. pestis* to antimicrobial peptides under mammalian host-like conditions (37°C) (Fig. 5d,e). This peptide-sensitizing effect required HdfR, as flunixin did not further increase Δ*hdfR* susceptibility, even at polymyxin concentrations where mutant survival remained above the lower detection plateau (Fig. 5d,e). In line with this genetic requirement, heterologous expression of *E. coli hdfR* restored flunixin-dependent peptide sensitization (Fig. 5d,e). Furthermore, at the biochemical level, microscale thermophoresis using purified HdfR showed direct binding of flunixin to HdfR, with an apparent Kd of approximately 1 µM (Fig. 5f). Together, these results indicate that flunixin provides a chemical entry point to pharmacologically perturb the HdfR-MaoP regulatory module through a conserved HdfR-dependent mechanism.

Now it reads in the Figure Legends:

- **Lines 628-642:** (**A-B**) *maoP* promoter activity in the *Y. pestis* wild-type strain carrying a chromosomal P_{maoP}-lux reporter after a 4 hours incubation at 37°C in the presence of increasing concentrations of flunixin (A) or at 21°C or 37°C in the presence of 25 µM flunixin (B), expressed as relative luminescence units (RLU), normalized to the OD_{595}. (**C**). Biofilm formation after a 24-h incubation at 21°C, expressed as a percentage relative to WT (n = 2) in the presence of 25 µM flunixin (**D-E**) Survival of WT, Δ*hdfR* and Δ*hdfR* complemented with 6xHis-tagged *hdfR* from *E. coli* (-c_{Ec}) strains after exposure to flunixin (25 µM) and increasing concentrations of polymyxin B (D) or fixed concentrations of polymyxin B, 0 µg/mL, 0.019 µg/mL or 0.6 µg/mL (E), alone or in combination, in the presence of the DMSO vehicle control (Mock) (n = 4; *, $p < 0.05$). Survival was quantified as Log_{10} RLU (N/N_0) (D) or RLU (N/N_0) (E) using bacteria grown and exposed at 37°C, where N corresponds to luminescence after exposure and N_0 to luminescence before exposure. (**F**) Flunixin binding assessed by MST using purified HdfR. Fraction bound is plotted as

a function of flunixin concentration. Data points represent mean ± SD from three independent measurements, and solid lines indicate fits to a single-site binding model.

Line 262: Not clear what characteristics of canonical nucleoid-associated proteins are not displayed by MaoP. These distinctions are presented as central to the novelty of this module, so these differences should be more explicitly highlighted.

We agree that the previous wording was insufficiently precise. We have removed the statement that MaoP does not display all characteristic features of canonical nucleoid-associated proteins, because this formulation did not clearly define which features were being considered. Instead, we now position MaoP within the functional spectrum of nucleoid-associated proteins by explicitly highlighting the properties it shares with this class of regulators: small size, high cellular abundance, a basic central region, broad DNA-binding activity without detectable sequence specificity under the conditions tested, discrete chromosomal occupancy, broad regulatory consequences, local effects on DNA conformation in vitro, and autoregulation. We also clarify that MaoP differs from classical sequence-specific transcription factors and does not behave as a global chromosome-folding factor detectable by Hi-C or contact-probability decay. The revised Discussion therefore presents MaoP as an architectural DNA-binding protein whose distinctive feature in this system lies in the integration of broad DNA-binding activity into an HdfR-controlled, temperature-responsive regulatory module, rather than as a factor defined by a vague departure from canonical NAPs.

Now it reads:

- **Lines 320-339:** Our mechanistic analyses clarify MaoP's molecular function within this framework. MaoP is a small, highly abundant protein with a basic central region and the capacity to autoregulate its own expression. It binds DNA without detectable sequence specificity and alters DNA conformation in vitro, as shown by MST and AFM. By contrast, Hi-C contact maps and contact-probability decay analyses did not reveal major changes in chromosome folding *in vivo*. This difference likely reflects the different scales probed by the two approaches, with AFM detecting local single-molecule DNA conformational changes, whereas Hi-C reports population-averaged chromosomal contacts at kilobase resolution, as well as potentially dynamic or heterogeneous MaoP-DNA interactions, as previously reported for H-NS[33]. Thus, transient, heterogeneous, or sub-kilobase MaoP-DNA interactions may not produce detectable changes in global Hi-C profiles. This scale-dependent behavior is consistent with MaoP acting as a local architectural DNA-binding factor rather than as a global nucleoid organizer, a role classically associated with abundant NAPs such as HU or IHF [34,35]. Together, these biochemical and functional properties place MaoP within the NAP functional spectrum. However, rather than acting as an autonomous global nucleoid organizer, MaoP appears to function as a quantitatively tuned architectural effector embedded in an HdfR-controlled, temperature-responsive regulatory module. How MaoP functionally interfaces with established nucleoid-associated proteins and DNA-binding regulators remains to be defined. These factors may act together with MaoP to modulate virulence-associated gene expression under specific conditions.

Line 264: Transcriptional regulation of maoP by HdfR is presented as a key aspect of the HdfR-MaoP module, but it is unclear how this is specifically coordinating activity with respect to the module. It appears that MaoP is presented and expressed (albeit possibly at different levels) across temperatures, but is not clear how this level of regulation explains differences in apparent activities of this module in both conditions. It appears that this is an important aspect of the module that is missing from the data presented.

Our data indicate that HdfR-dependent regulation of *maoP* quantitatively modulates MaoP levels across conditions, rather than acting as a simple on/off switch. We have added new results showing that *hdfR* expression is higher at 37°C than at 21°C, that estimated MaoP abundance is approximately threefold higher at 37°C than at 21°C on a per-cell basis, and that MaoP protein levels increase over the first hour following a 21°C-to-37°C temperature shift, whereas MaoP levels remain stable in bacteria maintained at 21°C. These data support temperature-dependent quantitative modulation of the module. Importantly, MaoP-dependent outputs remain condition-dependent, with distinct phenotypes observed under flea-like versus mammalian host-like conditions. We have also revised the discussion to clarify that HdfR coordinates the module by fine-tuning MaoP abundance, and that condition-specific outputs are consistent with a model in which HdfR-mediated changes in MaoP abundance act together with temperature-dependent physiological states.

Now it reads

In the result section

- **Lines 120-125:** Consistent with the condition-dependent phenotypes controlled by the HdfR-MaoP module, *hdfR* expression was higher at 37°C than at 21°C (Extended Data Fig. 5e), estimated MaoP abundance was approximately threefold higher at 37°C than at 21°C on a per-cell basis, and shifting bacteria from 21°C to 37°C increased MaoP protein abundance over the first hour, whereas MaoP levels remained stable in bacteria maintained at 21°C (Extended Data Fig. 5f,g).

In the discussion

- **Lines 340-357:** The difference between MaoP's broad DNA-binding capacity *in vitro* and its promoter occupancy *in vivo* helps reconcile its sequence-agnostic DNA binding *in vitro* with its discrete promoter occupancy *in vivo*. Controlled *maoP* expression in the Δ*hdfR* background restores all tested phenotypes, indicating that HdfR is not required as a promoter-specific cofactor for MaoP activity. Instead, HdfR appears to coordinate the module by tuning MaoP abundance, thereby linking MaoP's broad DNA-binding capacity to vector- and host-aligned molecular programs. Consistent with this model, MaoP binds DNA *in vitro* without detectable sequence specificity, its ChIP-seq peaks lack a clear consensus motif, and bacterial two-hybrid assays detected no interaction with the RNA polymerase subunits or sigma factors tested. These findings argue against strict sequence-specific recognition or protein-mediated recruitment as the main determinants of MaoP occupancy. Rather, MaoP occupancy at discrete promoters likely emerges from broad DNA binding constrained by local chromosomal accessibility, topology, and physiological state, as described for NAPs such as H-NS [34,36]. In this view, MaoP illustrates how architectural DNA-binding proteins can be integrated into environmentally responsive regulatory circuits to coordinate adaptive outputs across changing host-associated niches. In this context, HdfR-mediated tuning of MaoP abundance may ensure that MaoP-dependent phenotypes are expressed under appropriate temperature conditions. Autoregulatory loops at both the HdfR and MaoP levels may further reinforce this robustness.

Line 284: Not clear what is implied by "core functional logic".

We agree that the term "core functional logic" was not sufficiently defined. We have therefore replaced this expression with a more explicit description of the conserved regulatory architecture, referring to the HdfR-dependent control of *maoP* expression, MaoP function as the downstream effector, and the functional interchangeability of HdfR and MaoP homologs for the phenotypes tested. We have revised the manuscript accordingly to avoid ambiguity and overgeneralization.

Now it reads

- **Lines 380-392:** Our data connect these observations by showing that the organization and regulatory hierarchy of HdfR-MaoP have been retained despite substantial sequence divergence. The functional conservation of this scaffold is supported by prior evidence that MaoP expression is HdfR-dependent in

E. coli[45], and by our cross-species complementation experiments showing that HdfR and MaoP from *E. coli* restore all tested phenotypes in the corresponding *Y. pestis* mutants. Conversely, HdfR and MaoP from *Y. pestis* restore biofilm formation in the corresponding *E. coli* mutants, despite the absence of the HdfR-MaoP-regulated *Y. pestis hmsHFRS* biofilm genes in *E. coli*. Together, these findings suggest that HdfR-MaoP is an ancient regulatory module whose outputs have diversified across ecological contexts, with downstream outputs shaped by species-specific physiology and niche-associated pressures. In *Y. pestis*, this scaffold appears to have been integrated into regulatory programs that coordinate flea-borne transmission and mammalian host adaptation.

Line 285: The HdfR-dependence of MaoP expression appears to be important foundational work for this study but is only cursorily mentioned here.

We agree that HdfR-dependent regulation of *maoP* is a foundational aspect of the module. We have revised the text to more clearly emphasize this regulatory relationship and its central role in defining the HdfR-MaoP module, rather than presenting it as a secondary observation.

We agree that HdfR-dependent regulation of maoP is a foundational aspect of the module. We have revised the Discussion to emphasize that the reported HdfR dependence of MaoP expression in *E. coli* supports conservation of the hierarchical HdfR-to-MaoP relationship, rather than presenting it as a secondary observation.

Now it reads

- **Lines 382-385:** The functional conservation of this scaffold is supported by prior evidence that MaoP expression is HdfR-dependent in *E. coli*[45], and by our cross-species complementation experiments showing that HdfR and MaoP from *E. coli* restore all tested phenotypes in the corresponding *Y. pestis* mutants.

Line 289: What data supports role of this module in other bacteria?

We agree that the original phrasing did not sufficiently support the proposed role of this module across bacterial species. We have revised this section to clearly distinguish between conservation of the *hdfR-maoP* locus, previously reported associations in other bacteria, and our own experimental evidence. Importantly, we now explicitly present cross-species complementation data showing that HdfR and MaoP homologs from *E. coli* and *Y. pestis* are functionally interchangeable for the shared biofilm-associated output tested here. Because the *Y. pestis* biofilm genes regulated by HdfR-MaoP are absent from *E. coli*, this conserved biofilm phenotype likely reflects deployment of the module through distinct downstream targets in each species. Based on these combined observations, we have reformulated our interpretation to focus on conservation of the regulatory architecture, while avoiding overgeneralization regarding its functional outputs across species.

Now it reads

- **Lines 382-390:** The functional conservation of this scaffold is supported by prior evidence that MaoP expression is HdfR-dependent in *E. coli*[45], and by our cross-species complementation experiments showing that HdfR and MaoP from *E. coli* restore all tested phenotypes in the corresponding *Y. pestis* mutants. Conversely, HdfR and MaoP from *Y. pestis* restore biofilm formation in the corresponding *E. coli* mutants, despite the absence of the HdfR-MaoP-regulated *Y. pestis hmsHFRS* biofilm genes in *E. coli*. Together, these findings suggest that HdfR-MaoP is an ancient regulatory module whose outputs have diversified across ecological contexts, with downstream outputs shaped by species-specific physiology and niche-associated pressures.

Figure 2c-d: At what temperature are these experiments performed?

These experiments were performed at 21°C, corresponding to flea-relevant conditions and consistent with the analysis of HdfR-MaoP-dependent regulation of the biofilm machinery. This information is now explicitly indicated in the revised figure legend.

Now it reads in the Figure legend:

- **Lines 555-560:** Mean ± SD production of HmsH in Δ*hdfR*….strain ….and grown at 21°C in the absence or presence of arabinose (25 mM)

Figure 2f: Data points on the X-axis for Wt, hdfR mutant, and complement at 27 days post infection.

At 27 days post-infection, a fraction of fleas were no longer infected, resulting in very low or undetectable CFU counts. These points therefore reflect biologically relevant variability in infection status rather than technical artefacts. We have clarified in the revised figure legend that values on the x-axis correspond to fleas with undetectable bacterial loads, plotted at the detection limit for visualization.

Now it reads in the legend of the Figure 2:

- **Lines 566-567:** Data points on the x-axis correspond to fleas with bacterial loads below the detection limit and were plotted at the detection limit for visualization.

Figure 3: Panel d is awkwardly placed in vertical arrangement.

We have updated Figure 3 with new data and adjusted the layout to ensure panels are better integrated.

Line 581: Could not find how differential CFU counts were used in these assays.

This point has now been clarified. Differential CFU counts were obtained by plating flea homogenates on non-selective agar to determine total bacterial loads and on antibiotic-selective agar to enumerate the marked strain. The abundance of the competing strain was then inferred by subtracting selective CFU counts from total CFU counts, and relative abundance was calculated as the proportion of each strain within the total bacterial population recovered from the same flea. This has been clarified in the revised Methods section.

Now it reads

- **Lines 710-715:** ....homogenates were plated on non-selective supplemented LB agar to determine total bacterial loads and on selective supplemented LB agar to enumerate the antibiotic-resistant strain. The abundance of the competing strain was inferred by subtracting selective CFU counts from total CFU counts, and bacterial competition was quantified as the relative proportion of each strain within the total bacterial population recovered from each individual flea.

Line 710: Reference 48 is now published.

Reference 48 (now 58) has been updated to cite the published version.

Reviewer #2 (Remarks to the Author):

This manuscript describes the identification and characterisation of a regulatory hub in Y. pestis involved in controlling the transmission from flea to mammal. The manuscript shows that the HdfR-MaoP hub is required by Y. pestis for normal resistance to mammalian innate immunity effectors such as AMPs and biofilm formation (in the flea gut). As such the authors propose that this minimal regulatory hub "provides a mechanistic framework for overcoming the conflicting demands imposed by alternating vector and mammalian environments".

Comments

line 184: MaoS sites are mentioned but the location of these sites is not indicated anywhere in the manuscript.

We now explicitly indicate that the MaoS site is located in the intergenic region between *hdfR* and *maoP*. In addition, Extended Data Fig. 12 and 13 has been revised to show the position of the MaoS site within the conserved genomic context of the *hdfR-maoP* locus in representative Gammaproteobacteria.

Now I reads:

- **Lines 241-243:** Deletion of the MaoS site, located in the intergenic region between *hdfR* and *maoP*, abolished endogenous *maoP* transcription and….

line 199: MaoP apparently binds DNA in a sequence-agnostic manner but, nonetheless, in discrete promoter regions. The authors argue that this specificity may be due to local chromosomal topology. Does HdfR (a bona fide transcriptional regulator) have a recognition sequence that perhaps imparts specificity to a HdfR-MaoP complex?? Are there HdfR-regulated genes that are dependent and independent of MaoP??

We agree that the apparent discreteness of MaoP occupancy in vivo, despite sequence-independent DNA binding in vitro, required clarification. We have revised the manuscript to state more explicitly that MaoP binds DNA without detectable sequence specificity under the conditions tested. We also now conclude this section by indicating that MaoP in vivo occupancy is likely shaped by local chromosomal context rather than by strict sequence-specific recruitment.

Now it reads:

- **Lines 252-257:** ChIP-seq revealed MaoP occupancy at discrete promoter regions, including its own locus, the zinc uptake regulator *zur*, the biofilm operon *hmsHFRS* and the pMT1-borne caf1R-caf1M1A1 region (Supplementary Table 2). However, microscale thermophoresis measurements demonstrated that MaoP binds DNA without detectable sequence specificity under the conditions tested (Fig. 4b).
- **Lines 259-262:** Together, these findings support the designation of MaoP as a nucleoid-associated protein involved in modulating local DNA conformation and transcriptional outputs, with *in vivo* occupancy likely shaped by local chromosomal context rather than strict sequence-specific recruitment.

Regarding the possibility that HdfR confers promoter specificity to MaoP through an HdfR-MaoP recruitment complex, our data do not require such a model for the phenotypes tested here. Controlled expression of *maoP* in the Δ*hdfR* background restores all tested phenotypes in a dose-dependent manner, indicating that MaoP is sufficient to drive these outputs once expressed. We have therefore revised the discussion to clarify that HdfR primarily tunes MaoP abundance in this module, rather than being required as an obligatory promoter-specific cofactor for MaoP activity.

Now it reads:

- **Lines 342-352:** Controlled *maoP* expression in the Δ*hdfR* background restores all tested phenotypes, indicating that HdfR is not required as a promoter-specific cofactor for MaoP activity. Instead, HdfR appears to coordinate the module by tuning MaoP abundance, thereby linking MaoP's broad DNA-binding capacity to vector- and host-aligned molecular programs. Consistent with this model, MaoP binds DNA *in vitro* without detectable sequence specificity, its ChIP-seq peaks lack a clear consensus motif, and bacterial two-hybrid assays detected no interaction with the RNA polymerase subunits or sigma factors tested. These findings argue against strict sequence-specific recognition or protein-mediated recruitment as the main determinants of MaoP occupancy. Rather, MaoP occupancy at discrete promoters likely emerges from broad DNA binding constrained by local chromosomal accessibility, topology, and physiological state, as described for NAPs such as H-NS [34,36].

We also agree that HdfR may regulate additional targets independently of MaoP in other bacterial contexts. We have therefore revised the Results to avoid implying that MaoP is the only possible HdfR-dependent output. Specifically, we now state that inspection of the proteomic dataset suggested that HdfR primarily acts through activation of MaoP, and we reserve the stronger conclusion for the subsequent genetic evidence, stating that MaoP is the indispensable downstream effector of the HdfR regulon for the phenotypes tested here. We have also added discussion of studies showing direct HdfR binding to promoters controlling adaptive traits in other *Enterobacterales*, including *flhDC* in *E. coli* and the *std* fimbrial operon in *Salmonella enterica*. These examples clarify that HdfR-centered regulatory circuits can be wired to species-specific physiological programs, while our genetic data indicate that MaoP accounts for the major HdfR-dependent outputs tested here in Y. pestis.

Now it reads

- **Lines 107-109:** Despite this broad impact, inspection of the dataset suggested that HdfR primarily acts through the activation of a single downstream factor, MaoP.
- **Lines 342-344:** Controlled *maoP* expression in the Δ*hdfR* background restores all tested phenotypes, indicating that HdfR is not required as a promoter-specific cofactor for MaoP activity.
- **Lines 368-370:** In *S. enterica*, HdfR directly regulates the horizontally acquired *std* fimbrial operon[37], showing that conserved regulators can be wired to species-specific virulence programs.
- **Lines 378-380:** Consistent with this functional diversification, HdfR has been shown to bind directly to promoters controlling adaptive traits in other *Enterobacterales*, including the flagellar master regulator *flhDC* in *E. coli* and the *std* fimbrial operon in *S. enterica*[37,44].

Figure 3b: the DOC-PAGE presented is not of great quality. Do all LOS bands have the same mobility?

We acknowledge that the DOC-PAGE image in the initial version was not optimally presented, as the gel image was slightly tilted. We have corrected the image alignment to improve readability. The major LOS bands display comparable electrophoretic mobility across strains under the conditions tested, with no obvious shift in migration or gross alteration in banding pattern. These data indicate that the observed phenotype reflects reduced LOS abundance rather than a detectable change in the migration pattern of the major LOS species.

Reviewer #3 (Remarks to the Author):

This work led by Baillez et. al. identifies the HdfR-MaoP module as a putative regulatory hub governing host transition in Yersinia pestis. The authors argue that HdfR-dependent activation of MaoP coordinates flea-associated biofilm formation with resistance to mammalian host immunity, and

further propose that this regulatory logic is conserved across enteric bacteria. The study combines extensive multi-omics data, biochemical analyses, and infection models to investigate this pathway, and the broader evolutionary framework is conceptually appealing. However, several key mechanistic gaps remain unresolved, and addressing these issues would substantially strengthen the manuscript.

1. The major weakness of the work is the lack of mechanistic gaps and discrepancies between assays, and some areas requiring further clarification. My major concern is the function and mechanism of the nucleoid-associated protein-like factor MaoP, which sits in the central part in the proposed regulatory switch. The authors demonstrate via microscale thermophoresis that the MaoP binds DNA in a sequence-agnostic manner in Fig4b. However, ChIP-seq reveals MaoP occupancy at discrete promoter regions (such as its own locus, zur, and hmsHFRS). It is not sufficiently explained how a sequence-agnostic DNA-binding protein achieves this highly specific transcriptional targeting. The manuscript would benefit from a proposed model or additional experiments explaining how MaoP is recruited to specific promoters (e.g., through protein-protein interactions with other specific transcription factors, despite the negative bacterial two-hybrid results with RNA polymerase).

We agree that reconciling the apparent discrepancy between sequence-agnostic DNA binding *in vitro* and selective promoter occupancy *in vivo* is an important point. Our data indicate that MaoP does not rely on intrinsic sequence-specific DNA recognition. In addition, controlled expression of *maoP* in a Δ*hdfR* background restores all tested phenotypes, indicating that MaoP activity does not require HdfR to act as an obligatory promoter-specific recruitment factor once MaoP is expressed. We therefore favor a model in which the HdfR-MaoP module operates as a quantitative regulatory system, with HdfR tuning MaoP abundance in response to environmental cues such as temperature. MaoP, in turn, functions as a nucleoid-associated protein that lacks detectable sequence specificity under the conditions tested but modulates local DNA conformation. As a result, the discrete MaoP occupancy observed at promoters such as *maoP*, *zur* and *hmsHFRS* is unlikely to be driven by strict sequence-specific recognition and likely reflects context-dependent chromosomal features, including local DNA accessibility, topology or chromosomal neighborhood, although the precise determinants remain to be defined. This interpretation is consistent with the absence of detectable sequence specificity in vitro, the ability of MaoP to alter DNA conformation, and the lack of a clear consensus motif among ChIP-seq peaks. While we cannot exclude that MaoP may interact with additional factors, our data do not support an obligatory requirement for a specific protein-protein interaction, including with HdfR or RNA polymerase, to explain the phenotypes examined here. The manuscript has been revised to clarify this model and its current limitations.

Now it reads

- **Lines 332-336:** Together, these biochemical and functional properties place MaoP within the NAP functional spectrum. However, rather than acting as an autonomous global nucleoid organizer, MaoP appears to function as a quantitatively tuned architectural effector embedded in an HdfR-controlled, temperature-responsive regulatory module.
- **Lines 340-357:** The difference between MaoP's broad DNA-binding capacity *in vitro* and its promoter occupancy *in vivo* helps reconcile its sequence-agnostic DNA binding *in vitro* with its discrete promoter occupancy *in vivo*. Controlled *maoP* expression in the Δ*hdfR* background restores all tested phenotypes, indicating that HdfR is not required as a promoter-specific cofactor for MaoP activity. Instead, HdfR appears to coordinate the module by tuning MaoP abundance, thereby linking MaoP's broad DNA-binding capacity to vector- and host-aligned molecular programs. Consistent with this model, MaoP binds DNA *in vitro* without detectable sequence specificity, its ChIP-seq peaks lack a clear consensus motif, and bacterial two-hybrid assays detected no interaction with the RNA polymerase subunits or sigma factors tested. These findings argue against strict sequence-specific recognition or protein-mediated recruitment as the main determinants of MaoP occupancy. Rather, MaoP occupancy at discrete promoters likely emerges from broad DNA binding constrained by local chromosomal

accessibility, topology, and physiological state, as described for NAPs such as H-NS [34,36]. In this view, MaoP illustrates how architectural DNA-binding proteins can be integrated into environmentally responsive regulatory circuits to coordinate adaptive outputs across changing host-associated niches. In this context, HdfR-mediated tuning of MaoP abundance may ensure that MaoP-dependent phenotypes are expressed under appropriate temperature conditions. Autoregulatory loops at both the HdfR and MaoP levels may further reinforce this robustness.

2. There is a notable discrepancy regarding MaoP's effect on DNA topology. In vitro atomic force microscopy (AFM) visualizes local changes in DNA conformation upon MaoP binding. Conversely, in vivo Hi-C reveals no major changes in global or local chromosome folding. While the authors speculate this may be due to highly dynamic or heterogeneous MaoP-DNA interactions, they should discuss whether the resolution of their Hi-C mapping (1kb bins) is sufficient to detect the fine, local topological bends observed via AFM, or if other in vivo assays could bridge this gap.

We agree that reconciling the local DNA conformational changes observed by AFM with the absence of major effects in Hi-C maps requires clarification. These observations are not contradictory but reflect differences in scale, resolution, and averaging between the two approaches. AFM provides a high-resolution view of local conformational changes on individual DNA molecules, whereas Hi-C captures population-averaged chromosomal contact frequencies at kilobase resolution in our dataset. Under these conditions, subtle, transient, heterogeneous, or highly localized DNA bending events are unlikely to be detected by Hi-C. This is consistent with a model in which MaoP acts as a locally acting architectural factor that can alter DNA conformation without inducing detectable local or global chromosome reorganization at the resolution tested (1kb bin).

We agree that higher-resolution chromosome conformation approaches, such as Micro-C, may in principle provide additional insight into such local structural effects. However, their application in this context remains technically challenging and beyond the scope of the present study. We have revised the discussion to clarify this point.

Now it reads

- **Lines 322-329:** It binds DNA without detectable sequence specificity and alters DNA conformation in vitro, as shown by MST and AFM. By contrast, Hi-C contact maps and contact-probability decay analyses did not reveal major changes in chromosome folding *in vivo*. This difference likely reflects the different scales probed by the two approaches, with AFM detecting local single-molecule DNA conformational changes, whereas Hi-C reports population-averaged chromosomal contacts at kilobase resolution, as well as potentially dynamic or heterogeneous MaoP-DNA interactions, as previously reported for H-NS[33]. Thus, transient, heterogeneous, or sub-kilobase MaoP-DNA interactions may not produce detectable changes in global Hi-C profiles.

3. The proteomic data indicate that MaoP is a relatively abundant DNA-binding protein, yet the manuscript does not discuss this abundance relative to established Yersinia nucleoid-associated factors such as H-NS, YmoA/Hha, IscR. Such comparison would be valuable, particularly because it remains unclear whether MaoP performs overlapping, complementary, or distinct regulatory functions. At minimum, the authors should discuss whether these small DNA-binding proteins are deployed in different environmental niches or act cooperatively to fine-tune virulence-associated programs during the complex life cycle of Y. pestis.

We agree that MaoP abundance should be documented more explicitly. We have therefore added an estimate of MaoP copy number per bacterium and described the quantification method in the revised manuscript. These data show that MaoP is present at high cellular abundance (>10^4 molecules per cell at 37°C), consistent with a nucleoid-associated function rather than that of a classical low-abundance transcription factor. We have not expanded the discussion into a comparison with other Yersinia DNA-

binding regulators because direct cooperation between MaoP and factors such as H-NS, YmoA/Hha-like proteins or IscR was not tested in this study. We therefore avoid speculating on cooperative deployment and instead frame MaoP as a nucleoid-associated component of the HdfR-MaoP module whose interactions with other chromosomal regulators remain to be defined.

Now it reads

In the method section

- **Lines 930-943: Determination of MaoP copy number per bacteria.** MaoP copy number per bacterium was estimated by quantitative immunoblotting. Clarified lysates were incubated with anti-FLAG magnetic beads (GenScript) for 2 h at 4°C with gentle rotation. Beads were washed four times with Wash buffer and MaoP-FLAG was eluted using the Alkaline elution buffer. After neutralization as recommended by the manufacturer, the purified proteins were quantified by BCA assay. Purified MaoP-FLAG was used as a calibration standard and loaded alongside whole-cell extracts prepared from 2.5 x 10^8 *Y. pestis* grown at 21°C or 37°C. Samples were resolved by electrophoresis, transferred, and probed with an anti-FLAG antibody. Band intensities were quantified and compared with the purified MaoP-FLAG standard curve to determine the amount of MaoP-FLAG in each extract. Molecule numbers were derived using the predicted molecular weight of MaoP-FLAG (14,065 kDa) and Avogadro's constant, then normalized to bacterial cell counts. Details on magnetic beads and Antibody Supplementary Table 5 as well as buffer compositions are provided in Supplementary Table 6.

In the result section

- **Lines 120-125:** Consistent with the condition-dependent phenotypes controlled by the HdfR-MaoP module, *hdfR* expression was higher at 37°C than at 21°C (Extended Data Fig. 5e), estimated MaoP abundance was approximately threefold higher at 37°C than at 21°C on a per-cell basis, and shifting bacteria from 21°C to 37°C increased MaoP protein abundance over the first hour, whereas MaoP levels remained stable in bacteria maintained at 21°C (Extended Data Fig. 5f,g).
- **Lines 250-252:** MaoP is a small (14-kDa), non-highly basic DNA-binding protein, with an estimated abundance of approximately 1.4 x 10^4 and 4.4 x 10^4 molecules per cell at 21°C and 37°C respectively (Extended Data Fig. 5f).

In the discussion

- **Lines 336-339:** How MaoP functionally interfaces with established nucleoid-associated proteins and DNA-binding regulators remains to be defined. These factors may act together with MaoP to modulate virulence-associated gene expression under specific conditions.

4. The manuscript focuses primarily on chromosomal regulatory effects, but does not address whether MaoP-dependent DNA binding and topological changes could also extend to the virulence plasmids of Y. pestis. This is an important omission, given that the type III secretion system is encoded on pCD1 and that additional plasmids, including pMT1 and pPCP1/pPla, are central to pathogenicity and flea-mammal life circle. The authors should discuss whether MaoP might affect plasmid topology and thereby influence plasmid-encoded virulence functions.

We have now extended our ChIP-seq analysis to the plasmids present in the strain used in this study, namely pMT1 and pPCP1/pPla. This analysis identified MaoP occupancy on pMT1, including in the vicinity of the *caf* locus, whereas no MaoP-associated enrichment was detected on pPCP1/pPla (Supplementary Table 2). Western-blot analysis further showed that deletion of *maoP* did not affect Pla production, whereas Caf1 production was increased under the conditions tested. We also clarify that the strain used in this study does not carry the pCD1 plasmid, and therefore the potential impact of MaoP on type III secretion system regulation cannot be assessed in this background. This information has been included in the revised manuscript.

Now it reads

In the method section

- **Lines 859-860:** *Y. pestis* lacking pCD1 and *pla*, while expressing a…

In the result section

- **Lines 217-231: Plasmid-borne virulence-associated genes are integrated into the HdfR-MaoP regulatory network.** Proteomic analyses of the Δ*hdfR* mutant revealed increased abundance of Caf1R and Caf1 (Fig. 1d), both encoded on the pMT1 plasmid. Caf1R activates the *caf1M1A1* operon at mammalian host temperature, leading to production of the F1 capsule, a major *Y. pestis* virulence factor [28,29]. Consistent with the hierarchical organization of the HdfR-MaoP module, deletion of *maoP* increased Caf1 production under the conditions tested, suggesting that HdfR's effect on Caf1 abundance is mediated, at least in part, by MaoP (Extended Data Fig. 8a). This effect did not extend to all plasmid-encoded virulence factors, as production of the pPla-encoded virulence factor Pla was unchanged in the Δ*maoP* mutant (Extended Data Fig. 8b). The potential impact of HdfR-MaoP on the virulence pCD1-encoded functions could not be assessed because the strain used in this study lacks this plasmid. Altogether, these data indicate that HdfR-MaoP regulation can extend to specific plasmid-borne functions, such as Caf1 production on pMT1, suggesting that horizontally acquired elements can be integrated into host-associated regulatory networks governed by this module.

In the Discussion

- **Lines 367-372:** The HdfR-MaoP module also illustrates how conserved regulators can incorporate horizontally acquired virulence genes…. In *Y. pestis*, HdfR-MaoP-dependent regulation similarly extends beyond chromosomal loci to selected plasmid-borne virulence-associated genes, including the pMT1-encoded *caf* locus.

In the Figure legend

Lines 1240-1242: Extended Data Figure 8. MaoP affects Caf1 production but not Pla production. (A-B) Relative amounts of Caf1 (A) and Pla forms (B) produced in the Δ*maoP* and complemented (-c) strains compared with WT, measured by immunoblot (n = 4).

5. The conclusion that MaoP regulates hmsHFRS through parallel Zur-dependent and Zur-independent pathways raises an important mechanistic question that is left unresolved. Because the Δzur mutant displays a less severe phenotype than ΔmaoP, while the double mutant shows the strongest defect, the authors infer the existence of an additional Zur-independent route connecting MaoP to biofilm regulation. However, no hypothesis is offered. In addition, the function of Zur itself deserves more careful consideration. I wonder if Zur is simply a zinc uptake regulator, the authors should discuss the environmental contexts or host niches in which Zur-dependent control would be expected to operate during the Y. pestis life cycle. It would also be useful to clarify whether Zur acts solely in zinc homeostasis here or may intersect more broadly with other metal-responsive regulatory networks, particularly those related to iron, which could be more physiologically relevant in infection settings.

We acknowledge that we did not provide a mechanistic explanation for the Zur-independent contribution to MaoP-mediated regulation of biofilm formation. To address this point, we explored the possibility of post-transcriptional regulation through small RNAs previously implicated in the control of the *hmsHFRS* operon and c-di-GMP production via *hmsT*. However, these analyses did not support this hypothesis, as c-di-GMP levels were unchanged in the Δ*hdfR* mutant and the tested sRNA genes were not reduced in the Δ*maoP* mutant. These results indicate that the Zur-independent component does not appear to involve the tested sRNA-dependent pathways or global changes in c-

di-GMP levels, suggesting the existence of additional regulatory mechanisms. We have provided a new figure and revised the manuscript to explicitly acknowledge this point as an open question.

We also agree that Zur's potential role beyond canonical zinc homeostasis, including possible intersections with other metal-responsive networks, is an important consideration. However, our current data do not directly address these interactions. We have therefore limited our interpretation to the experimentally supported Zur-dependent regulation of *hmsHFRS* and to the new analyses excluding the tested sRNA-dependent and global c-di-GMP pathways. We now explicitly state in the Results that the mechanism underlying the Zur-independent branch remains to be defined.

Now it reads:

In the method section

- **Lines 920-928: c-di-GMP quantification.** c-di-GMP was extracted and quantified by HPLC as described previously [64], with minor modifications. Briefly, bacterial cultures were fixed with 0.18% formaldehyde, pelleted, washed, and resuspended in distilled water. An aliquot was used for protein normalization by BCA assay, while the remaining sample was heated at 99°C and extracted sequentially with 95% and 70% ethanol. Dried extracts were resuspended in nanopure water and analyzed on a Dionex Chromeleon HPLC system equipped with a reverse-phase C18 column. c-di-GMP was eluted using ammonium acetate-based water/methanol solvents, detected at 253 nm, quantified using a commercial c-di-GMP standard curve, and normalized to total protein content. Details on buffer compositions are provided in Supplementary Table 6.

In the result section

- **Lines 161-166:** Small RNAs have been reported to activate translation of *hmsHFRS* and *hmsT*, which encodes a diguanylate cyclase that produces c-di-GMP required for HmsHFRS activity, providing a plausible molecular basis for the Zur-independent branch[23-25]. However, c-di-GMP levels were unchanged in the Δ*hdfR* mutant, and deletion of *maoP* did not reduce expression of the tested sRNA genes (Extended Data Fig. 6). The mechanism underlying the Zur-independent regulation of *hmsHFRS* therefore remains to be defined.

In the Figure Legend section

- **Lines 1211-1217: Extended Data Figure 6. The HdfR-MaoP module does not affect sRNA- or c-di-GMP-dependent regulation of HmsHFRS in *Y. pestis* microtus.** (**A**) Mean ± SD promoter activity of genes encoding sRNAs previously reported to activate *hmsHFRS* and *hmsT* translation, measured in WT and Δ*maoP* strains cultured at 21°C and carrying transcriptional GFP reporters. Data are expressed as relative fluorescence units (RFU) relative to WT (n = 3). (**B**) Mean ± SD c-di-GMP levels in WT, Δ*hdfR* and complemented (-c) strains cultured at 21°C (n = 2).

6. The authors provide convincing evidence that MaoP limits complement activation by restricting the binding of mannose-binding lectin (MBL) and C1q. They conclude that the HdfR-MaoP module "masks surface ligands across temperatures to restrict complement activation". However, the actual identity of these immunogenic surface determinants remains unknown. Is it possible to identify which specific outer membrane components or surface ligands are being masked to prevent classical and lectin pathway activation?

Prior to submission, we evaluated a panel of 27 candidate genes selected from our omics datasets. None of the tested mutants individually recapitulated the complement sensitivity phenotype, indicating that no single candidate determinant tested was sufficient to explain the observed effect. These results support a model in which the HdfR-MaoP module influences complement resistance through distributed changes in surface ligand exposure or accessibility rather than through masking of one dominant determinant. Consistently, the Δ*maoP* mutant shows increased binding of both MBL

and C1q, supporting broader alteration of surface recognition by the lectin and classical pathways. The corresponding data are now provided in Extended Data Figure 7e, and the manuscript has been revised to reflect this distributed mode of action.

Now it reads:

- **Lines 203-208:** To identify potential determinants of this increased recognition, we screened 27 candidate surface-associated genes selected from our omics datasets. None of the corresponding mutants individually recapitulated the complement sensitivity phenotype, supporting a distributed effect on surface ligand exposure rather than masking of one dominant determinant (Extended Data Fig. 7e).

Now it reads in the Figure legend

- **Lines 1234-1238:** (**E**) Mean ± SD survival of WT, Δ*hdfR*, and mutants lacking genes identified as putatively regulated by the HdfR-MaoP module in the present omics analysis, after exposure to normal human serum. Survival was quantified as Log_{10} RLU (N/N_0) using bacteria grown at 21°C and then exposed to serum at 37°C for 180 min, where N corresponds to luminescence after exposure and N_0 to luminescence before exposure.

7. In Fig 3, the authors links AMP susceptibility at 37°C to impaired lipooligosaccharide (LOS) core maturation, specifically through reduced expression of gmhA and rfaD. However, ectopic expression of either gmhA or rfaD in the ΔmaoP mutant only partially restored polymyxin resistance. This partial rescue strongly suggests that other unknown factors within the MaoP regulon are required for full AMP resistance. The authors should address this and discuss what other HdfR-MaoP-dependent envelope modifications might be contributing to this phenotype.

We agree that the partial restoration of polymyxin resistance upon expression of either *gmhA* or *rfaD* alone required clarification. We have now added co-expression data showing that combined expression of *gmhA* and *rfaD* further restores polymyxin resistance, most notably at higher polymyxin concentrations. These results strengthen the conclusion that impaired LOS core maturation is a major determinant of the antimicrobial peptide susceptibility of the Δ*maoP* mutant and indicate that coordinated activity of both enzymes is required for efficient restoration.

We also provide new data indicating that this phenotype cannot be explained by reduced expression of canonical lipid A modification pathways associated with polymyxin resistance. In particular, transcription of the *pmrF* operon and upstream genes involved in aminoarabinose modification was not reduced in the Δ*maoP* mutant, whereas the phosphoethanolamine transferase gene *eptA* was increased. Thus, HdfR-MaoP-dependent peptide resistance primarily involves LOS core maturation through coordinated GmhA and RfaD output, rather than reduced activation of canonical lipid A modification pathways. We have added these data and revised the manuscript accordingly.

Now it reads

- **Lines 181-191:** Consistent with the coordinated contribution of both enzymes to LOS core biosynthesis, combined expression of *gmhA* and *rfaD* further restored polymyxin resistance, most notably at higher polymyxin concentrations (Fig. 3f). Inspection of the remaining LOS and lipid A biosynthesis genes revealed no reduced expression in Δ*maoP* (Extended Data Fig. 7b,c). In addition, canonical lipid A modification pathways associated with polymyxin resistance were not transcriptionally reduced, including the *pmrF* operon and upstream genes involved in aminoarabinose modification, whereas *eptA* was increased (Extended Data Fig. 7b). These data support the conclusion that peptide sensitivity stems from impaired LOS core maturation through reduced GmhA and RfaD output rather than from a broader disruption of LPS assembly or reduced expression of canonical lipid A modification pathways.

Now it reads in the Figure Legends:

- **Lines 598-600:** (D-F) Mean ± SD survival of WT and Δ*maoP* strains expressing or not *gmhA* (D), *rfaD* (E) or both *gmhA* and *rfaD* (F) from an arabinose-inducible promoter (pBAD30'-*gmhA*; p_{gmhA}' or pBAD30-*rfaD*; p_{rfaD})

8. The authors identify the FDA-approved drug flunixin as a chemical inhibitor that reduces maoP promoter activity in an HdfR-dependent manner, thereby sensitizing Y. pestis to AMPs. While an exciting finding, the molecular mechanism is entirely unexplored. It is unclear if flunixin directly binds to the HdfR protein to inhibit its transcriptional activation function, or if it acts upstream. Providing biochemical evidence of direct binding (e.g., using the purified Y. pestis HdfR in microscale thermophoresis or surface plasmon resonance assays) would substantially elevate the pharmacological relevance of this finding.

We agree that biochemical evidence of a direct interaction between flunixin and HdfR would provide additional mechanistic insight. We now demonstrate that flunixin directly interacts with HdfR, as shown by microscale thermophoresis using purified protein, with a dissociation constant (Kd) of approximately 1 µM. These data provide direct biochemical support for an HdfR-dependent mode of action. We have incorporated these results into the revised manuscript and adjusted the interpretation accordingly.

Now it reads:

- **Lines 297-299:** Furthermore, at the biochemical level, microscale thermophoresis using purified HdfR showed direct binding of flunixin to HdfR, with an apparent Kd of approximately 1 µM (Fig. 5f).

Now it reads in the Figure Legends:

- **Lines 639-642:** (**F**) Flunixin binding assessed by MST using purified HdfR. Fraction bound is plotted as a function of flunixin concentration. Data points represent mean ± SD from three independent measurements, and solid lines indicate fits to a single-site binding model.

9. The full complementation of the mutant phenotype by E. coli HdfR and MaoP is particularly striking. In fact, this result appears to challenge, rather than support, the authors' broader hypothesis that the HdfR-MaoP regulatory hub evolved specifically in Y. pestis to coordinate adaptation between arthropod and mammalian hosts, given that E. coli does not share this dual-host lifestyle. What is the physiological function of the HdfR-MaoP system in E. coli and in other Enterobacteriaceae? A clearer discussion of its ancestral role would help determine whether Y. pestis has uniquely adapted a conserved regulatory circuit for vector-host transition, or whether the manuscript is overstating the degree of lifestyle-specific specialization.

We agree that the functional complementation observed with *E. coli* HdfR and MaoP raises important questions regarding the evolutionary origin and conservation of this regulatory module. Our revised data show that HdfR and MaoP from *E. coli* fully complement the corresponding *Y. pestis* mutants, and we further demonstrate in the revised version that deletion of *hdfR* or *maoP* in *E. coli* impairs biofilm formation, a phenotype restored by expression of the corresponding *Y. pestis* genes. These results indicate that key functional properties of the module are conserved across species and that the HdfR-MaoP system is not unique to the flea-mammal lifestyle of *Y. pestis*. Importantly, however, the downstream regulatory outputs differ between species. In *Y. pestis*, the module controls biofilm formation through regulation of the *hmsHFRS* operon, which is absent from *E. coli*, indicating that similar regulatory components can act through distinct genetic targets.

We have therefore revised the manuscript to clarify that HdfR-MaoP represents an ancestral regulatory scaffold that has been rewired during evolution to control different targets and

physiological outputs depending on species-specific physiology and ecological context. In *Y. pestis*, this conserved module has been integrated into the flea-mammal transmission cycle, where it coordinates biofilm-mediated transmission with mammalian-stage immune resistance.

Now it reads:

In the result section

- **Lines 276-282:** Conversely, deletion of *hdfR* or *maoP* in *E. coli* impaired biofilm formation, and expression of the corresponding *Y. pestis* homolog restored this defect, indicating reciprocal functional conservation for this shared biofilm-associated output (Extended Data Fig. S12i). These findings indicate that the HdfR-MaoP module constitutes an evolutionarily conserved regulatory unit, with conserved genomic organization, functional interchangeability, and shared biofilm-associated regulatory activity, while immune-resistance outputs may reflect species- and niche-specific deployment of this regulatory scaffold.

In the result discussion

- **Lines 380-392:** Our data connect these observations by showing that the organization and regulatory hierarchy of HdfR-MaoP have been retained despite substantial sequence divergence. The functional conservation of this scaffold is supported by prior evidence that MaoP expression is HdfR-dependent in *E. coli*[45], and by our cross-species complementation experiments showing that HdfR and MaoP from *E. coli* restore all tested phenotypes in the corresponding *Y. pestis* mutants. Conversely, HdfR and MaoP from *Y. pestis* restore biofilm formation in the corresponding *E. coli* mutants, despite the absence of the HdfR-MaoP-regulated *Y. pestis hmsHFRS* biofilm genes in *E. coli*. Together, these findings suggest that HdfR-MaoP is an ancient regulatory module whose outputs have diversified across ecological contexts, with downstream outputs shaped by species-specific physiology and niche-associated pressures. In *Y. pestis*, this scaffold appears to have been integrated into regulatory programs that coordinate flea-borne transmission and mammalian host adaptation.

This work led by Baillez et. al. identifies the HdfR-MaoP module as a putative regulatory hub governing host transition in *Yersinia pestis*. The authors argue that HdfR-dependent activation of MaoP coordinates flea-associated biofilm formation with resistance to mammalian host immunity, and further propose that this regulatory logic is conserved across enteric bacteria. The study combines extensive multi-omics data, biochemical analyses, and infection models to investigate this pathway, and the broader evolutionary framework is conceptually appealing. However, several key mechanistic gaps remain unresolved, and addressing these issues would substantially strengthen the manuscript.

1. The major weakness of the work is the lack of mechanistic gaps and discrepancies between assays, and some areas requiring further clarification. My major concern is the function and mechanism of the nucleoid-associated protein-like factor MaoP, which sits in the central part in the proposed regulatory switch. The authors demonstrate via microscale thermophoresis that the MaoP binds DNA in a sequence-agnostic manner in Fig4b. However, ChIP-seq reveals MaoP occupancy at discrete promoter regions (such as its own locus, *zur*, and *hmsHFRS*). It is not sufficiently explained how a sequence-agnostic DNA-binding protein achieves this highly specific transcriptional targeting. The manuscript would benefit from a proposed model or additional experiments explaining how MaoP is recruited to specific promoters (e.g., through protein-protein interactions with other specific transcription factors, despite the negative bacterial two-hybrid results with RNA polymerase).

2. There is a notable discrepancy regarding MaoP's effect on DNA topology. In vitro atomic force microscopy (AFM) visualizes local changes in DNA conformation upon MaoP binding. Conversely, in vivo Hi-C reveals no major changes in global or local chromosome folding. While the authors speculate this may be due to highly dynamic or heterogeneous MaoP-DNA interactions, they should discuss whether the resolution of their Hi-C mapping (1kb bins) is sufficient to detect the fine, local topological bends observed via AFM, or if other in vivo assays could bridge this gap.

3. The proteomic data indicate that MaoP is a relatively abundant DNA-binding protein, yet the manuscript does not discuss this abundance relative to established Yersinia nucleoid-associated factors such as H-NS, YmoA/Hha, IscR. Such comparison would be valuable, particularly because it remains unclear whether MaoP performs overlapping, complementary, or distinct regulatory functions. At minimum, the authors should discuss whether these small DNA-binding proteins are deployed in different environmental niches or act cooperatively to fine-tune virulence-associated programs during the complex life cycle of *Y. pestis*.

4. The manuscript focuses primarily on chromosomal regulatory effects, but does not address whether MaoP-dependent DNA binding and topological changes could also extend to the virulence plasmids of *Y. pestis*. This is an important omission, given that the type III secretion system is encoded on pCD1 and that additional plasmids, including pMT1 and pPCP1/pPla, are central to pathogenicity and flea-mammal life circle. The authors should

discuss whether MaoP might affect plasmid topology and thereby influence plasmid-encoded virulence functions.

5. The conclusion that MaoP regulates hmsHFRS through parallel Zur-dependent and Zur-independent pathways raises an important mechanistic question that is left unresolved. Because the Δ*zur* mutant displays a less severe phenotype than Δ*maoP*, while the double mutant shows the strongest defect, the authors infer the existence of an additional Zur-independent route connecting MaoP to biofilm regulation. However, no hypothesis is offered. In addition, the function of Zur itself deserves more careful consideration. I wonder if Zur is simply a zinc uptake regulator, the authors should discuss the environmental contexts or host niches in which Zur-dependent control would be expected to operate during the *Y. pestis* life cycle. It would also be useful to clarify whether Zur acts solely in zinc homeostasis here or may intersect more broadly with other metal-responsive regulatory networks, particularly those related to iron, which could be more physiologically relevant in infection settings.

6. The authors provide convincing evidence that MaoP limits complement activation by restricting the binding of mannose-binding lectin (MBL) and C1q. They conclude that the HdfR-MaoP module "masks surface ligands across temperatures to restrict complement activation". However, the actual identity of these immunogenic surface determinants remains unknown. Is it possible to identify which specific outer membrane components or surface ligands are being masked to prevent classical and lectin pathway activation?

7. In Fig 3, the authors links AMP susceptibility at 37°C to impaired lipooligosaccharide (LOS) core maturation, specifically through reduced expression of gmhA and rfaD. However, ectopic expression of either *gmhA* or *rfaD* in the Δ*maoP* mutant only partially restored polymyxin resistance. This partial rescue strongly suggests that other unknown factors within the MaoP regulon are required for full AMP resistance. The authors should address this and discuss what other HdfR-MaoP-dependent envelope modifications might be contributing to this phenotype.

8. The authors identify the FDA-approved drug flunixin as a chemical inhibitor that reduces maoP promoter activity in an HdfR-dependent manner, thereby sensitizing *Y. pestis* to AMPs. While an exciting finding, the molecular mechanism is entirely unexplored. It is unclear if flunixin directly binds to the HdfR protein to inhibit its transcriptional activation function, or if it acts upstream. Providing biochemical evidence of direct binding (e.g., using the purified *Y. pestis* HdfR in microscale thermophoresis or surface plasmon resonance assays) would substantially elevate the pharmacological relevance of this finding.

9. The full complementation of the mutant phenotype by *E. coli* HdfR and MaoP is particularly striking. In fact, this result appears to challenge, rather than support, the authors' broader hypothesis that the HdfR-MaoP regulatory hub evolved specifically in Y. *pestis* to coordinate adaptation between arthropod and mammalian hosts, given that E. coli

does not share this dual-host lifestyle. What is the physiological function of the HdfR-MaoP system in *E. coli* and in other Enterobacteriaceae? A clearer discussion of its ancestral role would help determine whether *Y. pestis* has uniquely adapted a conserved regulatory circuit for vector-host transition, or whether the manuscript is overstating the degree of lifestyle-specific specialization.
