# Supplementary material for: A dual-host regulatory hub integrating biofilm formation and innate immunity resistance
Source: Nat Commun. 2026 Sep 1;17:10385. doi: 10.1038/s41467-026-77307-4 (PMC13627719; doi:10.1038/s41467-026-77307-4)
Supplement: Supplementary file 2 [file 41467_2026_77307_MOESM1_ESM.pdf]

Supplementary Figures.

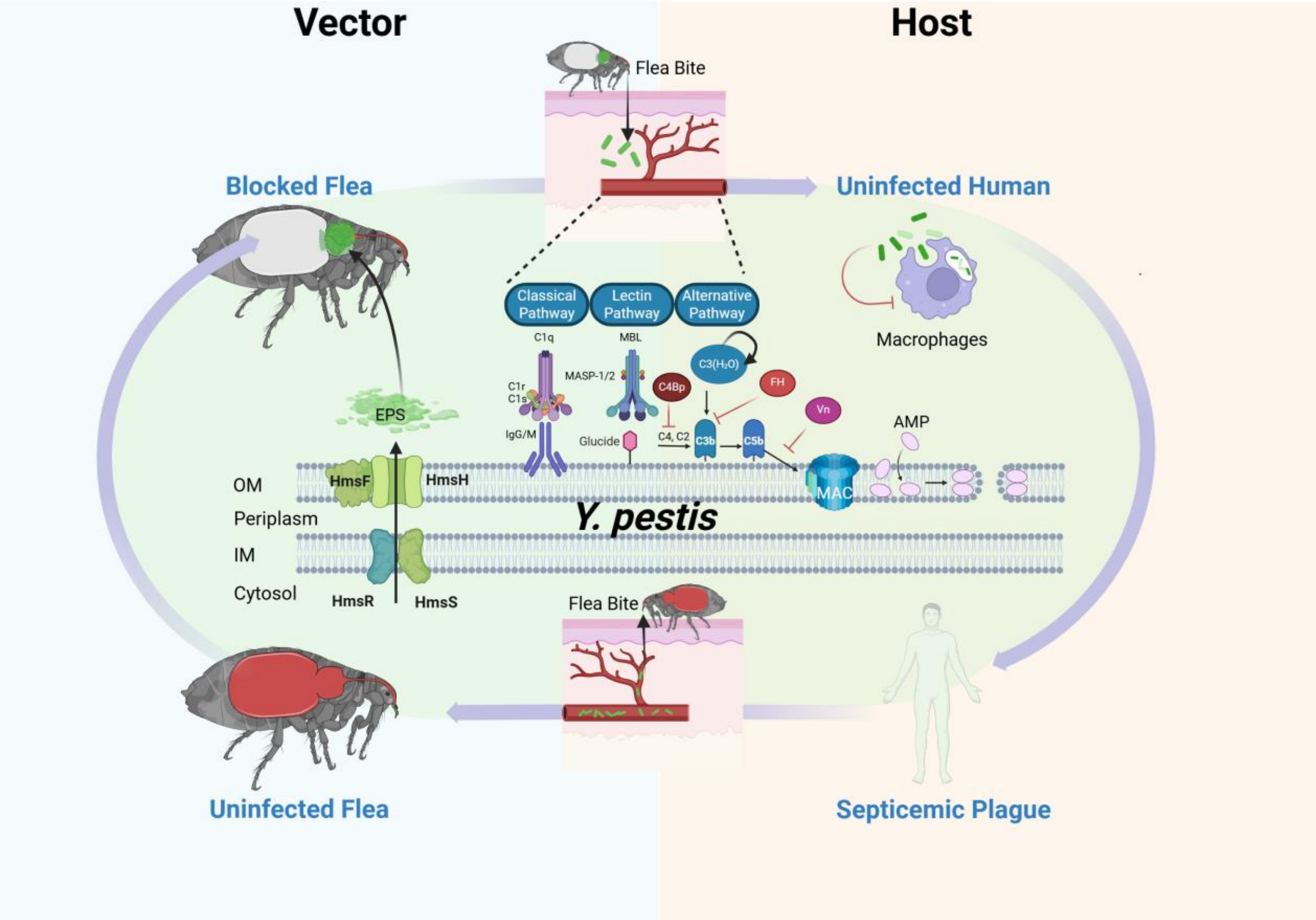

Supplementary Figure 1. Overview of the flea-mammal transmission cycle of *Yersinia pestis* and associated host environments. *Y. pestis* alternates between a flea vector and a mammalian host, two sharply distinct environments imposing opposing biological constraints. In the flea, proventricular colonization promotes HmsHFRS-dependent biofilm formation and foregut blockage, whereas in the mammalian host the bacterium must withstand innate immune defenses to establish bacteremia, enabling uptake by naive fleas. This schematic provides a conceptual framework linking flea-stage biofilm capacity with mammalian-stage resistance to innate immunity, the two processes investigated in this study. IM, inner membrane; OM: outer membrane. Created in BioRender. Alexandre, B. (2026) https://BioRender.com/yfvyrdt.

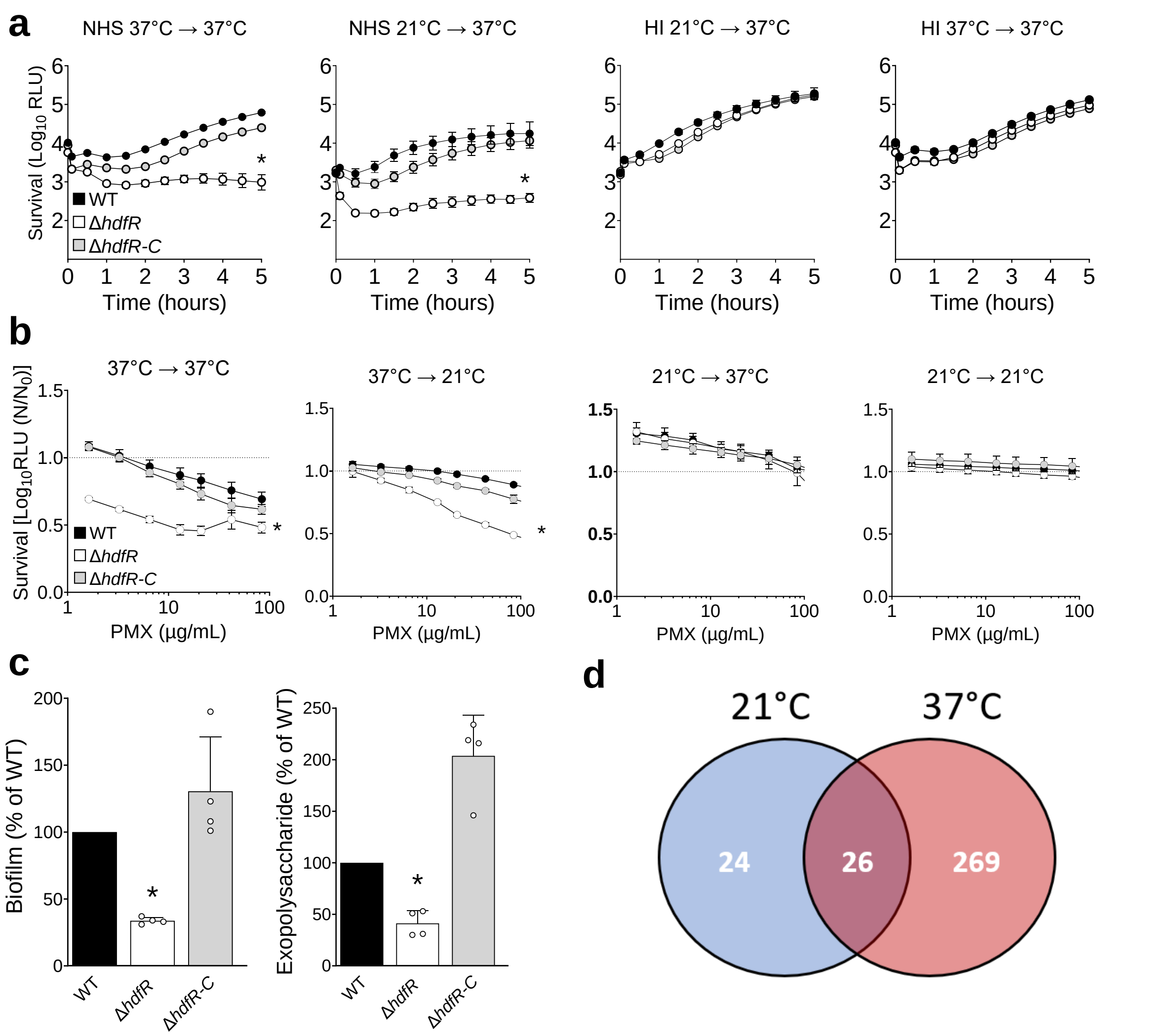

Supplementary Figure 2. HdfR contributes to multiple phenotypes across flea- and mammal-like conditions. (**A-B**) Mean ± SD survival of WT, Δ*hdfR*, Δ*maoP*, and complemented (-c) strains after exposure to normal human serum (NHS) or heat-inactivated serum (HI) (A) or polymyxin B (B) (n = 3 per strain and condition). Survival in serum (A) was quantified as Log_{10} RLU over time using bacteria grown at 21°C or 37°C, then exposed to serum at 37°C. Survival to polymyxin B was quantified as Log_{10} RLU (N/N_0) using bacteria grown at 21°C or 37°C, then exposed to increasing concentrations of polymyxin B (PMX) at either 21°C or 37°C, where N corresponds to luminescence after exposure and N_0 to luminescence before exposure. (**C**) Biofilm formation after a 24-h incubation at 21°C and Congo red binding (used as a proxy for exopolysaccharide production), expressed as a percentage relative to WT (mean ± SD, n = 4 per strain and assay). (**D**) Venn diagram showing the number of proteins differentially produced in the Δ*hdfR* compared to the WT grown at 21°C or 37°C (n = 5 independent bacterial cultures per condition). Here, n denotes independent biological experiments for (A–C) and independent bacterial cultures for (D). Asterisks denote statistically significant differences. For (A) and (B), survival curves were compared using two-way ANOVA followed by Tukey's multiple comparisons test for the indicated curve comparisons. For (C), biofilm formation and Congo red binding were analyzed separately using way ANOVA followed by Dunnett's multiple comparisons test with WT as the control. The P

values for the comparisons indicated by asterisks were: (A), in NHS, WT versus Δ*hdfR* after growth at either 21°C or 37°C, all $P < 0.0001$; (B), WT versus Δ*hdfR* after growth at 37°C and exposure to PMX at either 21°C or 37°C, all $P < 0.0001$; and (C), for biofilm formation, WT versus Δ*hdfR*, $P = 0.0059$, and for Congo red binding, WT versus Δ*hdfR*, $P = 0.0124$; WT versus Δ*hdfR*-c, $P = 0.0003$.

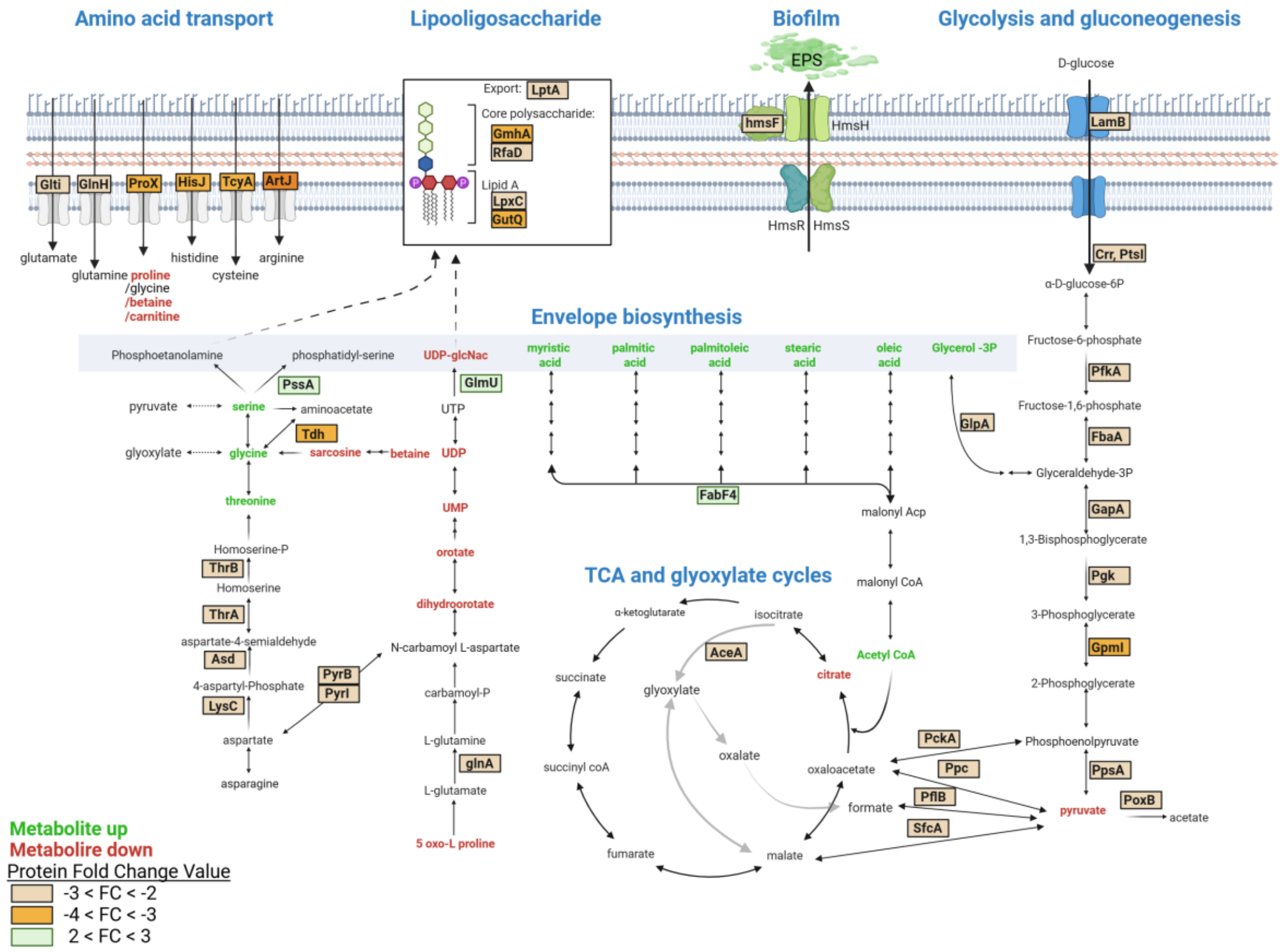

Supplementary Figure 3. Metabolic pathways affected by loss of HdfR. Schematic representation of central metabolic pathways highlighting proteins and metabolites whose abundance differs between WT and Δ*hdfR* grown at 37°C, as determined by comparative proteomic and metabolomic analyses. Proteins and metabolites increased in Δ*hdfR* relative to WT are shown in green, whereas those decreased are shown in red. Fold-change categories for protein abundance are indicated in the color legend. EPS: Extracellular polymeric substrance. Created in BioRender. Alexandre, B. (2026) https://BioRender.com/rhzmf1j.

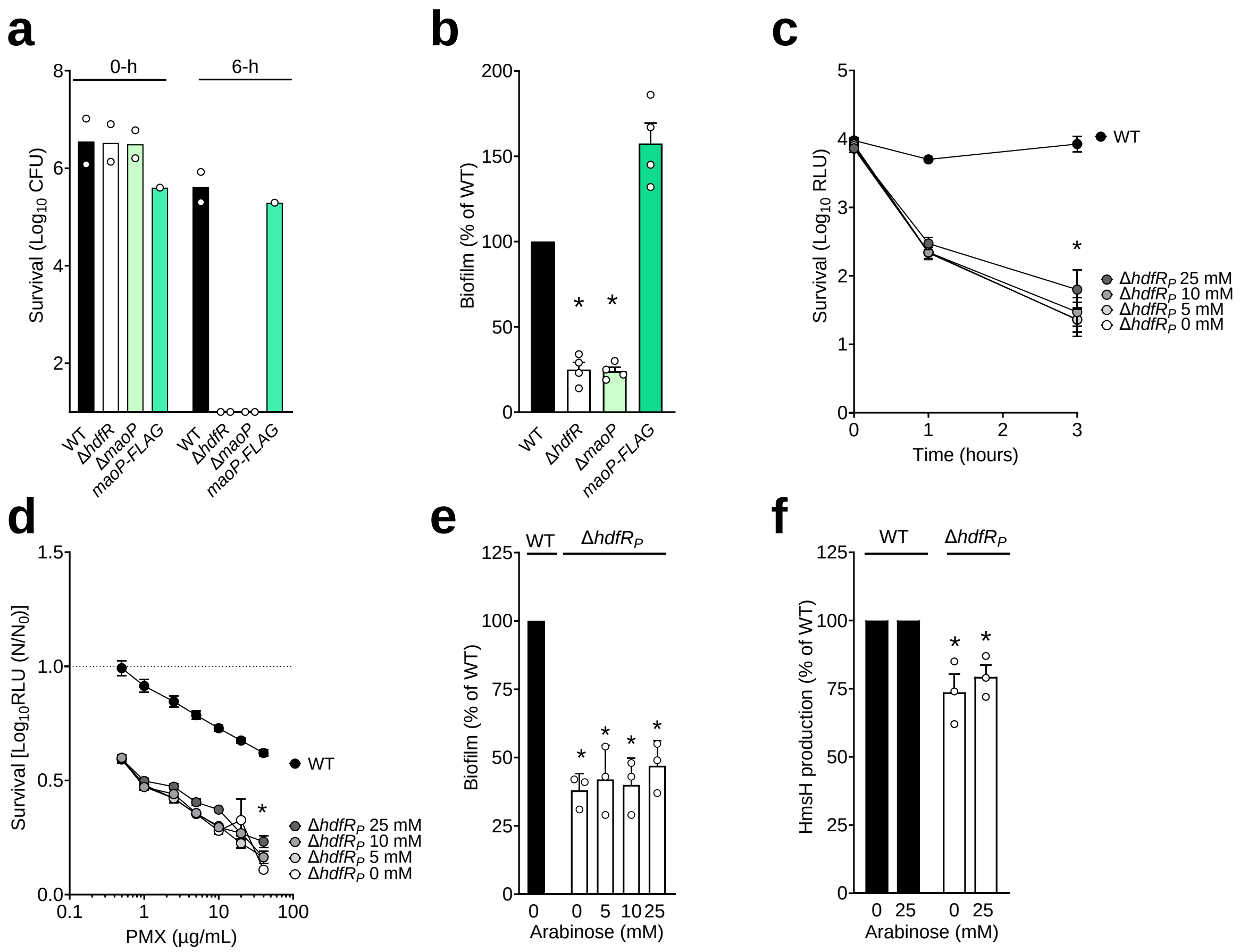

Supplementary Figure 4. Control and validation experiments for MaoP-FLAG functionality and arabinose-inducible expression systems. (**A**) Mean ± SD survival of Δ*maoP* and *maoP*-FLAG strains after 6 hours exposure to normal human serum, quantified as Log_{10} CFU (n = 2 per strain per condition; except for the *maoP*-FLAG strain, n = 1), demonstrating that C-terminal FLAG tagging does not impair MaoP-dependent serum resistance. (**B**) Biofilm formation after a 24-h incubation at 21°C by WT and Δ*maoP* strains, and a Δ*maoP* strain expressing MaoP-FLAG (FLAG tag fused to the C-terminus of MaoP), expressed as a percentage relative to WT (mean ± SD, n = 4 per strain), confirming functional complementation by MaoP-FLAG. (**C-D**) Mean ± SD survival of WT, Δ*hdfR* strains harboring an empty arabinose-inducible plasmid (pBAD30; $_{P}$) grown in the presence of increasing arabinose concentrations and exposure to normal human serum (C) or polymyxin B (D). These experiments control for potential effects of the empty plasmid and arabinose on serum and antimicrobial peptide resistance assays shown in **Fig. 1H and 1I**. Survival in serum (C) was quantified as Log_{10} RLU over time using bacteria grown at 21°C, then exposed to serum at 37°C (n = 3 per strain and condition). Survival to polymyxin B (D) was quantified as Log_{10} RLU (N/N_0) using bacteria grown at 37°C, then exposed to increasing concentrations of polymyxin B (PMX) at 37°C, where N corresponds to luminescence after exposure and N_0 to luminescence before exposure (n = 3 ; per strain and condition). (**E**) Biofilm formation after a 24-h incubation at 21°C by WT and Δ*hdfR* strains harboring an empty arabinose-inducible plasmid, grown in the presence of increasing arabinose concentrations, expressed as a percentage relative to WT (mean ± SD, n = 3 per strain). This experiment served as controls for

Fig. 1J. (**F**) Mean ± SD production of HmsH in WT and Δ*hdfR* harboring an empty arabinose-inducible plasmid grown in the absence or presence of arabinose (25mM) (n = 3 per strain). HmsH levels, normalized to total protein (Coomassie blue staining) and expressed relative to WT, were quantified by immunoblotting. This experiment served as a control for **Fig. 2D**. Here, n denotes independent biological experiments for (A-E) and independent biological experiments analyzed by immunoblotting from independently prepared bacterial samples for (F). Asterisks denote statistically significant differences. comparisons test. For (C) and (D), survival curves were compared using two-way ANOVA followed by Tukey's multiple comparisons test. For (E) and (F), comparisons were assessed using one-way ANOVA followed by Tukey's multiple comparisons test. The P values for the comparisons indicated by asterisks were: (B), WT versus Δ*hdfR* and Δ*maoP*, both $P < 0.0001$, and WT versus *maoP*-FLAG, $P = 0.0001$; (C-E), WT versus each Δ$hdfR_p$ condition (0, 5, 10 and 25 mM), all $P < 0.0001$ (F), WT 0 mM versus Δ$hdfR_p$ 0 mM, $P = 0.0067$, and WT 0 mM versus Δ$hdfR_p$ 25 mM, $P = 0.0255$.

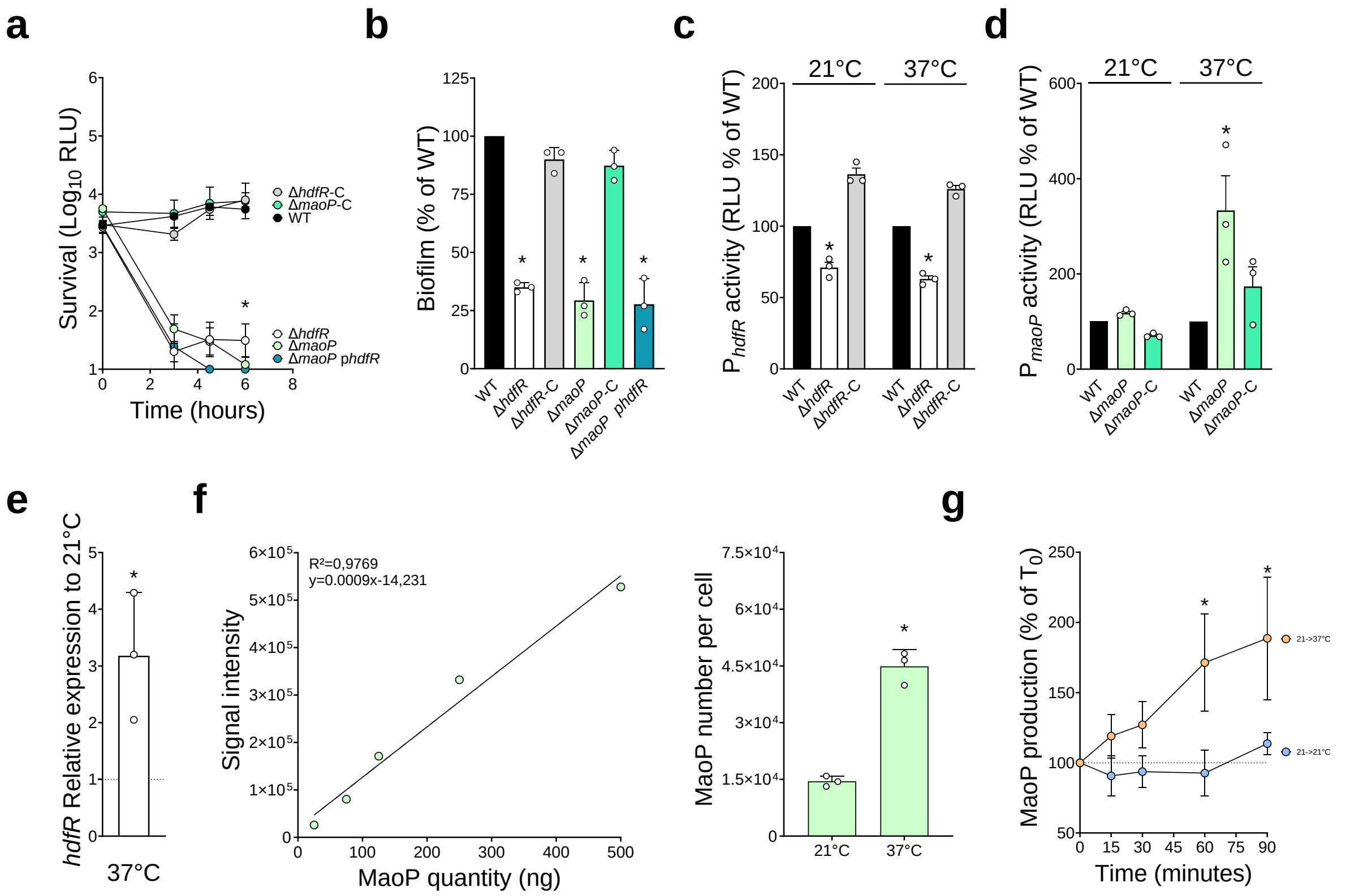

Supplementary Figure 5. Hierarchical, autoregulation and temperature-dependent control of the HdfR-MaoP module. (**A**) Mean ± SD survival of WT, Δ*hdfR*, Δ*maoP* and complemented (-c) strains, and of Δ*maoP* mutant expressing *hdfR* from high copy number plasmid grown at 21°C and assayed at 37°C after exposure to Normal human serum (NHS) over time, showing that HdfR overexpression does not restore MaoP-dependent serum resistance. Data are from n = 3 independent biological experiments for WT and n = 4 for the other strains. (**B**) Biofilm formation after a 24-h incubation at 21°C, expressed as a percentage relative to WT (mean ± SD, n = 3 per strain), indicating that HdfR overexpression cannot compensate for MaoP loss in biofilm formation. (**C**) Mean ± SD *hdfR* promoter activity in WT, Δ*hdfR*, and complemented (-c) strains cultured at 21°C or 37°C and carrying a chromosomal *hdfR*-lux reporter, expressed as Log_{10} Relative light unit (RLU) relative to WT (n = 3 per strain and temperature), demonstrating autoregulation of *hdfR* expression (**D**) Mean ± SD *maoP* promoter activity in WT, Δ*maoP*, and complemented strains cultured at 21°C or 37°C and carrying a chromosomal *maoP*-lux reporter, expressed as RLU relative to WT (n = 3 per strain), demonstrating autoregulation of *maoP* expression. (**E**) Mean ± SD relative *hdfR* expression at 37°C compared with 21°C, measured by RT-qPCR (n = 3 per condition). (**F**) Mean ± SD estimated MaoP abundance per cell after incubation at 21°C or 37°C (n = 3 per condition), together with the standard curve used for MaoP quantification. (**G**) Mean ± SD relative MaoP abundance at different time points after temperature shifts from 21°C to 21°C or from 21°C to 37°C (n = 3 per condition). Here, n denotes independent biological experiments for (A-E), and independent biological experiments analyzed by immunoblotting from independently prepared bacterial samples for (F-G). Asterisks denote statistically significant differences. For (A), survival curves were compared using two-way ANOVA followed by Dunnett's multiple comparisons test. For (B-D), comparisons were assessed using one-way ANOVA followed by

Dunnett's multiple comparisons test. For (E), relative *hdfR* expression at 37°C versus 21°C was analyzed after log2 transformation using a two-sided one-sample t-test against 0. For (F), MaoP abundance at 21°C and 37°C was compared using two-sided paired t-test For (G), MaoP abundance after temperature shift was compared using two-way ANOVA followed by Šídák's multiple comparisons test. The P values for the comparisons indicated by asterisks were: (A), WT versus Δ*hdfR*, Δ*maoP* and Δ*maoP* p*hdfR*, all P < 0.0001; (B), WT versus Δ*hdfR*, Δ*maoP* and Δ*maoP* p*hdfR*, all P < 0.0001; (C), at 21°C, WT versus Δ*hdfR*, P = 0.0014, and at 37°C, WT versus Δ*hdfR*, P < 0.0001; (D), at 37°C, WT versus Δ*maoP*, P < 0.0001; (E), relative *hdfR* expression at 37°C versus 21°C, P = 0.0066 (F), 21°C versus 37°C, P = 0.0039; and (G), 21°C to 21°C versus 21°C to 37°C, P = 0.0008 at 60 min and P = 0.0013 at 90 min.

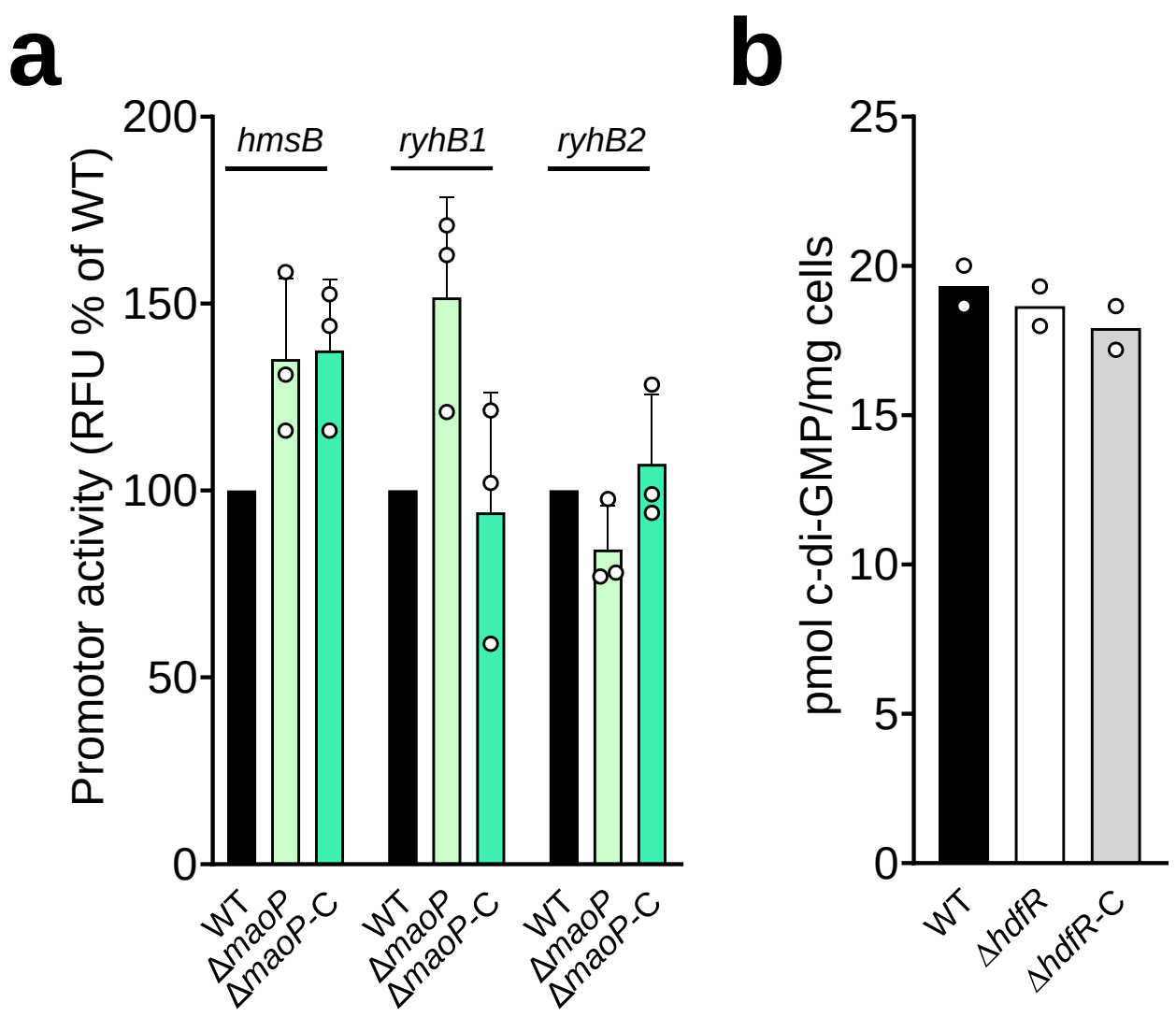

Supplementary Figure 6. The HdfR-MaoP module does not affect sRNA- or c-di-GMP-dependent regulation of HmsHFRS in *Y. pestis* microtus. (**A**) Mean ± SD promoter activity of genes encoding sRNAs previously reported to activate *hmsHFRS* and *hmsT* translation, measured in WT and Δ*maoP* strains cultured at 21°C and carrying transcriptional GFP reporters. Data are expressed as relative fluorescence units (RFU) relative to WT Data are from n = 3 independent biological experiments per strain. (**B**) Mean ± SD c-di-GMP levels in WT, Δ*hdfR* and complemented (-c) strains cultured at 21°C. Data are from n = 2 independent measurements from independently prepared bacterial samples per strain. For (A), promoter activities were analyzed separately for each reporter using one-way ANOVA followed by Dunnett's multiple comparisons test with WT as the control. No statistically significant differences were detected.

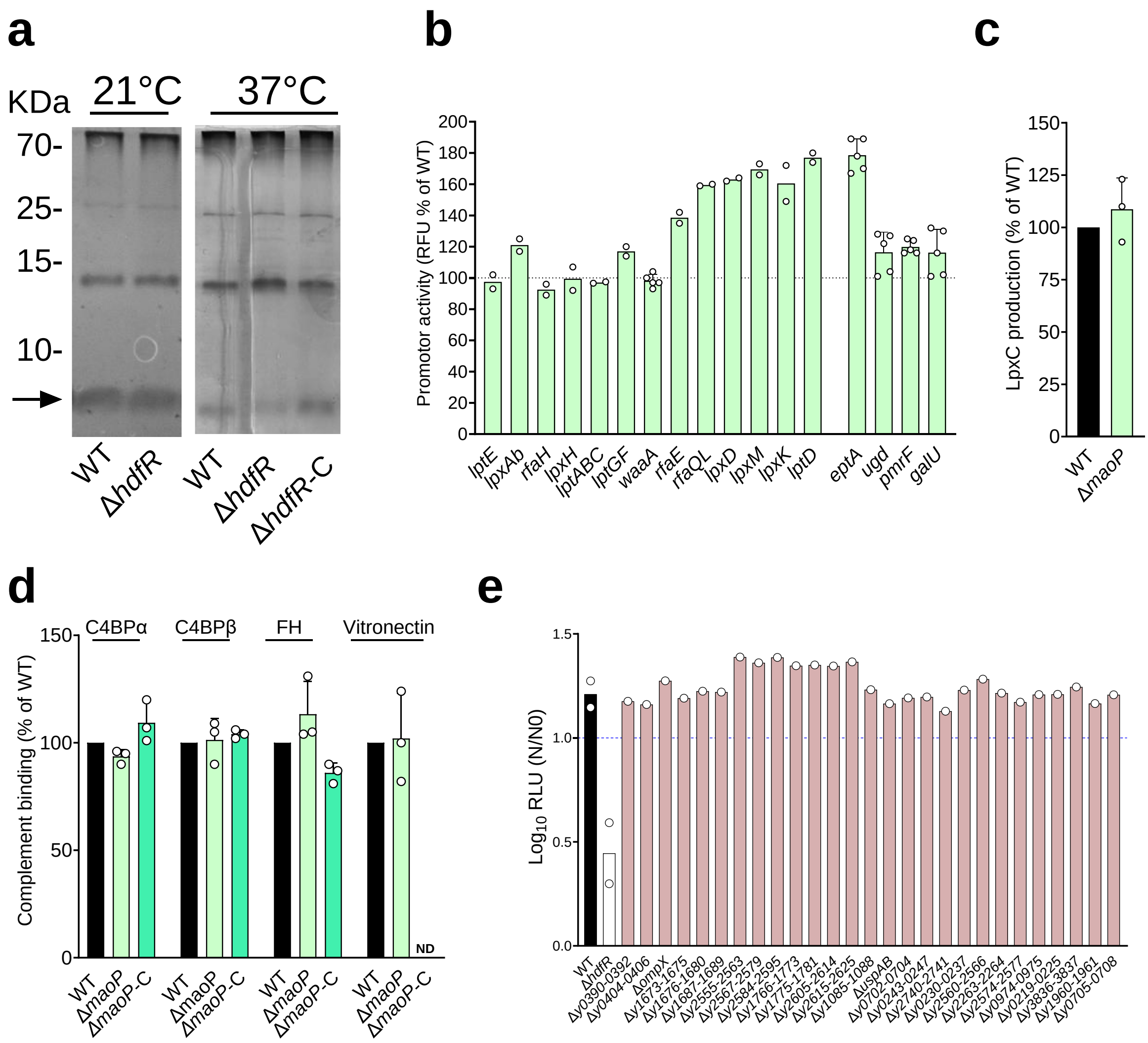

Supplementary Figure 7. MaoP- and HdfR-dependent phenotypes are not associated with global alterations in LOS biosynthesis, complement inhibitor recruitment, or individual omics-based candidate genes. (**A**) DOC-PAGE analysis of LOS from WT, Δ*hdfR* and complemented (-c) strains grown at 21°C or 37°C. Equal amounts of bacterial material were loaded. No difference was observed under flea-like (21°C) conditions, whereas a decrease in LOS quantity was observed in Δ*hdfR* under mammalian host-like (37°C) conditions. The DOC-PAGE analysis was performed once. (**B**) Mean ± SD promoter activity of genes involved in LOS biosynthesis and export pathways, as well as canonical lipid A modification pathways associated with polymyxin resistance, in WT and Δ*maoP* strains cultured at 37°C and carrying *gfp* reporters, expressed as relative fluorescence units (RFU) relative to WT. Data are from n = 2 independent biological experiments per strain for most reporters, except for *waaA*, *eptA*, *ugd*, *pmrF* and *galU*, for which n = 5 independent biological experiments per strain. (**C**) The amount of LpxC produced in the mutant, relative to WT, is also given. Data are from n = 3 independent biological experiments analyzed by immunoblotting from independently prepared bacterial samples. No decrease in reporter activity or LpxC production was observed in Δ*maoP* relative to WT. (**D**) Immunoblot analysis of complement inhibitors (C4BP$_{\alpha}$, C4BP$_{\beta}$, FH, vitronectin) bound to WT, Δ*maoP*, and complemented (-c) strains grown at 21°C and after a 60-min incubation with serum at 37°C. Bars indicate the mean ± SD relative to WT band intensity.

Data are from n = 3 independent biological experiments analyzed by immunoblotting from independently prepared bacterial samples. (**E**) Mean ± SD survival of WT (n = 2), Δ*hdfR* (n = 2), and mutants lacking genes identified as putatively regulated by the HdfR-MaoP module in the present omics analysis, after exposure to normal human serum. Survival was quantified as Log_{10} RLU (N/N_0) using bacteria grown at 21°C and then exposed to serum at 37°C for 180 min, where N corresponds to luminescence after exposure and N_0 to luminescence before exposure. Data are from n = 2 independent biological experiments for WT and Δ*hdfR*, and n = 1 for the other mutants, which are shown descriptively.

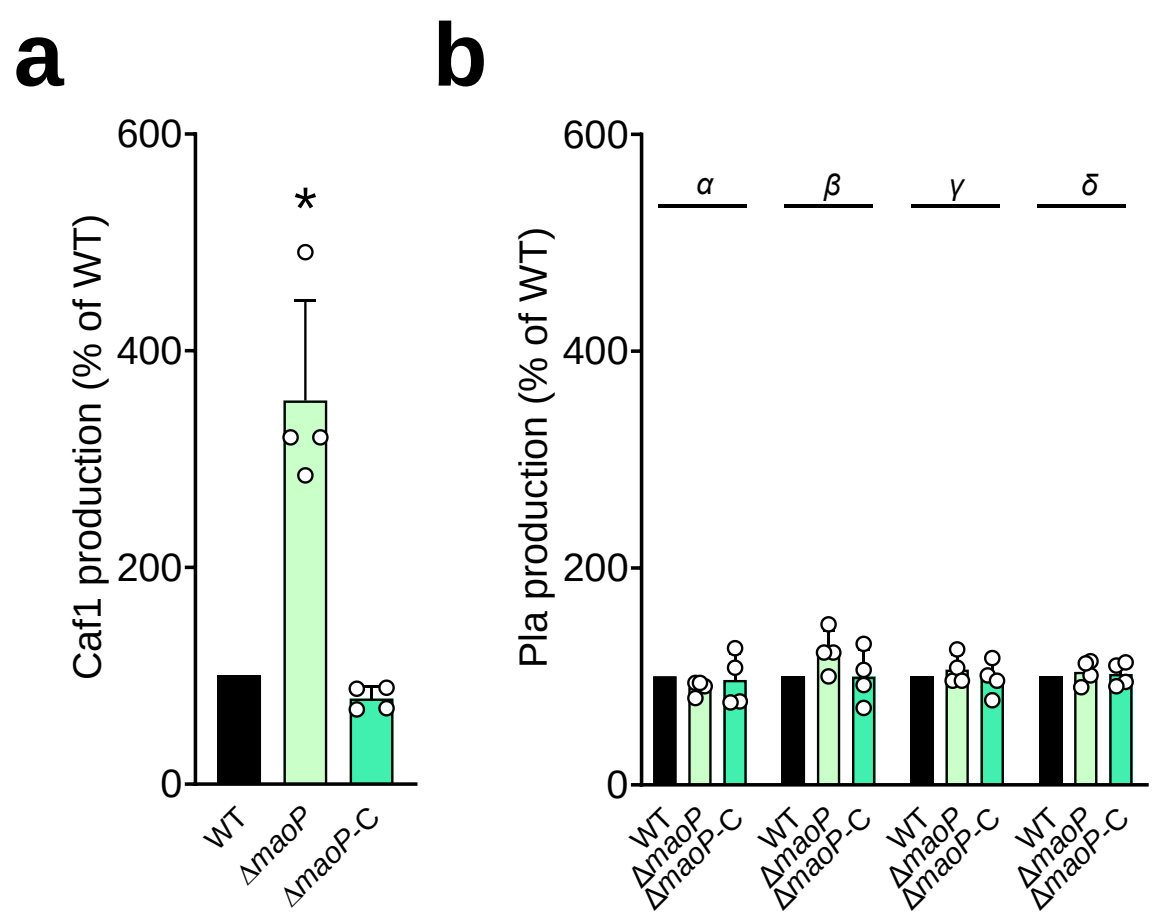

Supplementary Figure 8. MaoP affects Caf1 production but not Pla production. (**A-B**) Relative amounts of Caf1 (A) and Pla forms (B) produced in the Δ*maoP* and complemented (-c) strains compared with WT, measured by immunoblot. Mean ± SD estimated abundance per cell from n = 4 independently prepared bacterial samples analyzed by immunoblotting. For (A), Asterisks denote statistically significant differences using one-way ANOVA followed by Dunnett's multiple comparisons test, WT versus Δ*maoP*, P = 0.0002.

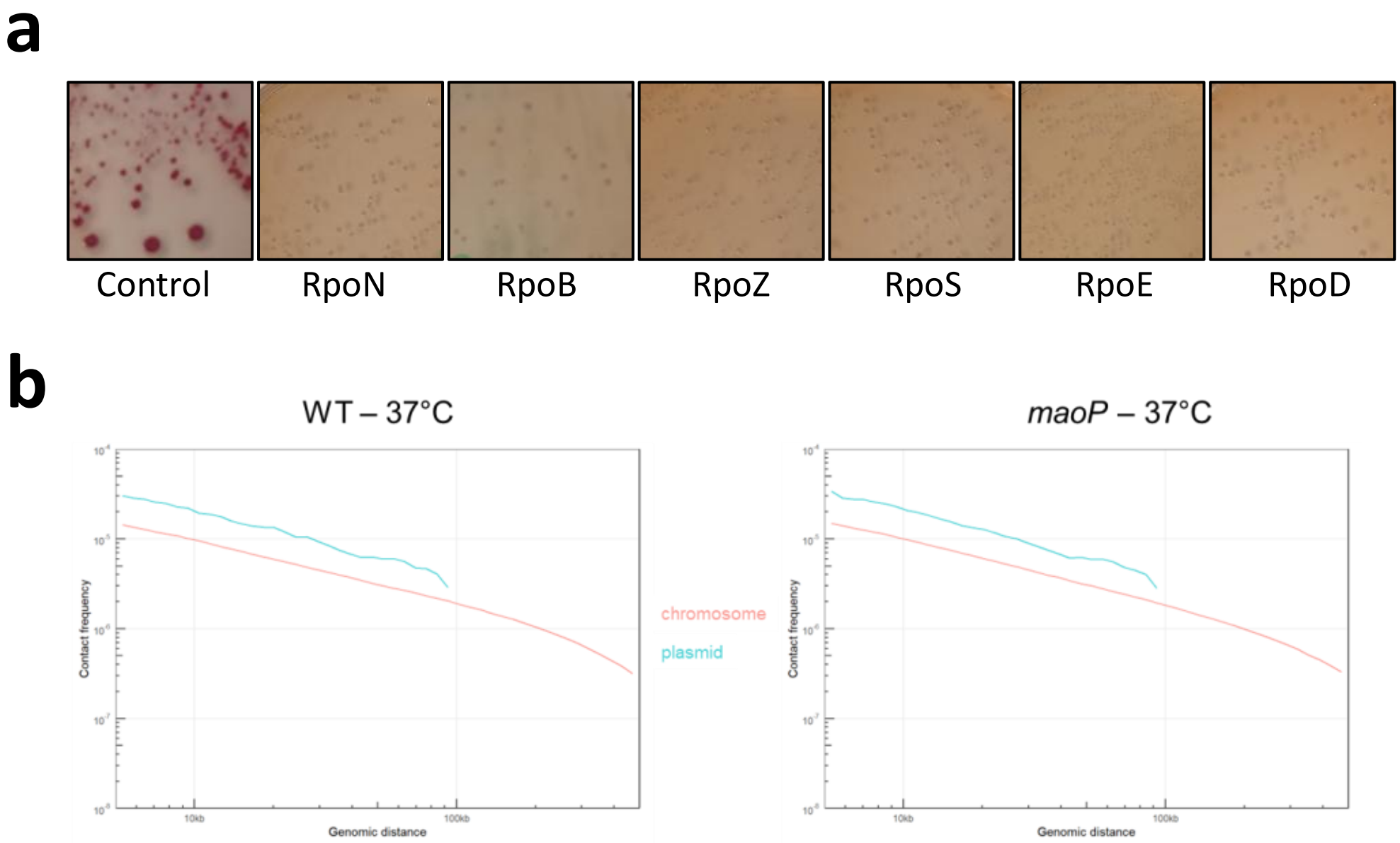

Supplementary Figure 9. MaoP does not detectably interact with RNA polymerase or alter large-scale chromosome organization. (**A**) Bacterial two-hybrid analysis testing interactions between MaoP and RNA polymerase subunits or sigma factors. Representative colonies are shown for the indicated combinations. The Zip-Zip pair was used as a positive control. No interaction was detected between MaoP and the tested RNA polymerase components under these conditions. This experiment was performed once. (**B**) Contact-probability decay as a function of genomic distance calculated from normalized Hi-C contact matrices of WT Δ*pla* and Δ*maoP* Δ*pla* strains grown at 37°C. Curves are shown separately for chromosome and plasmid contacts. No major difference in contact-probability decay was observed between WT and Δ*maoP*, supporting the absence of detectable large-scale chromosome organization defects under the conditions tested.

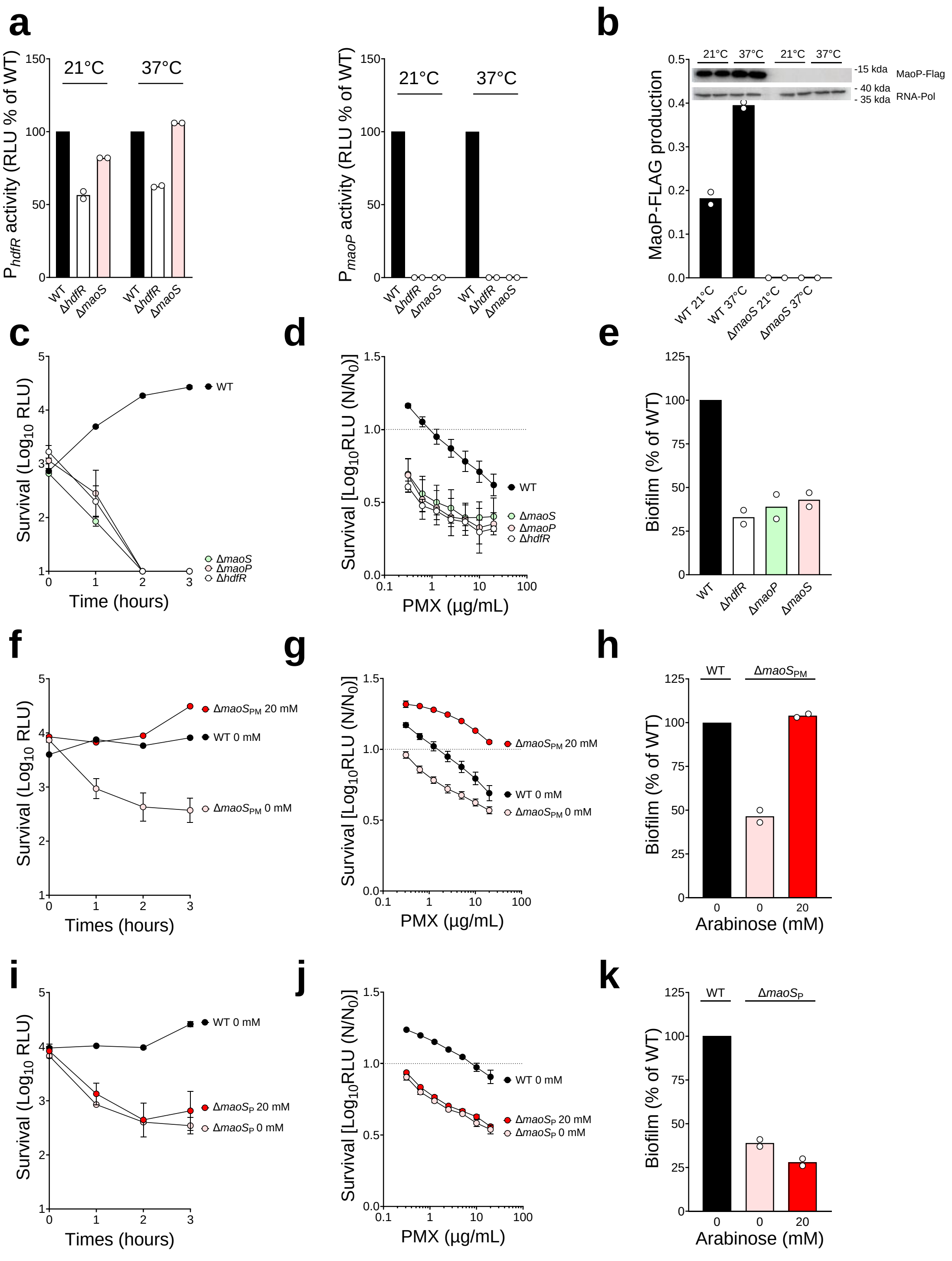

Supplementary Figure 10. Functional consequences of MaoS binding site deletion on MaoP-dependent phenotypes. (**A**) Mean ± SD *hdfR* and *maoP* promoter activity in WT, Δ*hdfR* and ΔMaoS-site strains cultured at 21°C or 37°C and carrying a chromosomal *hdfR*- or *maoP*-lux reporter, expressed as Log_{10} RLU relative to WT. Data are from n = 2 independent biological experiments per strain and temperature. (**B**) Immunoblot analysis of total protein extracts from WT and ΔMaoS-site strains expressing a functional MaoP-FLAG (Supplementary Fig. 3a and 3b) and grown at 21°C or 37°C. Equal amounts of protein were loaded. Bars indicate the mean ± SD relative intensity of MaoP-FLAG bands normalized to the RNA polymerase signal. Data are from n = 2 independent biological experiments analysed by immunoblotting

from independently prepared bacterial samples per strain and temperature. (**C-D**) Mean ± SD survival of WT, Δ*hdfR*, Δ*maoP* and ΔMaoS-site strains after exposure to normal human serum (C) or polymyxin B (D). Survival in serum (C) was quantified as Log_{10} RLU over time using bacteria grown at 21°C, then exposed to serum at 37°C. Survival to polymyxin B (D) was quantified as Log_{10} RLU (N/N_0) using bacteria grown at 37°C, then exposed to increasing concentrations of polymyxin B (PMX) at 37°C, where N corresponds to luminescence after exposure and N_0 to luminescence before exposure. Data are from n = 2 independent biological experiments per strain, except for panel (D), which includes n = 3 independent biological experiments per strain. (**E**) Biofilm formation after a 24-h incubation at 21°C, expressed as a percentage relative to WT. Data are shown as mean ± SD from n = 2 independent biological experiments per strain. (**F-G**) Same experimental setup as in (C-D), except that the ΔMaoS-site strain expressing or not *maoP* from an arabinose-inducible promoter (pBAD30-*maoP*-FLAG; $_{PM}$) was used and grown in absence or presence of arabinose (20 mM) prior to serum (F) or polymyxin B (G) exposure. Survival was quantified as in (C-D). Data are shown as mean ± SD from n = 2 independent biological experiments per strain and condition. (**H**) Biofilm formation under the same conditions as in (E), except that the ΔMaoS-site strain expressing or not *maoP* from an arabinose-inducible promoter (pBAD30-*maoP*-FLAG; $_{PM}$) was used and grown in the absence or presence of arabinose (20 mM). Data are shown as mean ± SD from n = 2 independent biological experiments per strain. (**I-K**) Same experimental setup as (F-H), expect that ΔMaoS-Site strain harbors an empty arabinose-inducible promoter (pBAD30; $_{P}$). Data are shown as mean ± SD from n = 2 per strain and condition. This experiment served as controls of **panels F-H**.

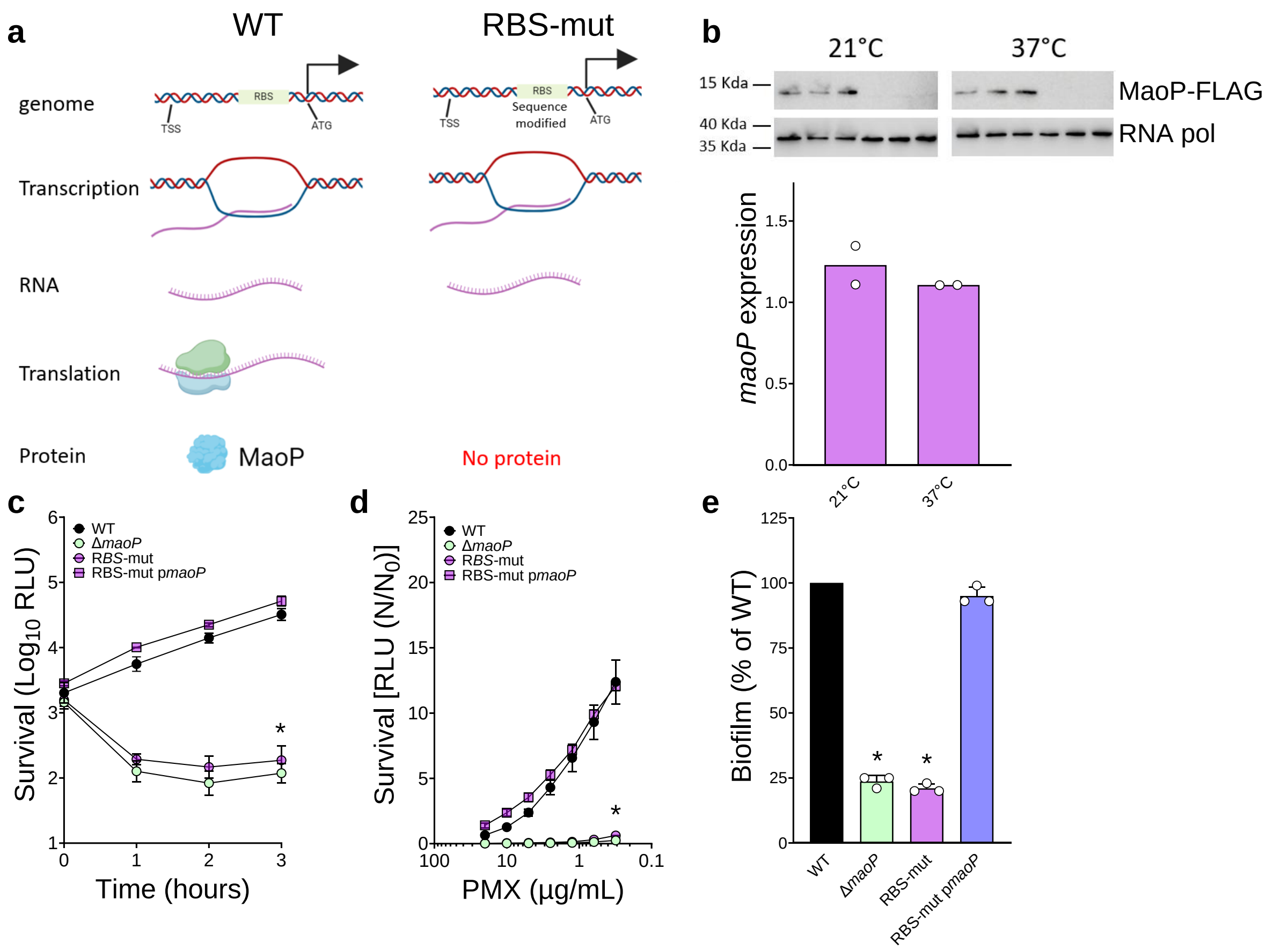

Supplementary Figure 11. *maoP* must be translated to exert its activity. (**A**) Schematic representation of the experimental strategy used to disrupt *maoP* translation by mutating the ribosome-binding site (indicated RBS-mut) without affecting transcription. Created in BioRender. Alexandre, B. (2026) https://BioRender.com/yitdck3. (**B**) Presence of MaoP and mean ± SD *maoP* transcript levels in WT and RBS-mut strains (both having a *maoP* gene fused to a FLAG tag coding sequence) grown at 21°C or 37°C. Presence of MaoP were detected by immunoblot using total protein extracts and an anti-FLAG antibody (top). Detection of RNA polymerase was used as a loading control (bottom). *maoP* transcript levels were quantified by RT-qPCR and expressed relative to WT. Data are from n = 2 independent biological experiments per condition. (**C-D**) Mean ± SD survival of WT, Δ*maoP*, RBS-mut, and the RBS-mut strain complemented with *maoP* after exposure to normal human serum (C) or polymyxin B (D). Survival in serum (C) was quantified as Log_{10} RLU over time using bacteria grown at 21°C, then exposed to serum at 37°C. Survival to polymyxin B (D) was quantified as Log_{10} RLU (N/N_0) using bacteria grown at 37°C, then exposed to increasing concentrations of polymyxin B (PMX) at 37°C, where N corresponds to luminescence after exposure and N_0 to luminescence before exposure. Data are from n = 3 independent biological experiments per strain. (**E**) Biofilm formation after a 24-h incubation at 21°C, expressed as a percentage relative to WT. Data are shown as mean ± SD from n = 3 independent biological experiments per strain. Here, n denotes independent biological experiments for (B-E); the immunoblot in (B) was performed using independently prepared bacterial samples. Asterisks denote statistically significant differences. For (C) and (D), survival curves were compared using two-way ANOVA followed by Dunnett's multiple comparisons test with WT as the control. For (E),

biofilm formation was analyzed using one-way ANOVA followed by Dunnett's multiple comparisons test. For each assay, WT versus each of Δ*maoP* and RBS-mut, all $P < 0.0001$.

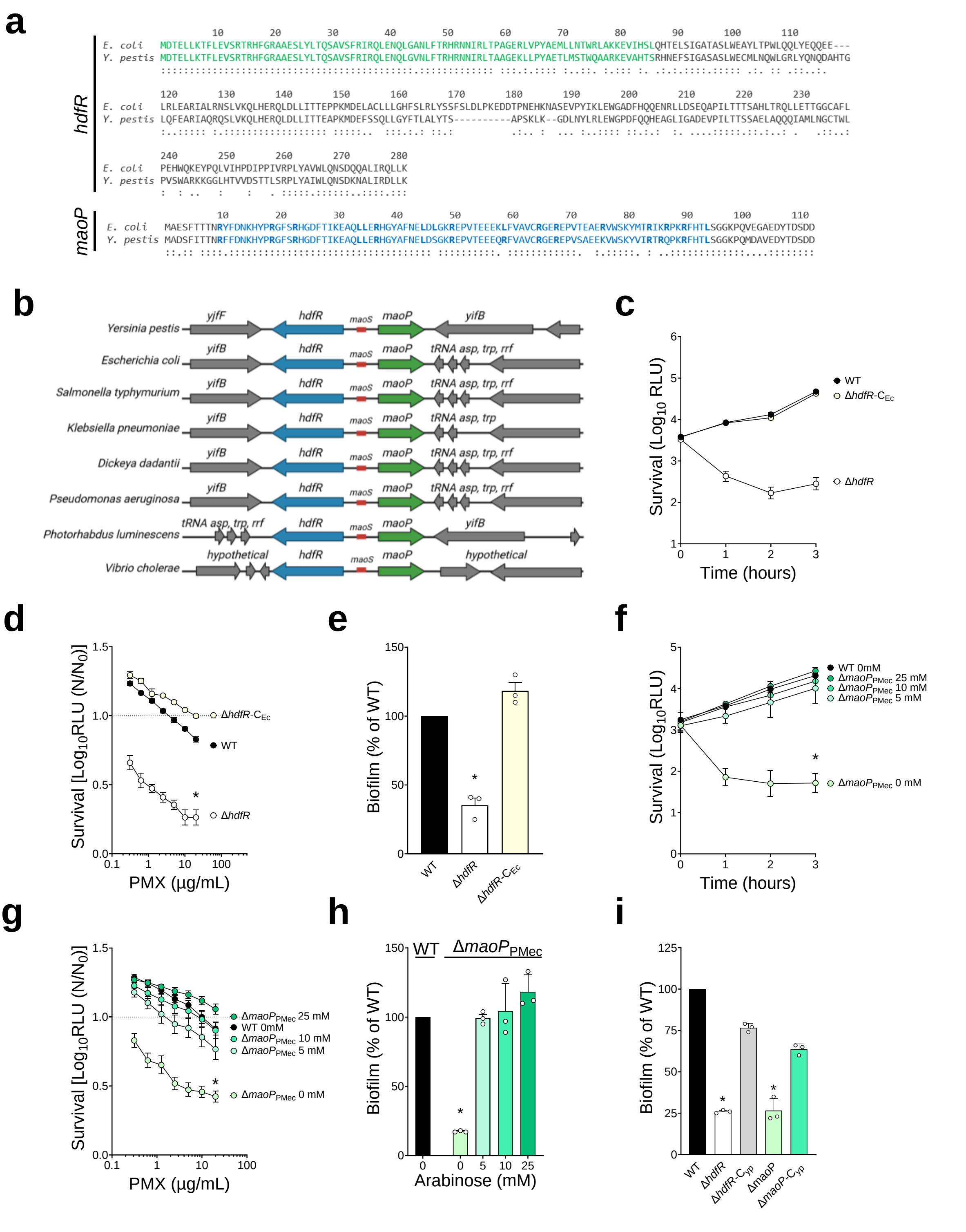

Supplementary Figure 12. Conservation of the HdfR-MaoP module across diverse bacterial species and cross-species functional complementation between *Y. pestis* and *E. coli*. (**A**) Pairwise sequence alignments of HdfR and MaoP from *Y. pestis* and *E. coli*. Conserved residues are indicated, with the helix-turn-helix (HTH) DNA-binding domain of HdfR highlighted in green and the predicted basic central region of MaoP highlighted in blue. (**B**) Genomic synteny of the *hdfR-maoP* gene pair in representative Gammaproteobacteria, illustrating conserved gene organization and orientation across species. Created in BioRender. Alexandre, B. (2026) https://BioRender.com/s0fs90v. (**C-D**) Mean ± SD survival of *Y. pestis*

WT, Δ*hdfR*, and Δ*hdfR* complemented with 6xHis-tagged *hdfR* from *E. coli* (-c_{Ec}) strains after exposure to normal human serum (C) or polymyxin B (D). Survival in serum (C) was quantified as Log_{10} RLU over time using bacteria grown at 21°C, then exposed to serum at 37°C (n = 2 per strain and time point). Survival to polymyxin B (D) was quantified as Log_{10} RLU (N/N_0) using bacteria grown at 37°C, then exposed to increasing concentrations of polymyxin B (PMX) at 37°C, where N corresponds to luminescence after exposure and N_0 to luminescence before exposure (n = 3 strain and condition). (**E**) Mean ± SD biofilm formation after a 24-h incubation at 21°C by *Y. pestis* WT, Δ*hdfR* and Δ*hdfR* complemented with 6xHis-tagged *hdfR* from *E. coli*, expressed as a percentage relative to WT (n = 3 per strain). (**F-G**) Same experimental setup as in (C-D), except that the Δ*maoP* strain expressing or not *E. coli maoP*-3FLAG (-c_{Ec}) from an arabinose-inducible promoter (PM_{ec}) was used and grown in the presence of increasing arabinose concentrations prior to serum (F) or polymyxin B (G) exposure. Survival was quantified as in (C-D) (n = 3 per strain and condition). (**H**) Biofilm formation under the same conditions as in (E), except that the Δ*maoP* strain expressing or not *E. coli maoP* (-c_{Ec}) from an arabinose-inducible promoter (PM_{ec}) were used and grown in the presence of increasing arabinose concentrations (mean ± SD, n = 3 per strain). (**I**) Biofilm formation after a 24-h incubation at 37°C by *E. coli* WT, Δ*hdfR*, and Δ*maoP* strains, with the mutant strains complemented with their respective *Y. pestis* orthologs (-C_{Yp}), expressed as a percentage relative to WT (mean ± SD, n = 3 per strain). Here, n denotes independent biological experiments. Asterisks denote statistically significant differences. For (F) and (G), survival curves were compared using two-way ANOVA followed by Tukey's multiple comparisons test for the indicated curve comparisons. For (E), (H) and (I), comparisons were assessed using one-way ANOVA followed by Tukey's multiple comparisons. The P values for the comparisons indicated by asterisks were: (E), WT versus Δ*hdfR*, P = 0.0001; (F-H), Δ*maoP*$_{PMec}$ 0 mM versus WT and versus each arabinose-induced Δ*maoP*$_{PMec}$ condition, all P < 0.0001; and (I), WT versus each Δ*hdfR* and Δ*maoP* mutant, all P < 0.0001.

Supplementary information references

1. Schlegel, S., P. de Gier, J. W. De-convoluting the Genetic Adaptations of *E. coli* C41(DE3) in Real Time Reveals How Alleviating Protein Production Stress Improves Yields. Cell Rep **10**, 1758-1766 (2015).
2. Pradel, E. *et al.* New insights into how *Yersinia pestis* adapts to its mammalian host during bubonic plague. *PLoS Pathog* **10**, e1004029 (2014).
3. Quintard, K. *et al*. Evaluation of the Role of the *opgGH* Operon in *Yersinia pseudotuberculosis* and Its Deletion during the Emergence of *Yersinia pestis*. Infect Immun **83**, 3638-3647 (2015).
4. Dewitte, A. *et al.* A refined model of how *Yersinia pestis* produces a transmissible infection in its flea vector. *PLoS Pathog* **16**, e1008440, (2020).
5. Bouvenot, T. *et al*. Interplay between *Yersinia pestis* and its flea vector in lipoate metabolism. ISME J **15**, 1136-1149 (2021).
6. Datsenko, K. A. & Wanner, B. L. One-step inactivation of chromosomal genes in *Escherichia coli* K-12 using PCR products. Proc Natl Acad Sci U S A **97**, 6640-6645 (2000).
7. Choi, K. H. *et al.* A Tn*7*-based broad-range bacterial cloning and expression system. *Nat Methods* **2**, 443-448 (2005).
8. Stenum, T. S. *et al*. RNA interactome capture in *Escherichia coli* globally identifies RNA-binding proteins. Nucleic Acids Res **51**, 4572-4587 (2023).
9. Koch, L. *et al.* Transcriptomic studies and assessment of *Yersinia pestis* reference genes in various conditions. *Sci Rep* **9**, 2501 (2019).
10. Simon, S. *et al*. Fast and simple detection of *Yersinia pestis* applicable to field investigation of plague foci. PLoS One **8**, e54947 (2013).
