# Supplementary material for: A dual-host regulatory hub integrating biofilm formation and innate immunity resistance
Source: Nat Commun. 2026 Sep 1;17:10385. doi: 10.1038/s41467-026-77307-4 (PMC13627719; doi:10.1038/s41467-026-77307-4)
Supplement: Supplementary file 3 [file 41467_2026_77307_MOESM2_ESM.pdf]

Description of Additional Supplementary Files:

Supplementary Data 1: The 200 most abundant *Y. pestis* WT proteins identified in our proteomic study. MaoP is highlited in green.

Supplementary Data 2: The MaoP's DNA binding sites by ChIP-Seq analysis. Genes of interest for the study are highlited in green. Peaks were called using MACS2 with default settings. MACS2-derived −log10(P value), fold enrichment and −log10(q value) are reported for each peak.

Supplementary Data 3: Strains and plasmids used in the study. Ap^R, Sm^R, Km^R, Tp^R, Tc^R mean resistance to ampicillin, streptomycin, kanamycin, trimethoprime, tetracycline respectively. References are provided in the Supplementary information.

Supplementary Data 4: Primer sets used in the study. References are provided in the Supplementary information.

Supplementary Data 5: Antibodies used in the study. (O/N / overnight). '10' corresponds to the reference 10 in the Supplementary information.

Supplementary Data 6: Reagent and resources used in the study.
