# Supplementary material for: A dual-host regulatory hub integrating biofilm formation and innate immunity resistance
Source: Nat Commun. 2026 Sep 1;17:10385. doi: 10.1038/s41467-026-77307-4 (PMC13627719; doi:10.1038/s41467-026-77307-4)
Supplement: Supplementary file 4 [file 41467_2026_77307_MOESM9_ESM.pdf]

Corresponding author(s): Florent Sebbane
Last updated by author(s): 07/07/2026

# Reporting Summary

Nature Portfolio wishes to improve the reproducibility of the work that we publish. This form provides structure for consistency and transparency in reporting. For further information on Nature Portfolio policies, see our Editorial Policies and the Editorial Policy Checklist.

## Statistics

For all statistical analyses, confirm that the following items are present in the figure legend, table legend, main text, or Methods section.

| n/a | Confirmed | |
|---|---|---|
| ☐ | ☒ | The exact sample size (*n*) for each experimental group/condition, given as a discrete number and unit of measurement |
| ☐ | ☒ | A statement on whether measurements were taken from distinct samples or whether the same sample was measured repeatedly |
| ☐ | ☒ | The statistical test(s) used AND whether they are one- or two-sided<br>*Only common tests should be described solely by name; describe more complex techniques in the Methods section.* |
| ☒ | ☐ | A description of all covariates tested |
| ☐ | ☒ | A description of any assumptions or corrections, such as tests of normality and adjustment for multiple comparisons |
| ☐ | ☒ | A full description of the statistical parameters including central tendency (e.g. means) or other basic estimates (e.g. regression coefficient) AND variation (e.g. standard deviation) or associated estimates of uncertainty (e.g. confidence intervals) |
| ☐ | ☒ | For null hypothesis testing, the test statistic (e.g. *F*, *t*, *r*) with confidence intervals, effect sizes, degrees of freedom and *P* value noted<br>*Give P values as exact values whenever suitable.* |
| ☒ | ☐ | For Bayesian analysis, information on the choice of priors and Markov chain Monte Carlo settings |
| ☒ | ☐ | For hierarchical and complex designs, identification of the appropriate level for tests and full reporting of outcomes |
| ☒ | ☐ | Estimates of effect sizes (e.g. Cohen's *d*, Pearson's *r*), indicating how they were calculated |

*Our web collection on statistics for biologists contains articles on many of the points above.*

## Software and code

Policy information about availability of computer code

| | |
|---|---|
| Data collection | Data were collected using instrument-associated acquisition software.<br>- Luminescence, fluorescence and absorbance measurements were acquired using the Microwin 2010 5.24 software associated with the Centro XS³ LB 960 luminometer, the perkin elmer 2030 manager 4.0 with the Victor X3 multilabel plate reader and Biotek ELx800 spectrophotometer.<br>- Immunoblot images were acquired using the multigauge 2.1.1 software associated with LAS-3000 and the imageQuant TL 10.2 with ImageQuant 800 imaging systems.<br>- Microscale thermophoresis data were acquired on a Monolith NT.115 Pico instrument using MO.Control 1.6.1 software.<br>- Atomic force microscopy images were acquired on a FastScan Bio microscope using NanoScope 9.4 software.<br>- Proteomics data were acquired using the Chromeleon 7 associated with the RSLC Ultimate 3000 system coupled to an Eclipse mass spectrometer,<br>- Sequencing data for ChIP-seq and Hi-C were generated on Illumina sequencing platforms<br>- qPCR data were acquired using MxPro 4.10 software. |
| Data analysis | - Statistical analyses were performed using GraphPad Prism 10.<br>- Proteomics data were processed using Proteome Discoverer 1.4, Mascot 2.6.0 and Progenesis 4.1 LC-MS/MS.<br>- Metabolomics data were processed using TraceFinder and MetaboAnalyst 4.0.<br>- Hi-C data were processed using Cutadapt 3.2, Bowtie2 2.4.2 and the OCHA pipeline in R (https://github.com/js2264/OHCA) .<br>- ChIP-seq reads were processed using Bowtie2 Galaxy Version 2.5.5+galaxy0; MACS2 callpeak Galaxy Version 2.2.9.1+galaxy0 |

- MST data were analysed using MO.Affinity Analysis 2.3.
- AFM images were processed using NanoScope Analysis 1.9, and DNA bend angles were measured using ImageJ 1.52a.

For manuscripts utilizing custom algorithms or software that are central to the research but not yet described in published literature, software must be made available to editors and reviewers. We strongly encourage code deposition in a community repository (e.g. GitHub). See the Nature Portfolio guidelines for submitting code & software for further information.

## Data

Policy information about availability of data

All manuscripts must include a data availability statement. This statement should provide the following information, where applicable:

- Accession codes, unique identifiers, or web links for publicly available datasets
- A description of any restrictions on data availability
- For clinical datasets or third party data, please ensure that the statement adheres to our policy

- The mass spectrometry proteomics data have been deposited with the ProteomeXchange Consortium via the PRIDE partner repository under the dataset identifier PXD071830. The identification and quantification of proteomics data has been deposited in Zenodo (DOI : 10.5281/zenodo.18209150).
- The metabolomics data have been deposited in Zenodo (DOI : 10.5281/zenodo.17786388). ChIP-Seq data have been submitted to NCBI under the BioProject accession PRJNA1372475. The pPromX-GFP sequence has been deposited in GenBank (under the accession number PX762416).
- Hi-C Data have been deposited the Sequence Read Archive (SRA) under the BioSample accessions SAMN54411906 and SAMN54411907.

## Research involving human participants, their data, or biological material

Policy information about studies with human participants or human data. See also policy information about sex, gender (identity/presentation), and sexual orientation and race, ethnicity and racism.

| | |
|---|---|
| Reporting on sex and gender | N/A |
| Reporting on race, ethnicity, or other socially relevant groupings | N/A |
| Population characteristics | N/A |
| Recruitment | N/A |
| Ethics oversight | Commercially obtained normal human serum was used as an anonymized biological reagent for in vitro complement-mediated killing assays. The study did not involve human research participants, human clinical data, identifiable human-derived samples, or collection of human material by the investigators. Therefore, ethics approval for human participant research and informed consent were not required. |

Note that full information on the approval of the study protocol must also be provided in the manuscript.

# Field-specific reporting

Please select the one below that is the best fit for your research. If you are not sure, read the appropriate sections before making your selection.

☒ Life sciences ☐ Behavioural & social sciences ☐ Ecological, evolutionary & environmental sciences

For a reference copy of the document with all sections, see nature.com/documents/nr-reporting-summary-flat.pdf

# Life sciences study design

All studies must disclose on these points even when the disclosure is negative.

| | |
|---|---|
| Sample size | No sample-size calculation was performed. Sample sizes were chosen based on prior experience with the assays, standard experimental practice for microbiological, biochemical and flea infection experiments, feasibility constraints for vector infection experiments, and the need to assess reproducibility across independent biological replicates. Sample sizes are reported in the corresponding figure legends and include independent biological replicates for in vitro assays, independent omics replicates, and individual fleas or flea cohorts for vector infection experiments, as appropriate. These sample sizes were considered sufficient because the main findings were supported by reproducible effects across independent experiments and, where applicable, by concordant results across complementary experimental approaches. |
| Data exclusions | No samples were excluded from the analysis. For AFM-based DNA bend-angle measurements, only DNA molecules that were fully visible and could be unambiguously traced were included, according to predefined image-quality criteria. |
| Replication | Experiments were performed using independent biological replicates, as indicated in the figure legends. In vitro phenotypes were reproduced across independent experiments, omics analyses were performed with multiple biological replicates, and flea infection or co-infection experiments were performed using independent flea cohorts. All replication attempts supporting the main conclusions were successful. |
| Randomization | Experimental groups were defined by the study design and corresponded to predefined bacterial genotypes, complementation constructs, growth temperatures, treatments or assay conditions. Formal random allocation was therefore not applicable for most in vitro experiments. |

Strains and conditions were processed in parallel under matched experimental conditions to limit batch effects. For flea infection experiments, fleas were randomly collected from the available colony population for each infection, and fully engorged fleas were randomly selected after the infectious blood meal for downstream analyses.

Blinding

Except for the compound screening assay, investigators were not blinded to group allocation during data collection and analysis because strain identity and treatment conditions were required for experimental setup, culture conditions, complementation strategies, infection design and downstream interpretation. For the compound screening assay, compounds were arrayed in plates by one experimenter and analysed by another experimenter, reducing direct operator bias during analysis. Potential bias was further limited by the use of predefined experimental conditions, objective quantitative readouts, including luminescence, fluorescence, absorbance, CFU enumeration, immunoblot quantification, sequencing-based analyses and instrument-based measurements, and replication across independent experiments.

# Reporting for specific materials, systems and methods

We require information from authors about some types of materials, experimental systems and methods used in many studies. Here, indicate whether each material, system or method listed is relevant to your study. If you are not sure if a list item applies to your research, read the appropriate section before selecting a response.

## Materials & experimental systems

| n/a | Involved in the study |
|---|---|
| ☐ | ☒ Antibodies |
| ☒ | ☐ Eukaryotic cell lines |
| ☒ | ☐ Palaeontology and archaeology |
| ☐ | ☒ Animals and other organisms |
| ☒ | ☐ Clinical data |
| ☐ | ☒ Dual use research of concern |
| ☒ | ☐ Plants |

## Methods

| n/a | Involved in the study |
|---|---|
| ☐ | ☒ ChIP-seq |
| ☒ | ☐ Flow cytometry |
| ☒ | ☐ MRI-based neuroimaging |

## Antibodies

Antibodies used

mouse anti-C1q, clone 242G3, monoclonal, Progen, cat. no. 610136PROGEN, dilution 1:250;
rabbit anti-MBL, polyclonal, Proteintech, cat. no. 24207-1-AP, dilution 1:1000;
mouse anti-Factor B, clone M13/12, monoclonal, Santa Cruz Biotechnology, cat. no. sc-47682, dilution 1:900;
mouse anti-C4α, clone C-2, monoclonal, Santa Cruz Biotechnology, cat. no. sc-271181, dilution 1:900;
mouse anti-C4β, clone D12, monoclonal, Santa Cruz Biotechnology, cat. no. sc-74524, dilution 1:900;
mouse anti-C3b, clone B-9, monoclonal, Santa Cruz Biotechnology, cat. no. sc-28294, dilution 1:10000;
mouse anti-C4bpα, clone D-5, monoclonal, Santa Cruz Biotechnology, cat. no. sc-398720, dilution 1:1000;
mouse anti-C4bpβ, clone E-1, monoclonal, Santa Cruz Biotechnology, cat. no. sc-514553, dilution 1:900;
mouse anti-FH, clone C18/3, monoclonal, Santa Cruz Biotechnology, cat. no. sc-47685, dilution 1:900;
mouse anti-vitronectin, clone D-8, monoclonal, Santa Cruz Biotechnology, cat. no. sc-74484, dilution 1:1000;
mouse anti-RNA polymerase, clone 4RA2, monoclonal, BioLegend, cat. no. 663104, dilution 1:5000 or 1:50000 depending on the assay;
rabbit anti-HmsH, polyclonal, custom antibody generated by GenScript, dilution 1:4000;
mouse anti-Caf1, clone YPF19, monoclonal, GeneTex, cat. no. GTX28275, dilution 1:2000;
mouse anti-Pla, clone Pla35, monoclonal, previously described in Simon et al., PLoS ONE 2013, dilution 1:1000000;
rabbit anti-LpxC, polyclonal, NBRP-E. coli at NIG, no clone applicable, dilution 1:5000;
rabbit anti-FLAG-Tag, polyclonal, Proteintech, cat. no. 20543-1-AP, no clone applicable, dilution 1:5000;
MonoRab anti-FLAG/DYKDDDDK magnetic beads, rabbit monoclonal, GenScript, cat. no. L00835-1, clone not specified by the supplier, amount 40 µL;
goat anti-mouse IgG-HRP secondary antibody, polyclonal, Invitrogen, cat. no. 31430, no clone applicable, dilution 1:5000;
goat anti-rabbit IgG-HRP secondary antibody, polyclonal, Invitrogen, cat. no. 31460, no clone applicable, dilution 1:4000.

Validation

Antibodies were used for western blotting, except the MonoRab anti-FLAG magnetic beads, which were used for FLAG-based protein purification. Antibody use for the relevant targets and applications was supported by manufacturer information, previous published use, and/or validation in this manuscript. Previous-use references include Heinz et al., 1984 for C1q; Alberts et al., 2020 for MBL; Wang et al., 2020 for Factor B; Feng et al., 2021 for C4α; Moliva et al., 2014 for C4β; Zhao et al., 2019 for C3b; Soares Martins et al., 2022 for C4BPα; Blom et al., 1999 for C4BPβ; Moore et al., 2010 for factor H; Muerza-Cascante et al., 2017 for vitronectin; Sanderlin et al., 2022 for RNA polymerase α; Rosales-Mendoza et al., 2010 for Caf1; Simon et al., 2013 for Pla; Li et al., 2024 for FLAG-tag detection; He et al., 2024 for MonoRab anti-FLAG magnetic beads; and the NBRP-E. coli antibody collection for LpxC. In this study, specificity was further supported by detection of bands at the expected molecular weight. The custom rabbit anti-HmsH antibody was validated in this manuscript by detection of a band at the expected molecular weight and by loss or strong reduction of this band in an hmsH mutant control. Additional signal changes observed in strains affecting hmsHFRS regulation were consistent with the expected biology. The corresponding uncropped blot is provided in the Source Data.

# Animals and other research organisms

Policy information about studies involving animals; ARRIVE guidelines recommended for reporting animal research, and Sex and Gender in Research

| | |
|---|---|
| Laboratory animals | Mouse, NMRI strain, 2-5 days old. Xenopsylla cheopis adult fleas from a laboratory-maintained colony; exact age not recorded. |
| Wild animals | This study did not involve wild animals |
| Reporting on sex | Sex was considered for flea infection experiments where relevant. Colonization kinetics and co-infection assays were performed using female Xenopsylla cheopis fleas to obtain sufficiently large and homogeneous cohorts, following an established protocol. Mixed-sex cohorts were used for blockage monitoring when appropriate. No sex-based comparative analysis was performed because the study was not designed to assess sex-specific effects. |
| Field-collected samples | The study did not involved samples collected from the field |
| Ethics oversight | All animal procedures were approved by the local Animal Care and Use Committee (Comité d'éthique en expérimentation animale n° 75; reference: 2015102609372221 and 2022020316019750) and notified to the relevant French authorities, in accordance with current national legislation (Government Decree No. 2017-1411; Articles R.214-87 to R.214-126). |

Note that full information on the approval of the study protocol must also be provided in the manuscript.

# Dual use research of concern

Policy information about dual use research of concern

## Hazards

Could the accidental, deliberate or reckless misuse of agents or technologies generated in the work, or the application of information presented in the manuscript, pose a threat to:

| No | Yes | |
|---|---|---|
| ☒ | ☐ | Public health |
| ☒ | ☐ | National security |
| ☒ | ☐ | Crops and/or livestock |
| ☒ | ☐ | Ecosystems |
| ☒ | ☐ | Any other significant area |

## Experiments of concern

Does the work involve any of these experiments of concern:

| No | Yes | |
|---|---|---|
| ☒ | ☐ | Demonstrate how to render a vaccine ineffective |
| ☒ | ☐ | Confer resistance to therapeutically useful antibiotics or antiviral agents |
| ☒ | ☐ | Enhance the virulence of a pathogen or render a nonpathogen virulent |
| ☒ | ☐ | Increase transmissibility of a pathogen |
| ☒ | ☐ | Alter the host range of a pathogen |
| ☒ | ☐ | Enable evasion of diagnostic/detection modalities |
| ☒ | ☐ | Enable the weaponization of a biological agent or toxin |
| ☒ | ☐ | Any other potentially harmful combination of experiments and agents |

## Plants

Seed stocks

*Report on the source of all seed stocks or other plant material used. If applicable, state the seed stock centre and catalogue number. If plant specimens were collected from the field, describe the collection location, date and sampling procedures.*

Novel plant genotypes

*Describe the methods by which all novel plant genotypes were produced. This includes those generated by transgenic approaches, gene editing, chemical/radiation-based mutagenesis and hybridization. For transgenic lines, describe the transformation method, the number of independent lines analyzed and the generation upon which experiments were performed. For gene-edited lines, describe the editor used, the endogenous sequence targeted for editing, the targeting guide RNA sequence (if applicable) and how the editor was applied.*

Authentication

*Describe any authentication procedures for each seed stock used or novel genotype generated. Describe any experiments used to assess the effect of a mutation and, where applicable, how potential secondary effects (e.g. second site T-DNA insertions, mosiacism, off-target gene editing) were examined.*

## ChIP-seq

### Data deposition

☒ Confirm that both raw and final processed data have been deposited in a public database such as GEO.

☒ Confirm that you have deposited or provided access to graph files (e.g. BED files) for the called peaks.

Data access links
*May remain private before publication.*

https://dataview.ncbi.nlm.nih.gov/object/PRJNA1372475

Files in database submission

FLAG-MaoP whole-cell extract, replicate 1: SRR36267233 ; FLAG-MaoP whole-cell extract, replicate 2: SRR36267232 ; FLAG-MaoP whole-cell extract, replicate 3: SRR36267231 ; MaoP whole-cell extract, replicate 1: SRR36267236 ; MaoP whole-cell extract, replicate 2: SRR36267235 ; MaoP whole-cell extract, replicate 3: SRR36267234

Genome browser session
(e.g. UCSC)

no longer applicable

### Methodology

Replicates

ChIP-seq was performed using three independent biological replicate cultures of the MaoP-FLAG strain and three independent biological replicate cultures of the corresponding untagged parental control strain. The six ChIP DNA samples were processed in parallel for library preparation and sequencing, and all corresponding libraries passed platform quality controls for DNA quantity, fragment-size profile, library profile and sequencing depth. Reads from biological replicates were pooled for peak calling to reduce the influence of any single sample. Candidate MaoP-bound regions identified by ChIP-seq were subsequently evaluated experimentally in the manuscript using independent approaches.

Sequencing depth

SRR36267236 - 16631270 reads, coverage: 496
SRR36267235 - 18993996 reads, coverage: 562
SRR36267234 - 17716785 reads, coverage: 530
SRR36267233 - 16980579 reads, coverage: 507
SRR36267232 - 15263123 reads, coverage: 461
SRR36267231 - 10058328 reads, coverage: 301

Antibodies

rabbit anti-MonoRab anti-FLAG magnetic Beads ;GenScript (L00835-1)

Peak calling parameters

Reads were mapped to the Yersinia pestis Kim10 genome (chromosome NC_004088.1 and plasmid NC_004838.1) using Bowtie 2 with default parameters. Treatment and control BAM files were pooled prior to peak calling. Peaks were identified using MACS2 Callpeak with a q-value cutoff of 0.05 (FDR ≤ 5%), lower and upper mfold bounds of 5 and 50, respectively, and a bandwidth of 300 bp for fragment size estimation. All other parameters were left at their default settings.

Data quality

MACS2 peak calling (q ≤ 0.05) identified 855 significant peaks (842 chromosomal and 13 plasmid-associated). All peaks passed the FDR ≤ 5% threshold, while none showed >5-fold enrichment (fold enrichment range: 1.05–1.95).

Software

Bowtie2 Galaxy Version 2.5.5+galaxy0; MACS2 callpeak Galaxy Version 2.2.9.1+galaxy0
