# Supplementary material for: A dual-host regulatory hub integrating biofilm formation and innate immunity resistance
Source: Nat Commun. 2026 Sep 1;17:10385. doi: 10.1038/s41467-026-77307-4 (PMC13627719; doi:10.1038/s41467-026-77307-4)

Figure 1E

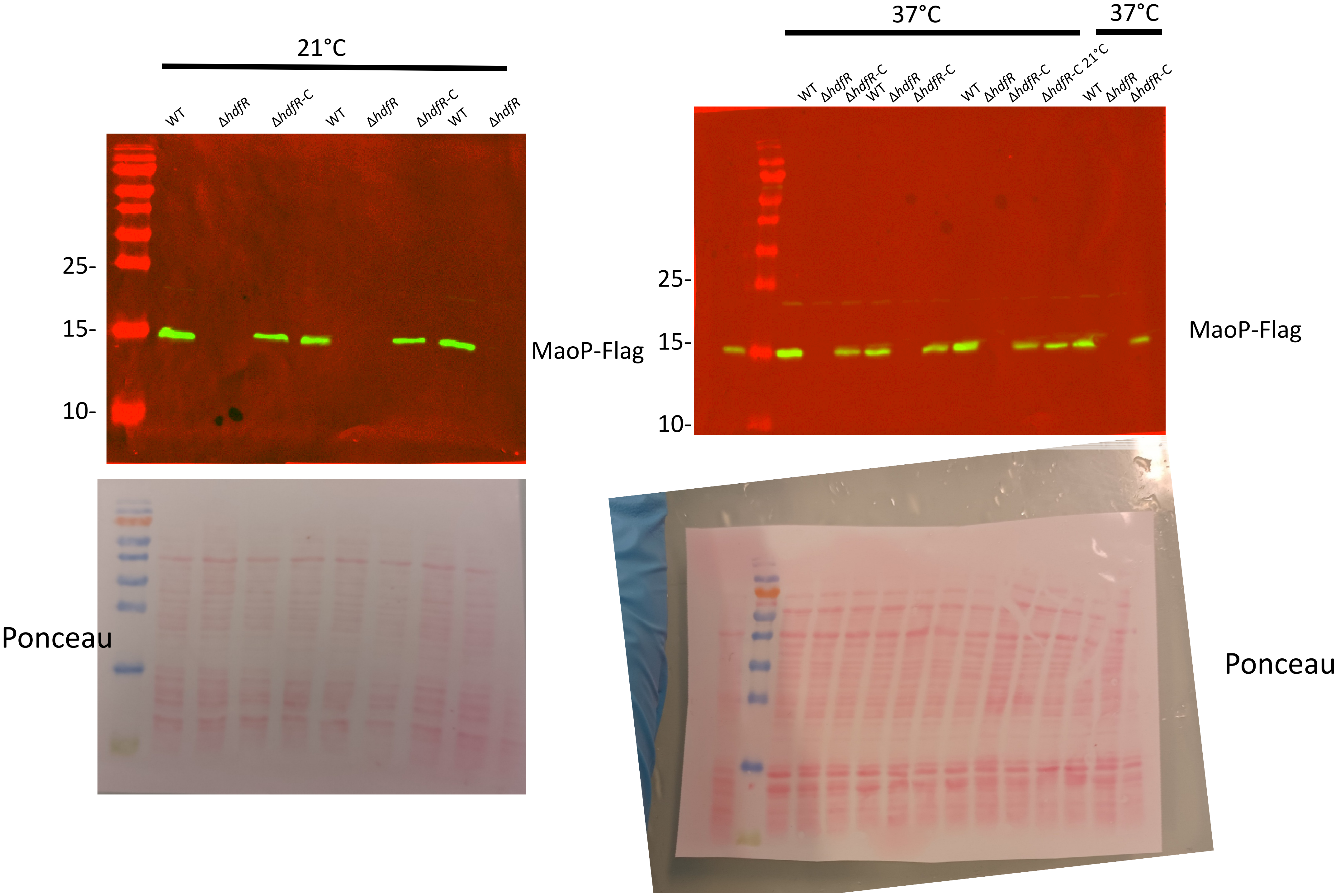

21°C
WT
ΔhdfR
ΔhdfR-C
WT
ΔhdfR
ΔhdfR-C
WT
ΔhdfR
25-
15-
10-
MaoP-Flag
Ponceau
37°C
37°C
WT
ΔhdfR
ΔhdfR-C
WT
ΔhdfR
ΔhdfR-C
WT
ΔhdfR
ΔhdfR-C
ΔhdfR-C 21°C
WT
ΔhdfR
ΔhdfR-C
25-
15-
10-
MaoP-Flag
Ponceau

Figure 2C

WB hmsH
hdfR MaoP et comp e

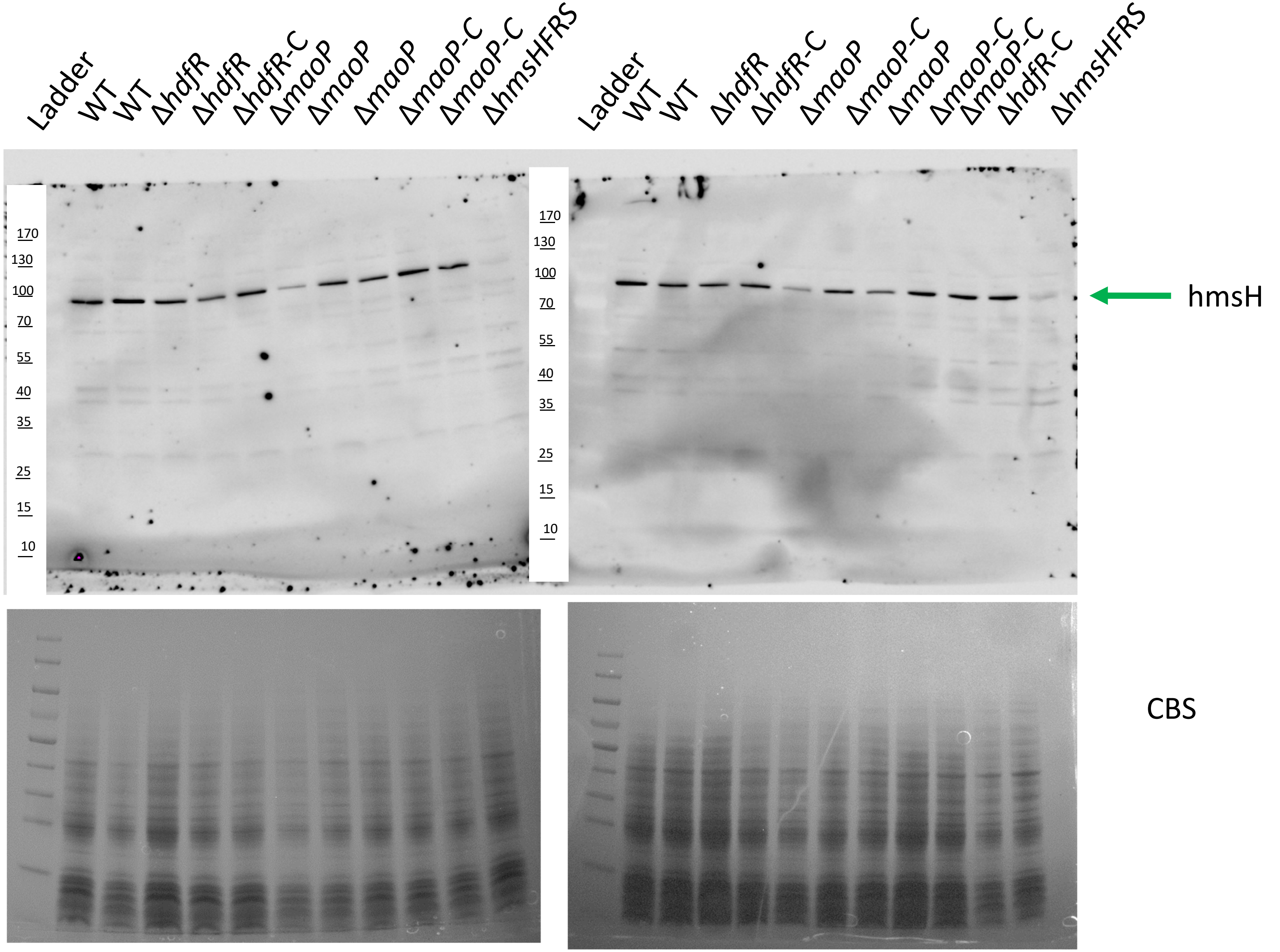

Figure 2C

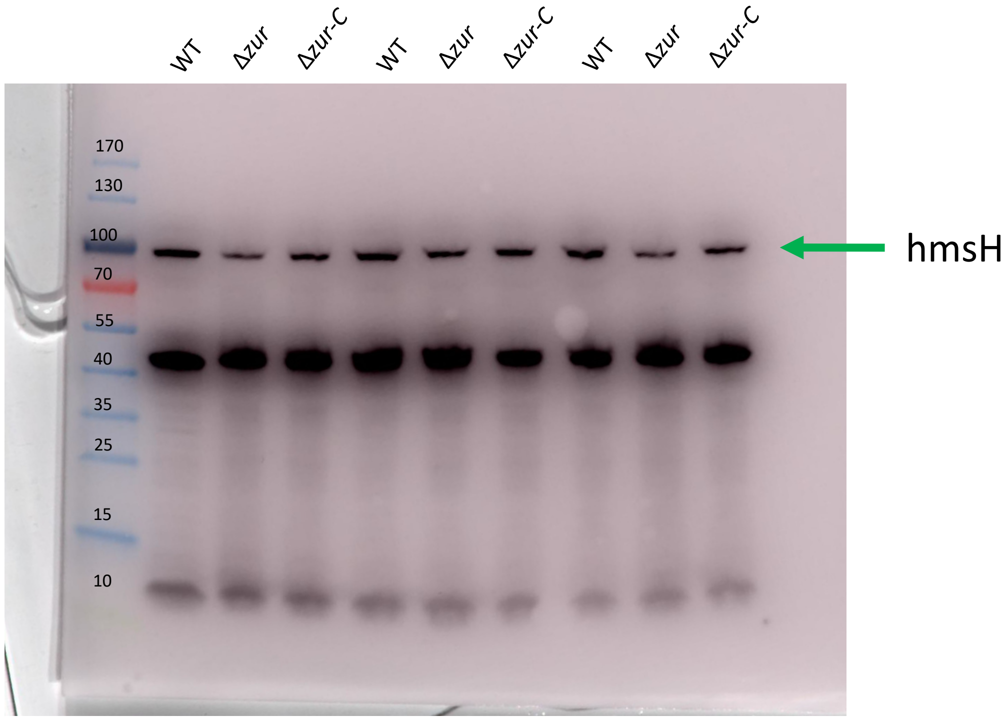
WT
Δzur
Δzur-C
WT
Δzur
Δzur-C
WT
Δzur
Δzur-C
170
130
100
70
55
40
35
25
15
10
hmsH

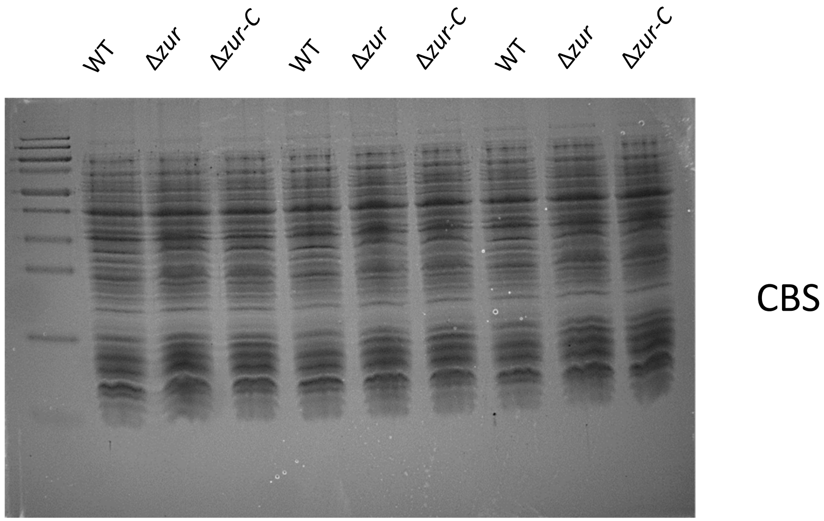
WT
Δzur
Δzur-C
WT
Δzur
Δzur-C
WT
Δzur
Δzur-C
CBS

Figure 2C

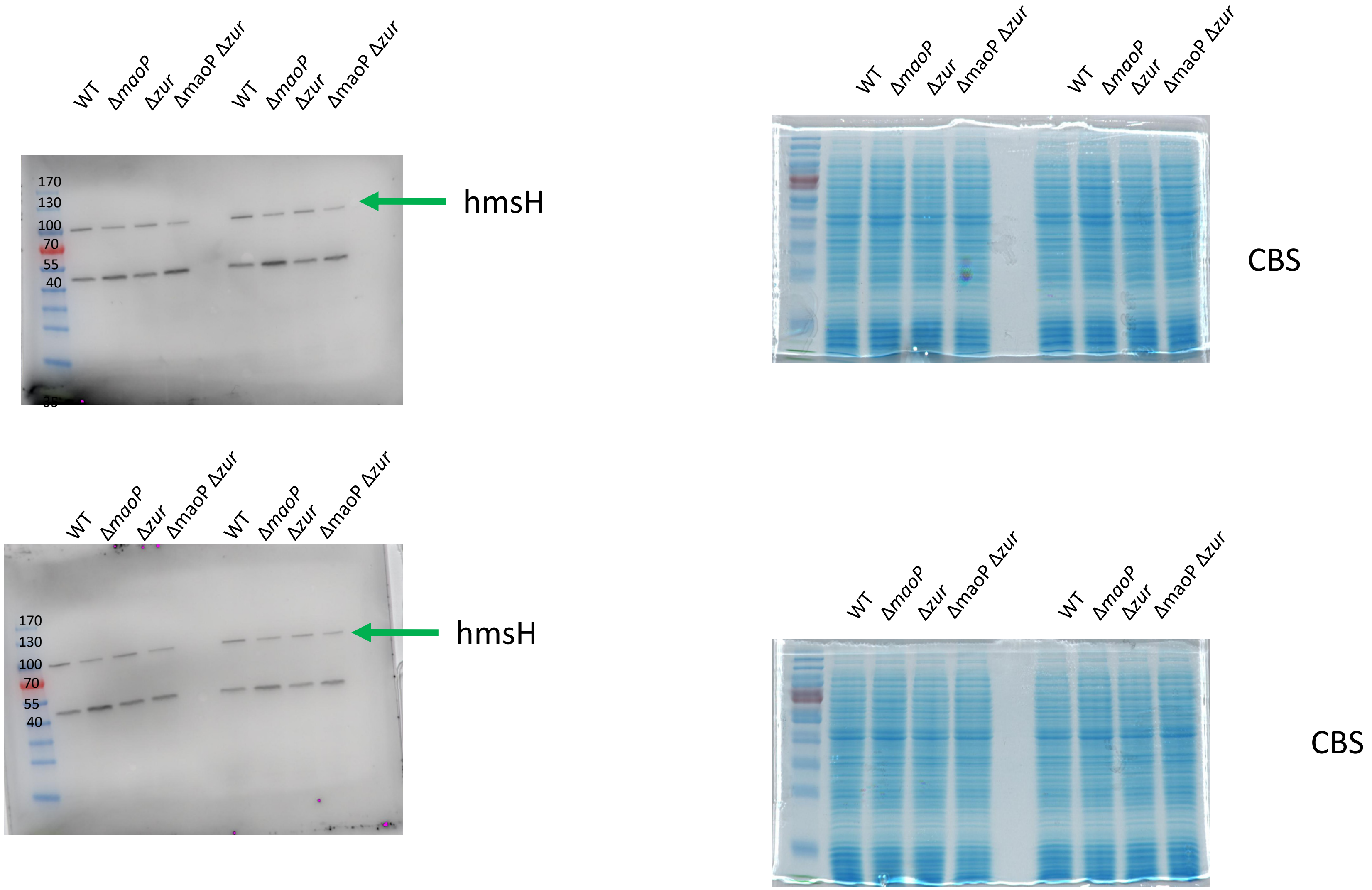
WT
ΔmaoP
Δzur
ΔmaoP Δzur
WT
ΔmaoP
Δzur
ΔmaoP Δzur
170
130
100
70
55
40
hmsH
WT
ΔmaoP
Δzur
ΔmaoP Δzur
WT
ΔmaoP
Δzur
ΔmaoP Δzur
CBS
WT
ΔmaoP
Δzur
ΔmaoP Δzur
WT
ΔmaoP
Δzur
ΔmaoP Δzur
170
130
100
70
55
40
hmsH
WT
ΔmaoP
Δzur
ΔmaoP Δzur
WT
ΔmaoP
Δzur
ΔmaoP Δzur
CBS

Figure 2C

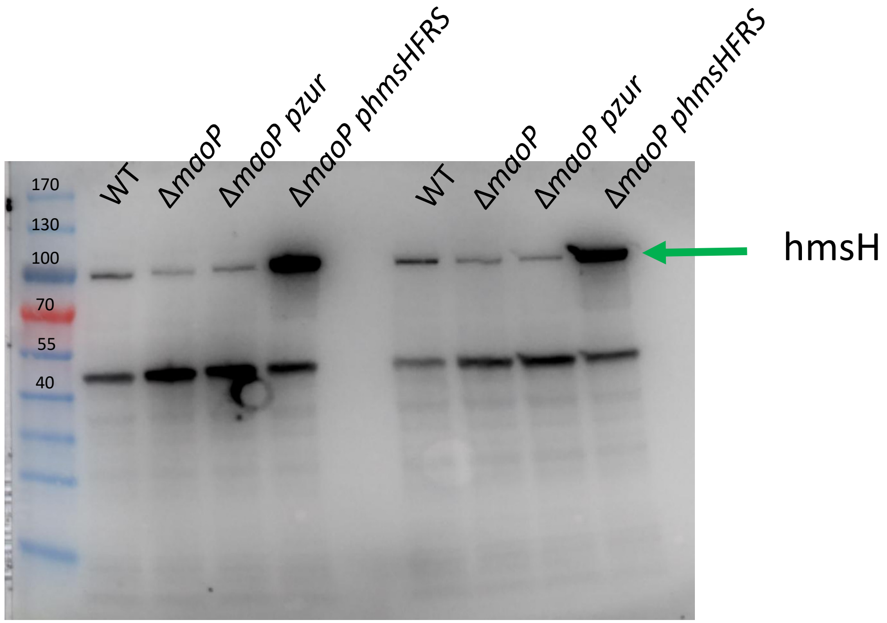
170
130
100
70
55
40
WT
ΔmaoP
ΔmaoP pzur
ΔmaoP phmsHFRS
WT
ΔmaoP
ΔmaoP pzur
ΔmaoP phmsHFRS
hmsH

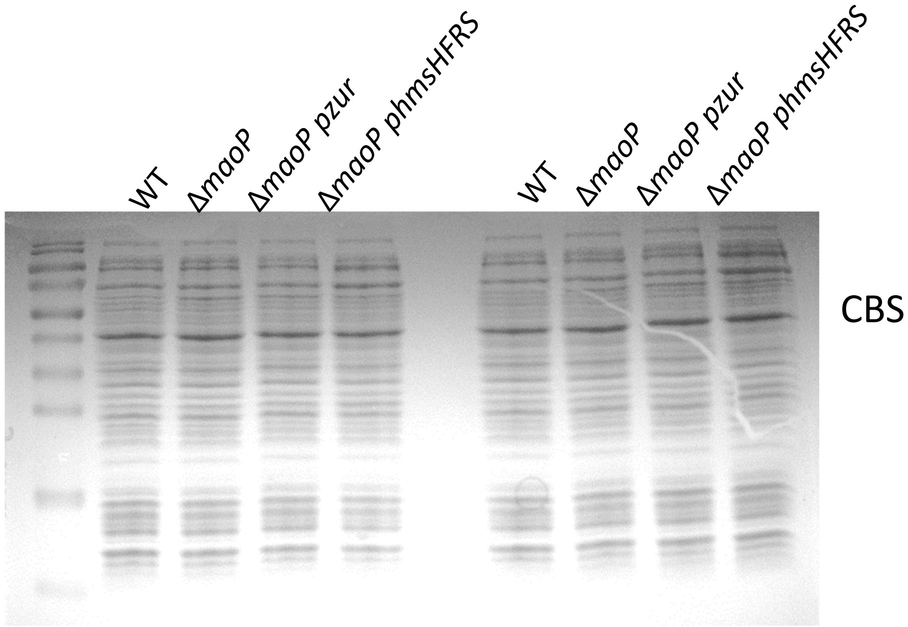
WT
ΔmaoP
ΔmaoP pzur
ΔmaoP phmsHFRS
WT
ΔmaoP
ΔmaoP pzur
ΔmaoP phmsHFRS
CBS

Figure 2D, Supplementary Fig. 4f

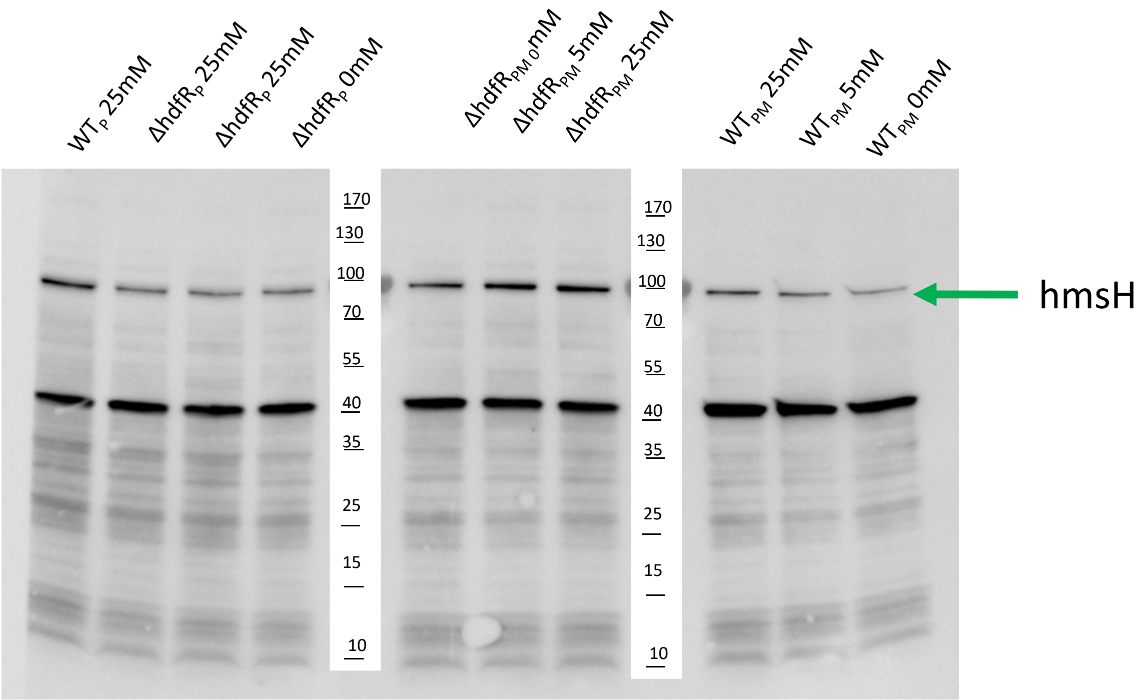

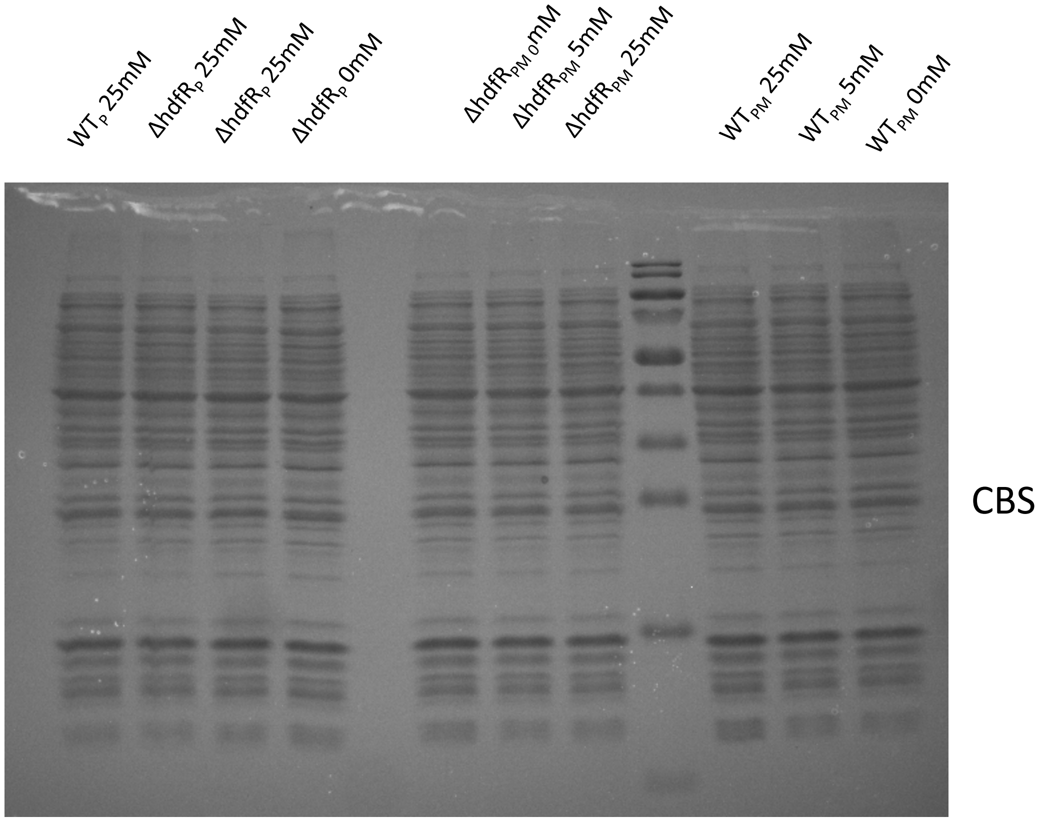

Figure 2D, Supplementary Fig. 4f

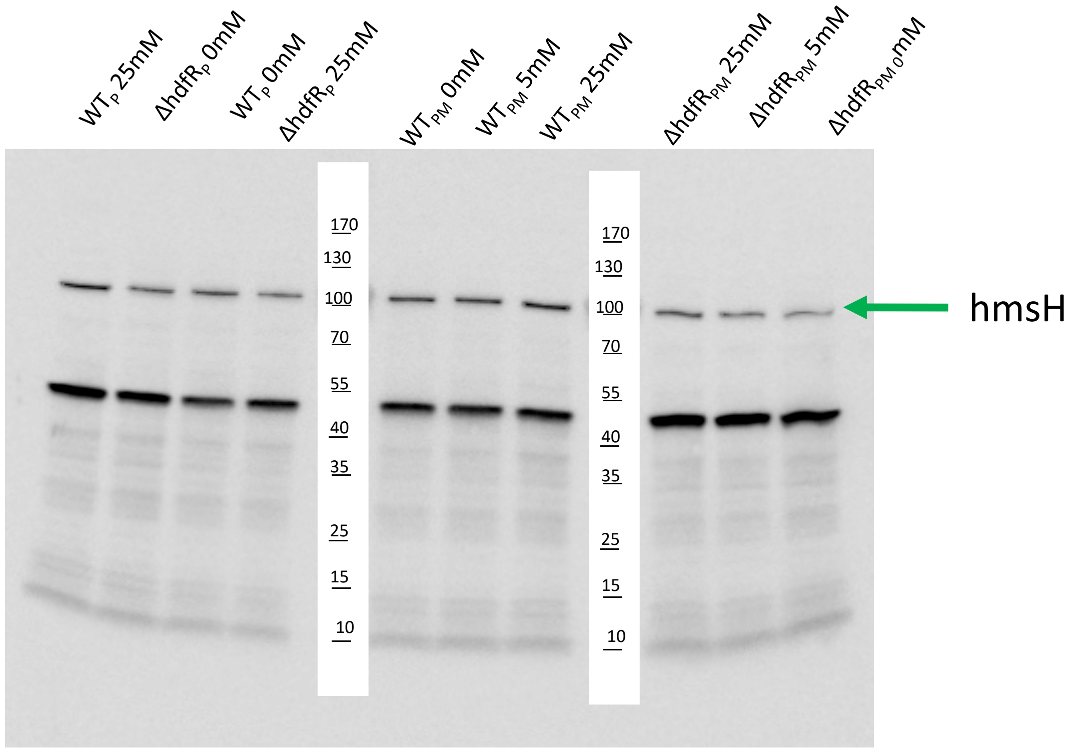

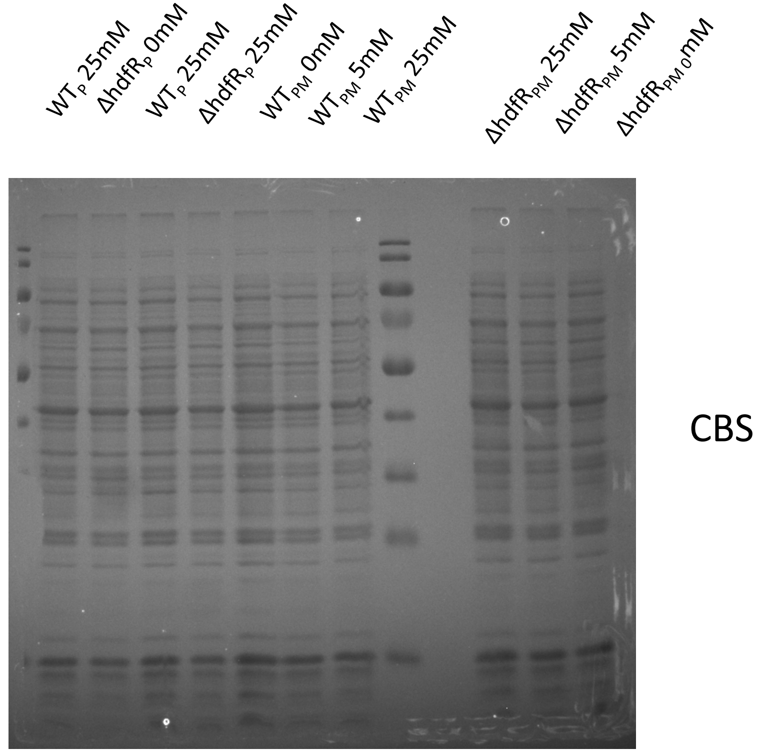

Figure 2D, Supplementary Fig. 4f

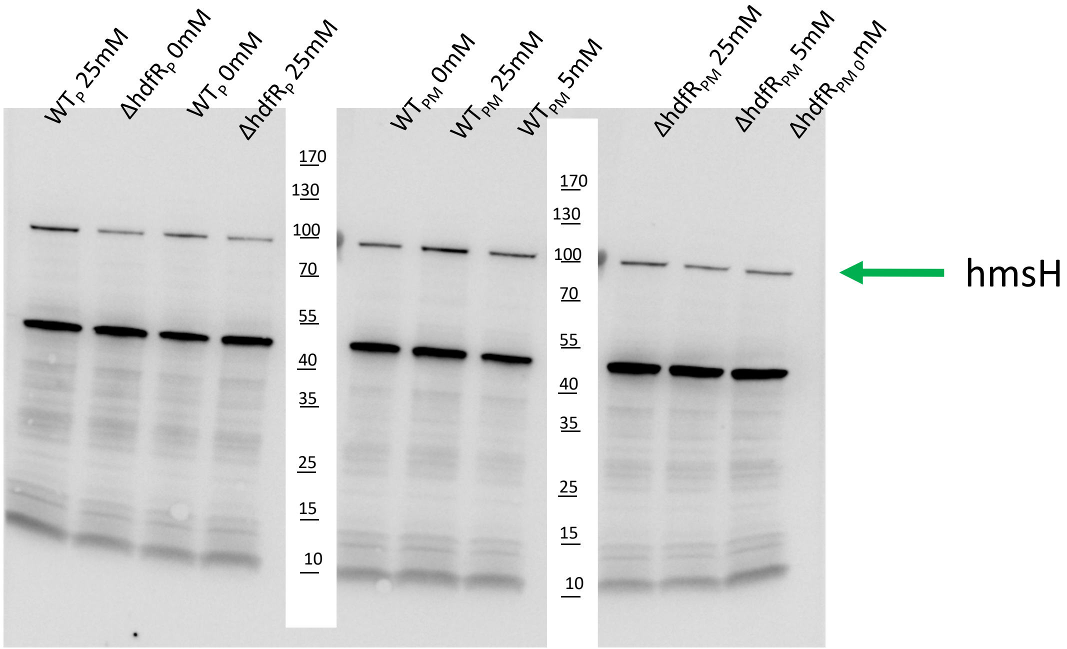

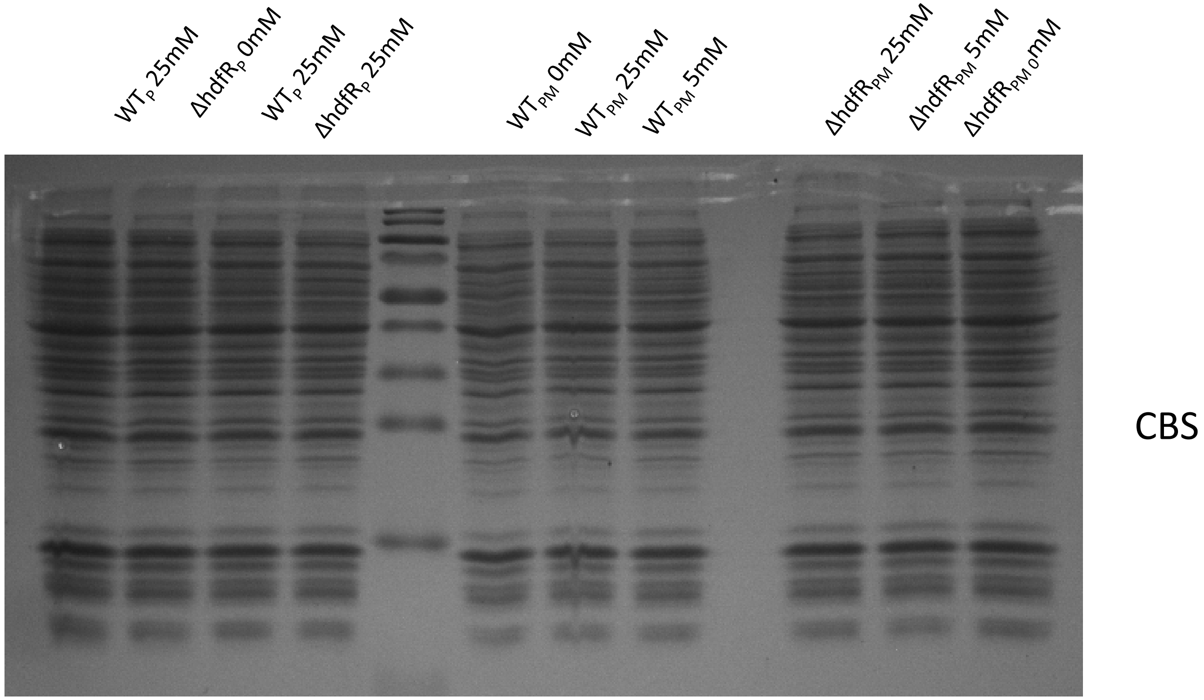

Figure 2D, Supplementary Fig. 4f

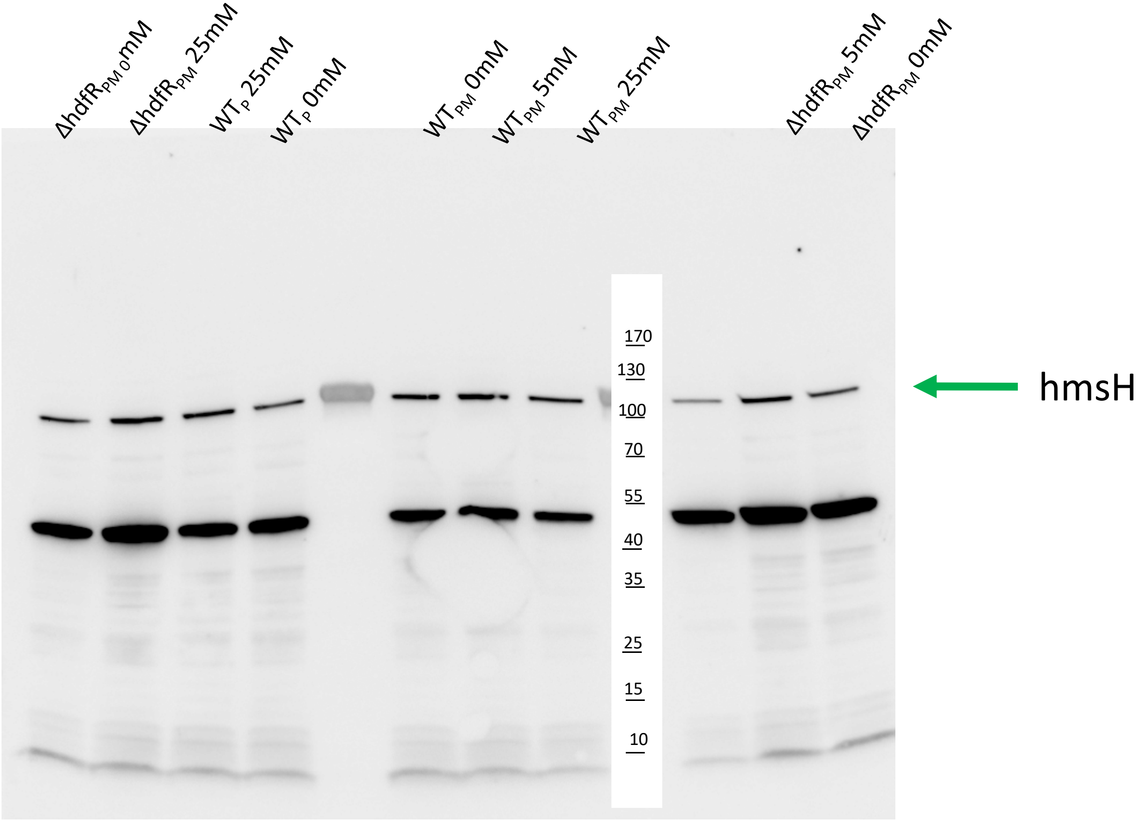

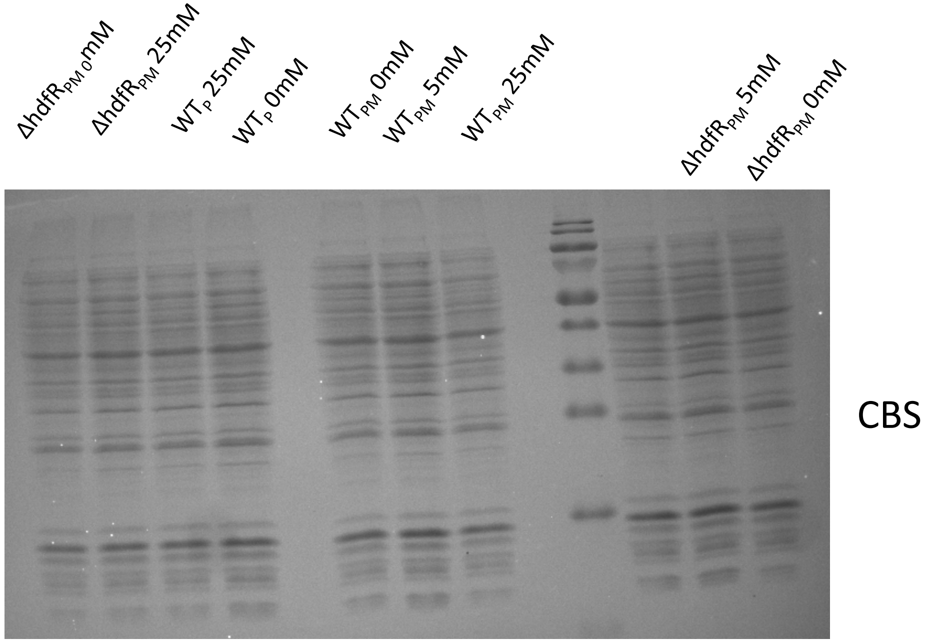

Figure 3b

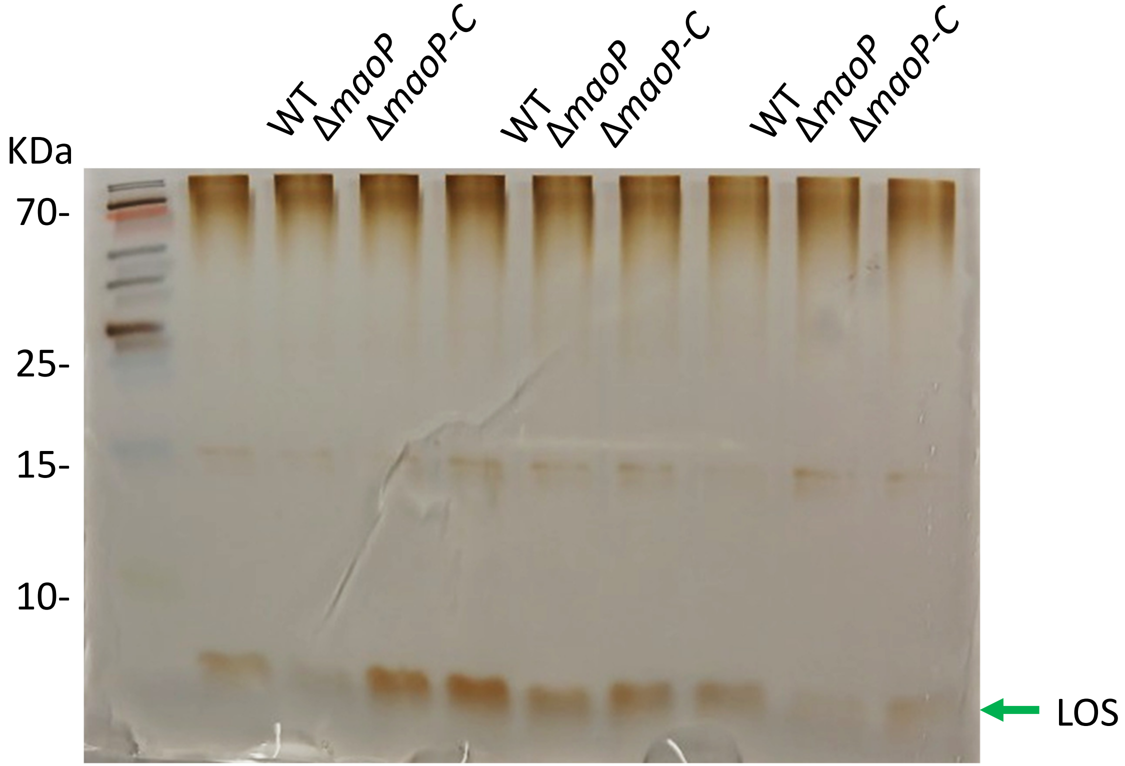
WT
ΔmaoP
ΔmaoP-C
WT
ΔmaoP
ΔmaoP-C
WT
ΔmaoP
ΔmaoP-C
KDa
70-
25-
15-
10-
LOS

Figure 3g

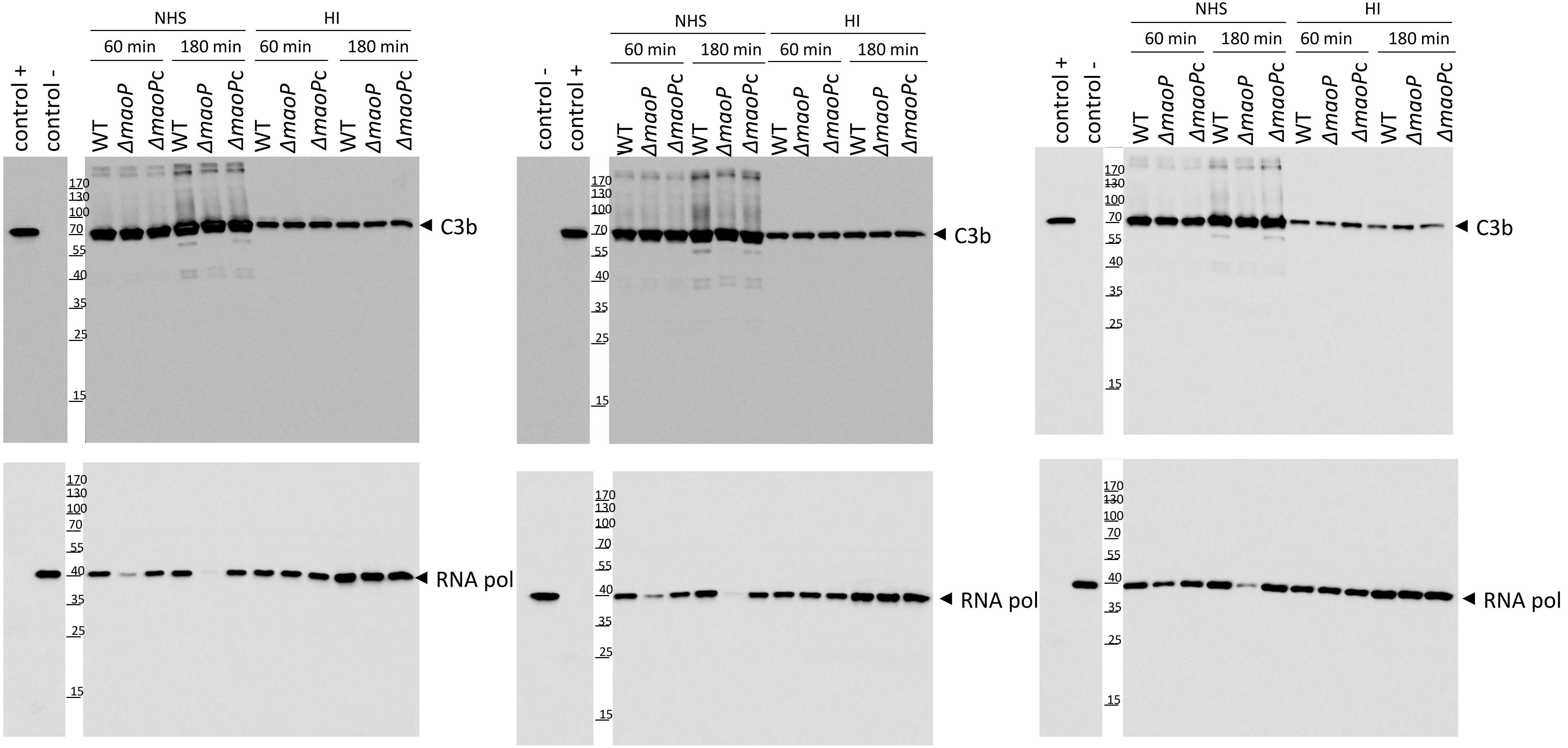

NHS
HI
60 min
180 min
60 min
180 min
control +
control -
WT
ΔmaoP
ΔmaoPc
170
130
100
70
55
40
35
25
15
C3b
RNA pol
NHS
HI
60 min
180 min
60 min
180 min
control -
control +
WT
ΔmaoP
ΔmaoPc
C3b
RNA pol
NHS
HI
60 min
180 min
60 min
180 min
control +
control -
WT
ΔmaoP
ΔmaoPc
C3b
RNA pol

Figure 3g

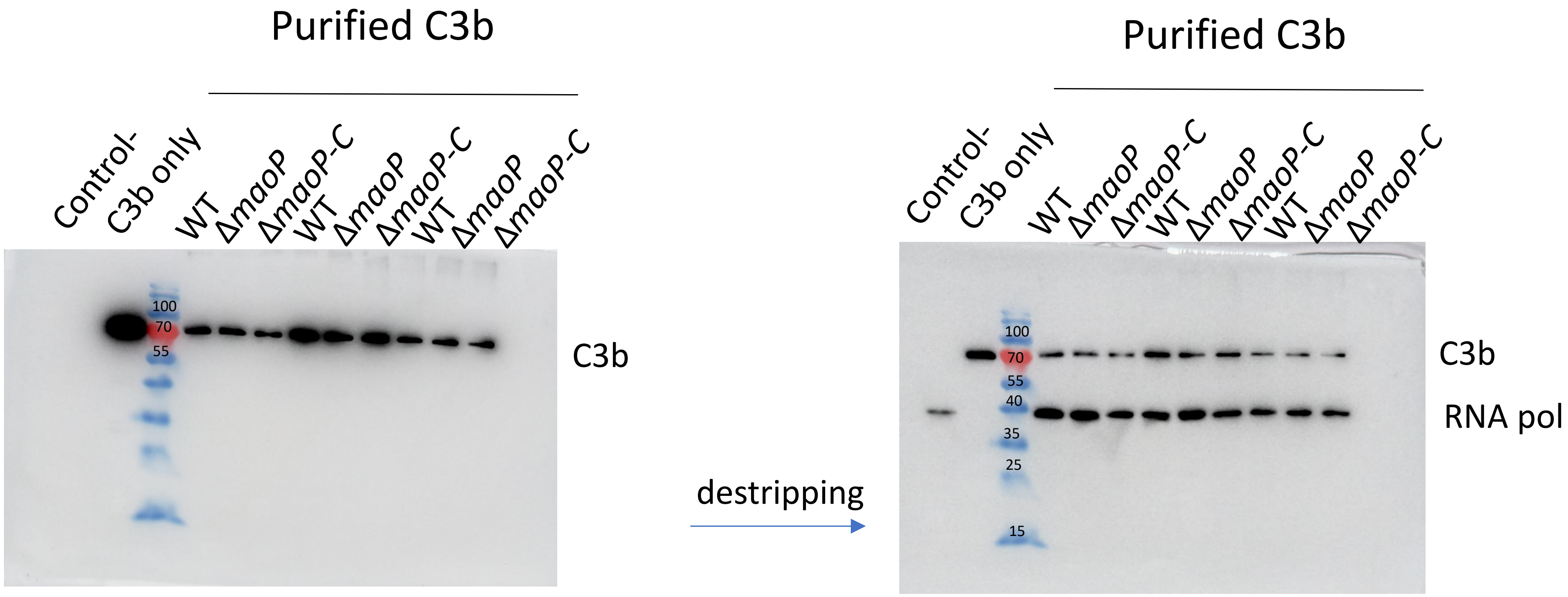
Purified C3b
Control-
C3b only
WT
ΔmaoP
ΔmaoP-C
WT
ΔmaoP
ΔmaoP-C
WT
ΔmaoP
ΔmaoP-C
100
70
55
C3b
destripping
Purified C3b
Control-
C3b only
WT
ΔmaoP
ΔmaoP-C
WT
ΔmaoP
ΔmaoP-C
WT
ΔmaoP
ΔmaoP-C
100
70
55
40
35
25
15
C3b
RNA pol

Figure 3h

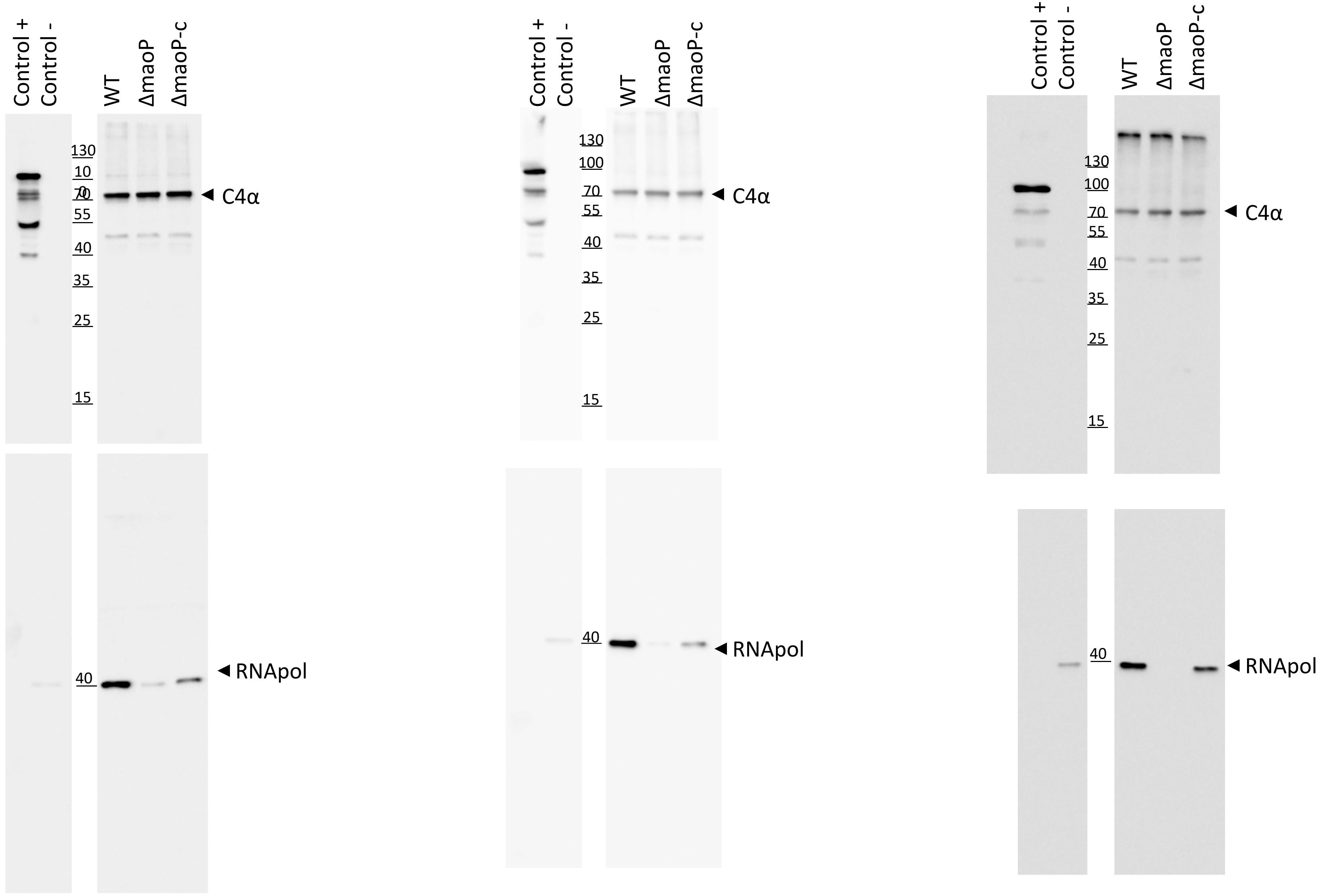

Control +
Control -
WT
ΔmaoP
ΔmaoP-c
130
10
70
55
40
35
25
15
C4α
40
RNApol
Control +
Control -
WT
ΔmaoP
ΔmaoP-c
130
100
70
55
40
35
25
15
C4α
40
RNApol
Control +
Control -
WT
ΔmaoP
ΔmaoP-c
130
100
70
55
40
35
25
15
C4α
40
RNApol

Figure 3h

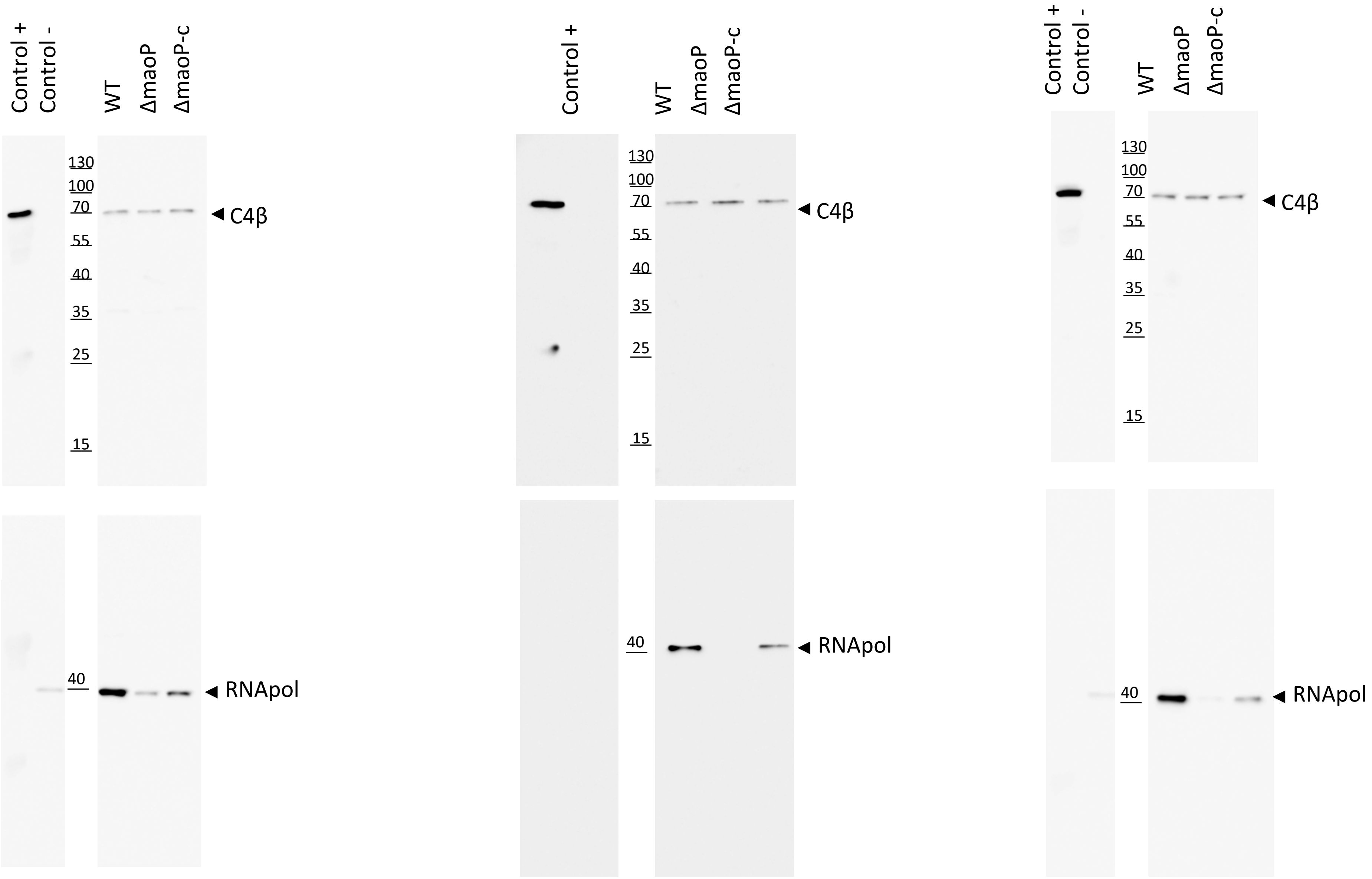

Control +
Control -
WT
ΔmaoP
ΔmaoP-c
130
100
70
55
40
35
25
15
C4β
40
RNApol
Control +
WT
ΔmaoP
ΔmaoP-c
130
100
70
55
40
35
25
15
C4β
40
RNApol
Control +
Control -
WT
ΔmaoP
ΔmaoP-c
130
100
70
55
40
35
25
15
C4β
40
RNApol

Figure 3h

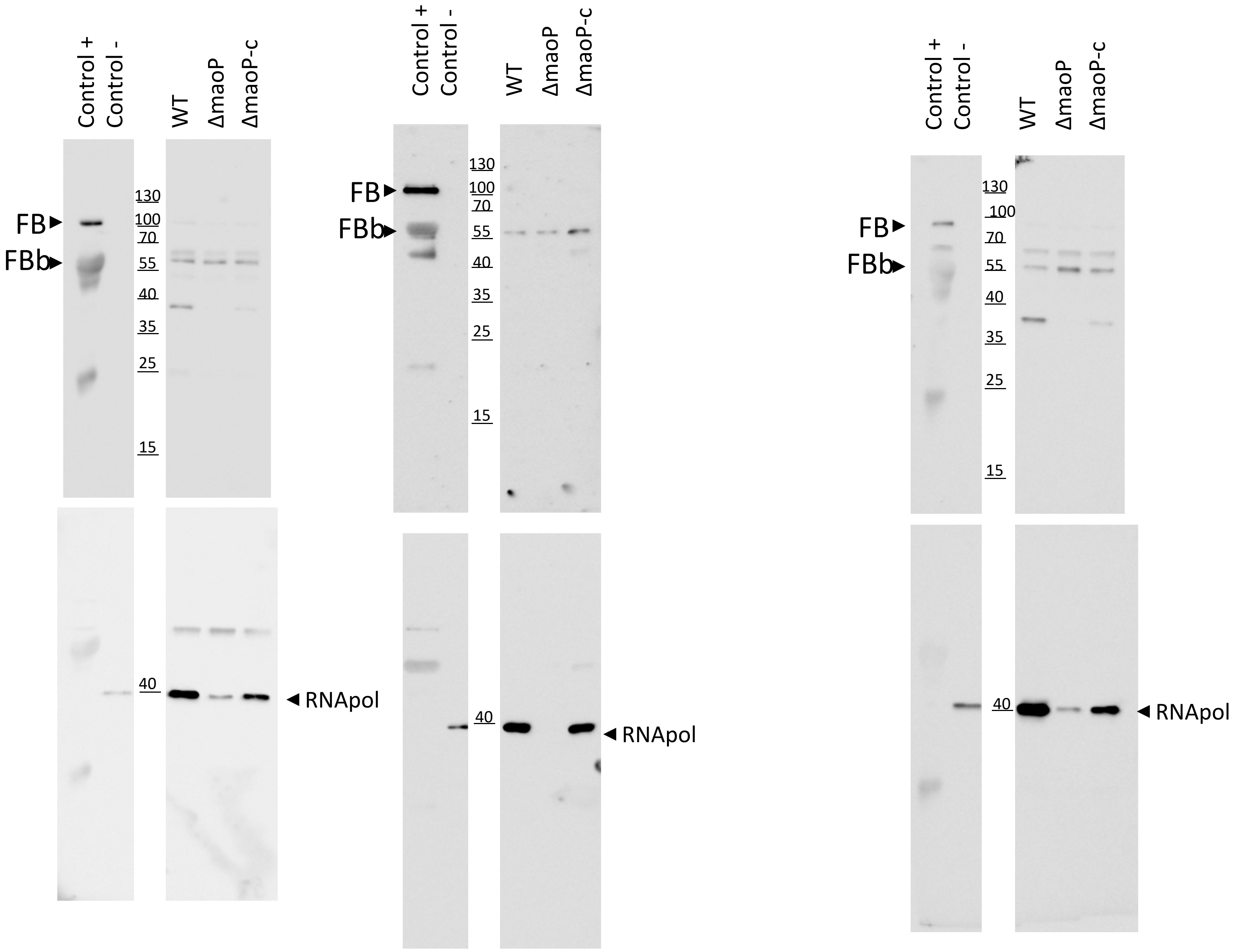
Control +
Control -
WT
ΔmaoP
ΔmaoP-c
FB
FBb
130
100
70
55
40
35
25
15
40
RNApol

Figure 3i

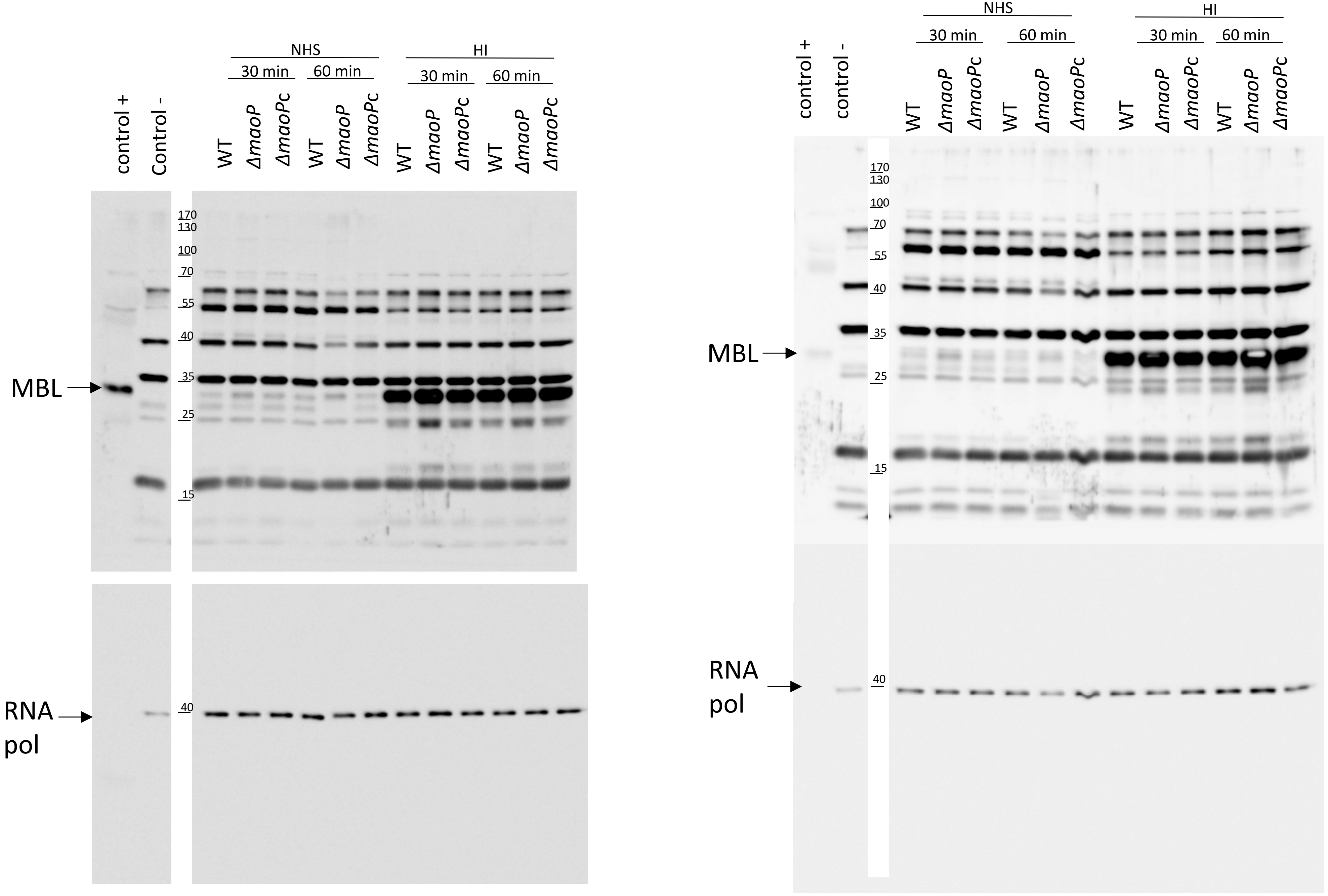
NHS
HI
30 min
60 min
control +
Control -
control -
WT
ΔmaoP
ΔmaoPc
170
130
100
70
55
40
35
25
15
MBL
RNA pol

Figure 3i

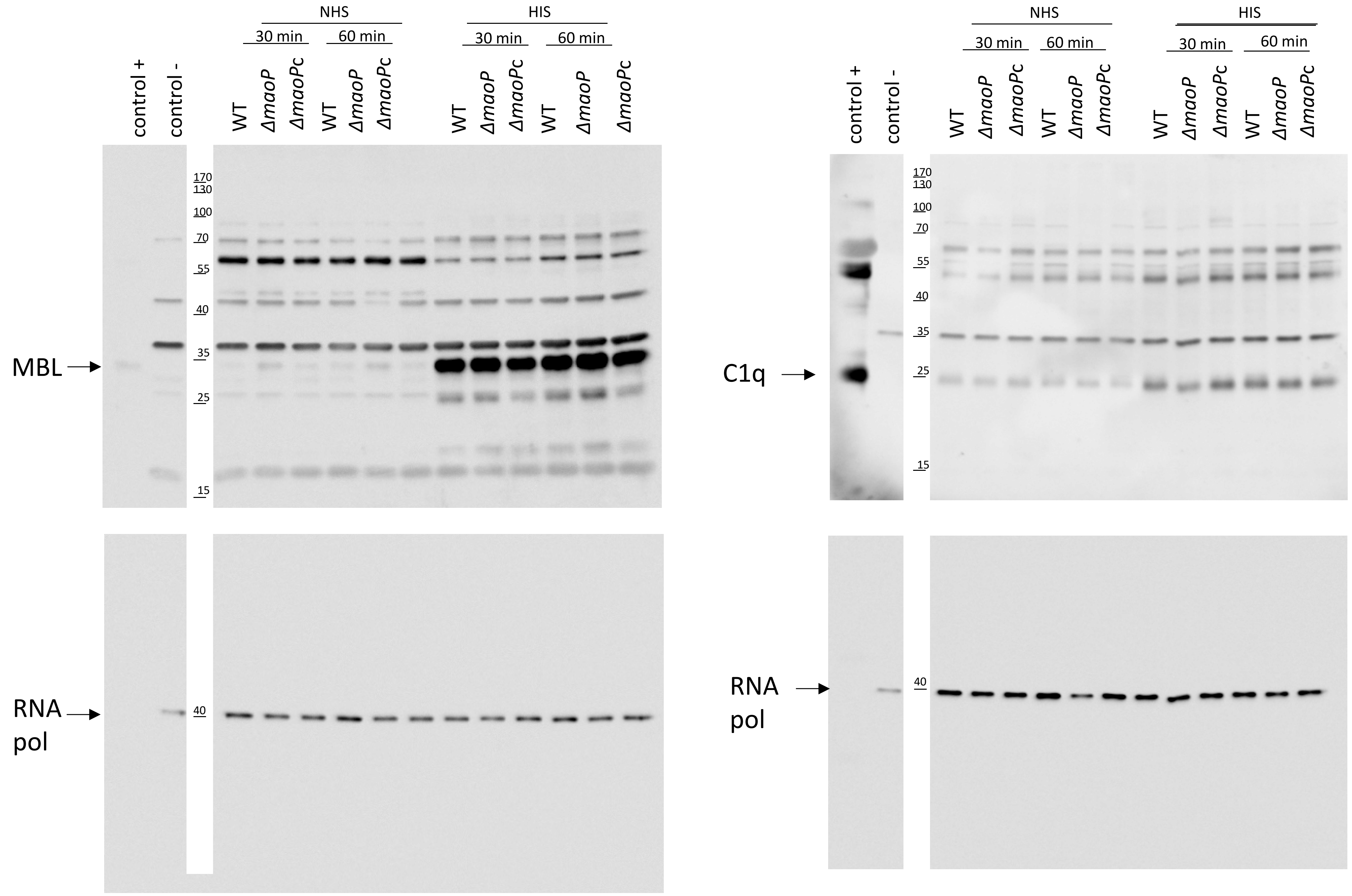
NHS
HIS
30 min
60 min
control +
control -
WT
ΔmaoP
ΔmaoPc
170
130
100
70
55
40
35
25
15
MBL
RNA pol
C1q

Figure 3i

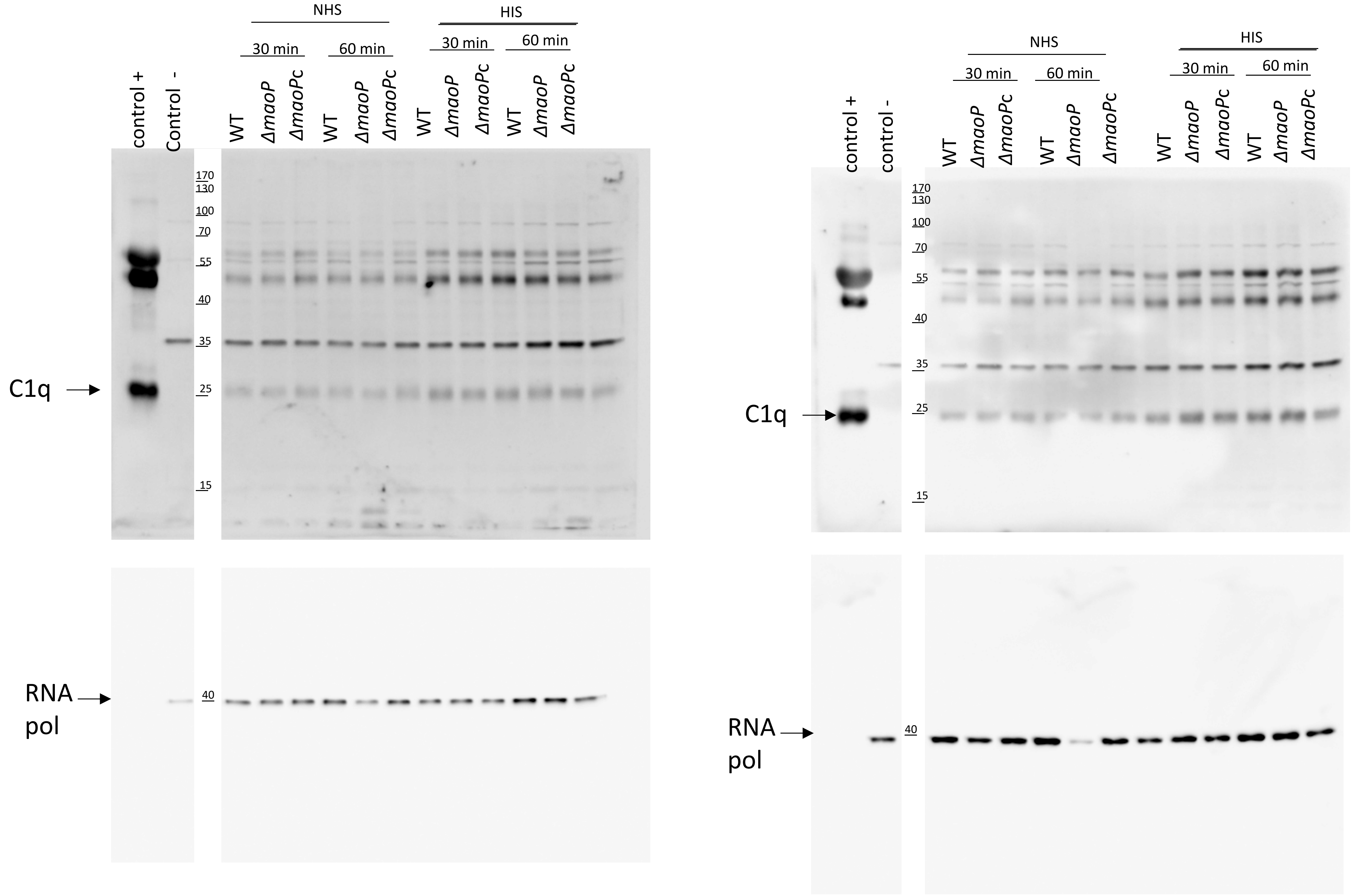
NHS
HIS
30 min
60 min
control +
Control -
control -
WT
ΔmaoP
ΔmaoPc
170
130
100
70
55
40
35
25
15
C1q
RNA pol

Supplementary Fig. 5f

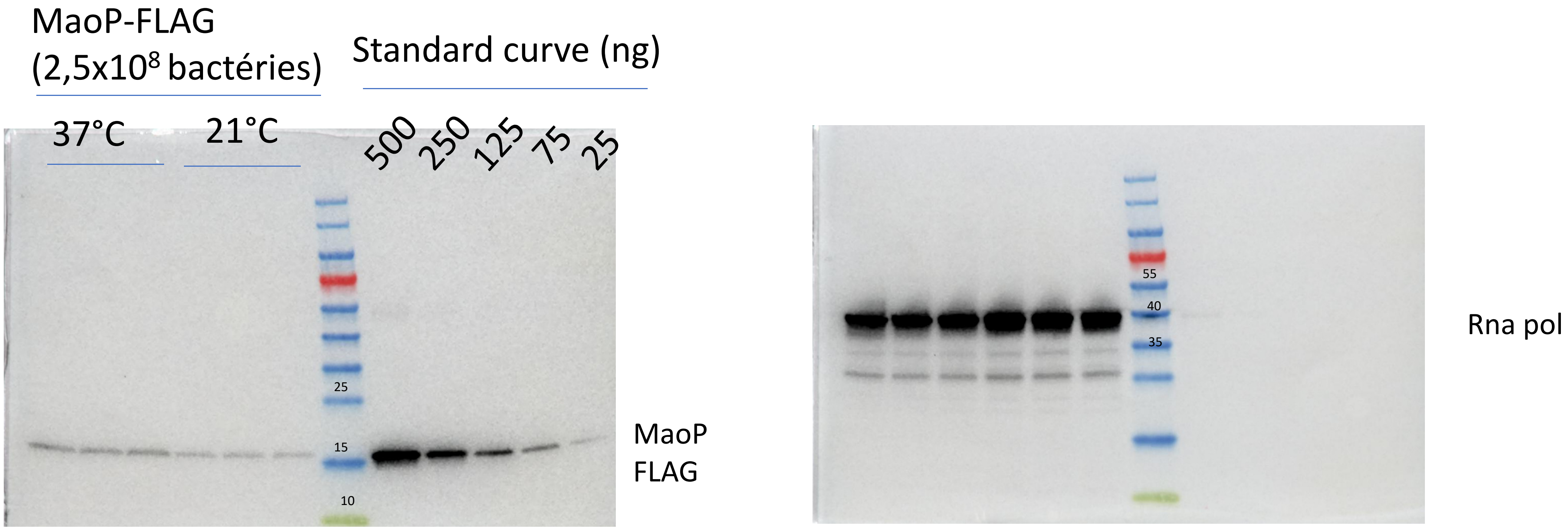

Supplementary Fig. 5g

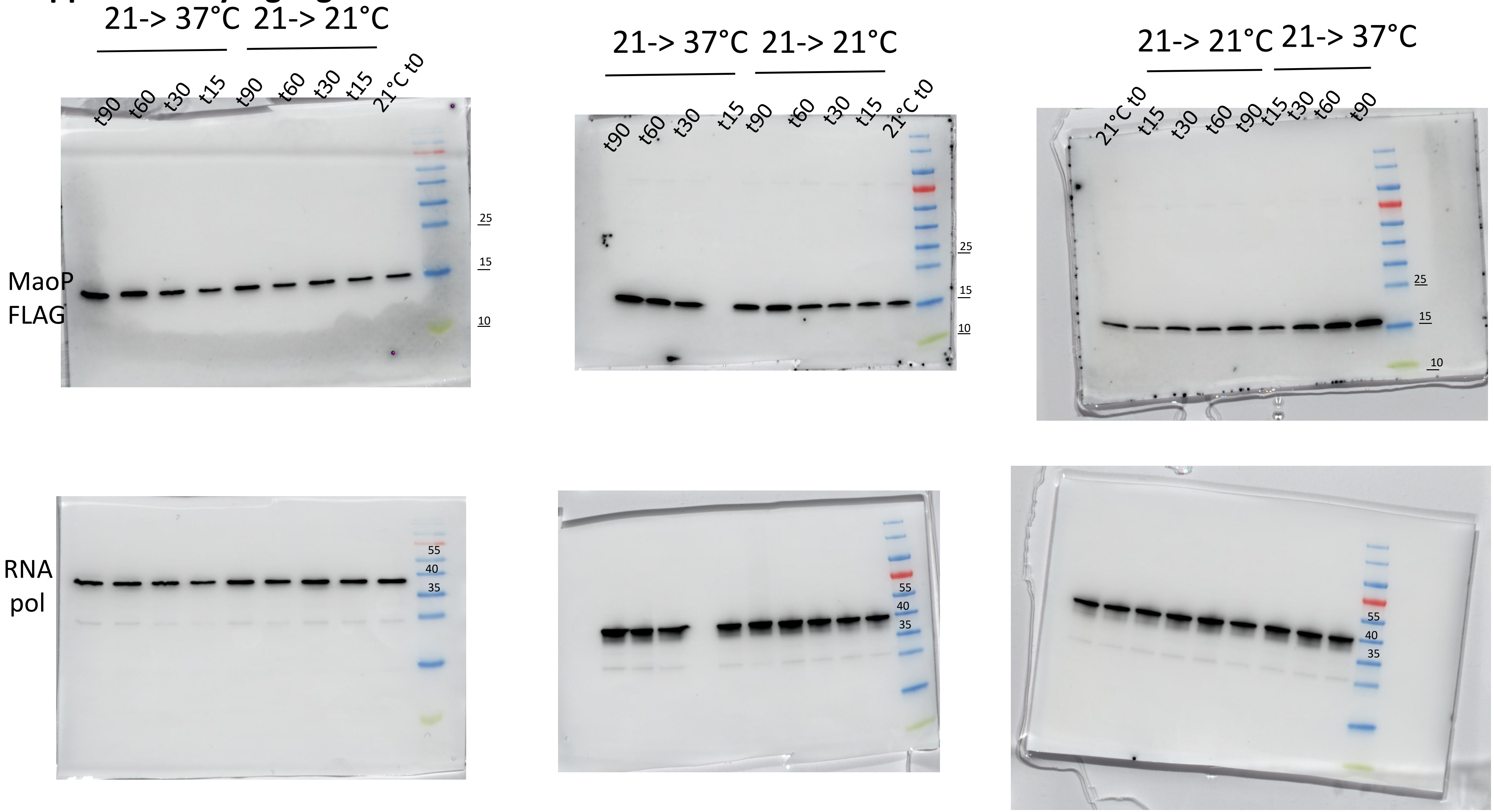

Supplementary Fig. 7c

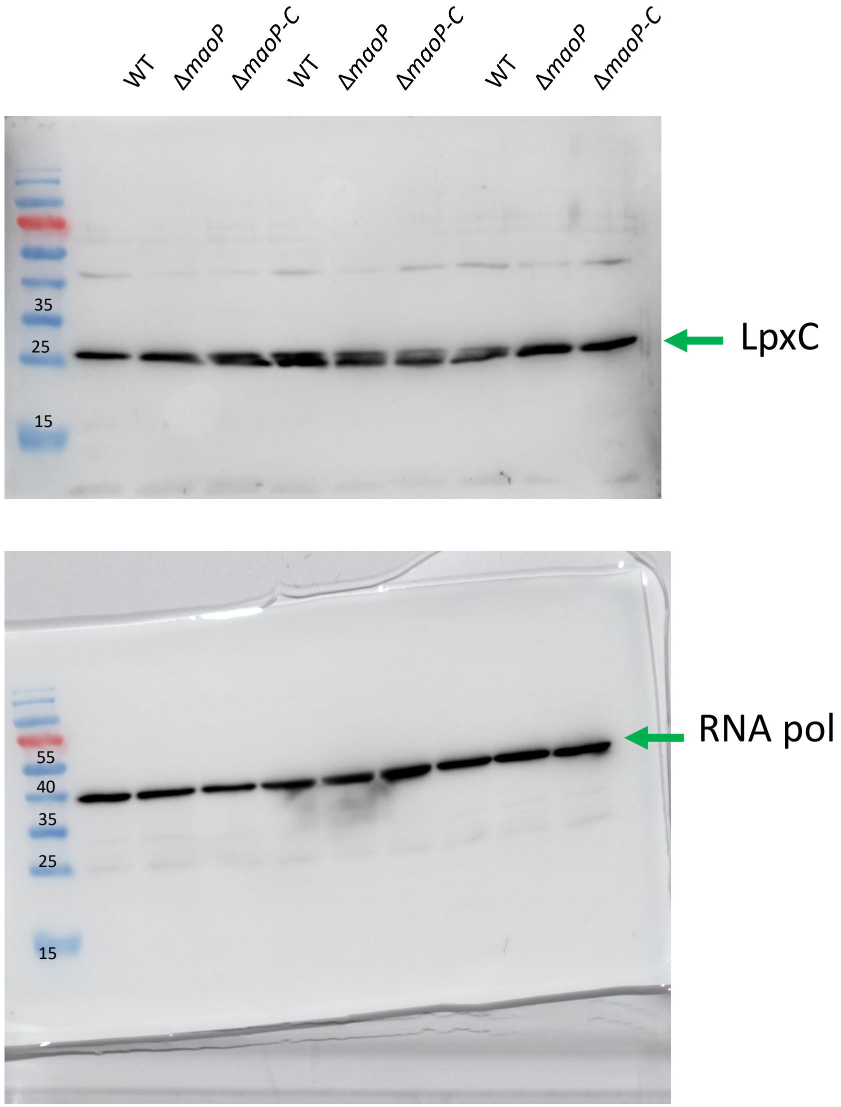

Supplementary Fig. 7d

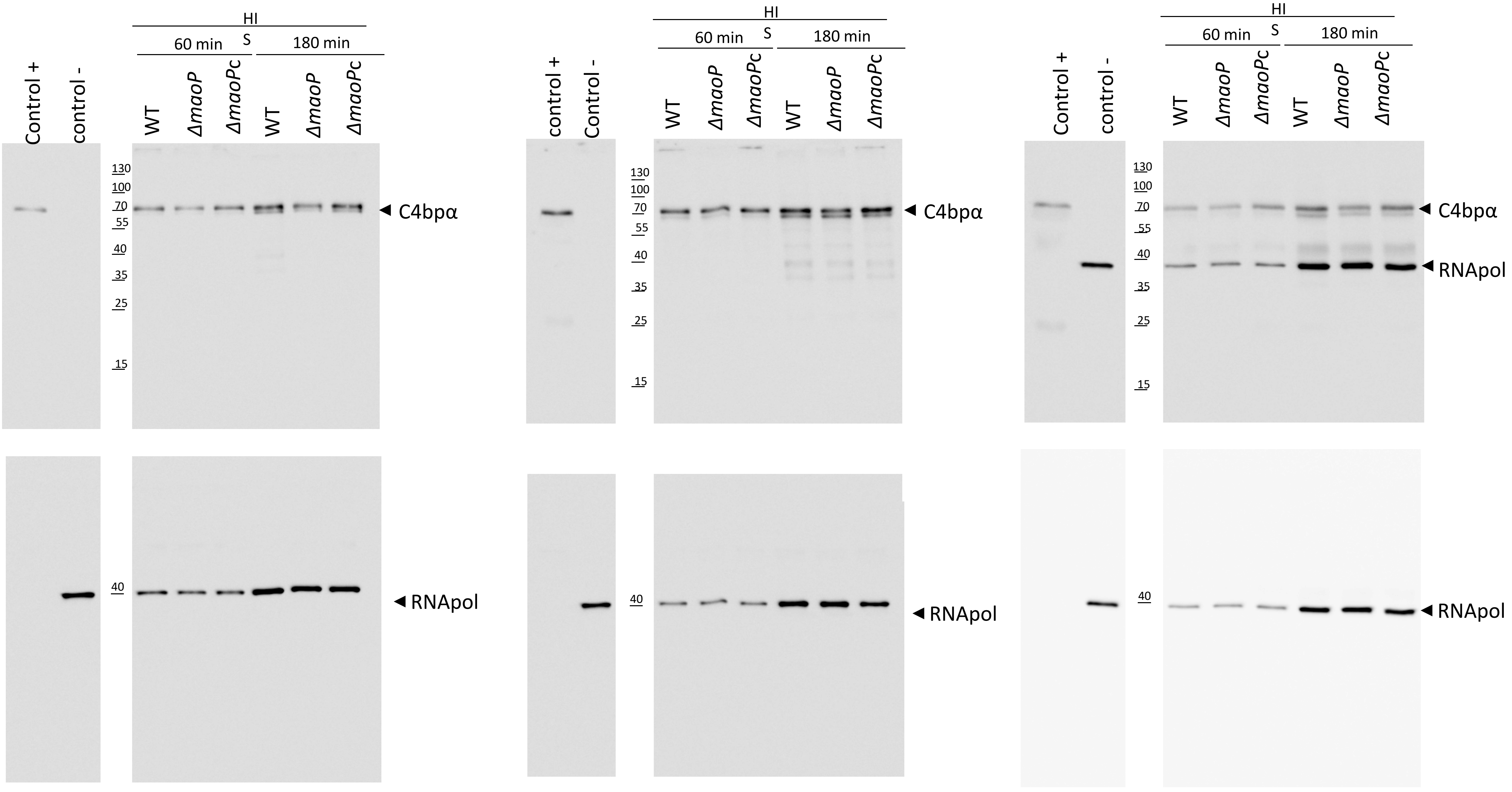

Supplementary Fig. 7d

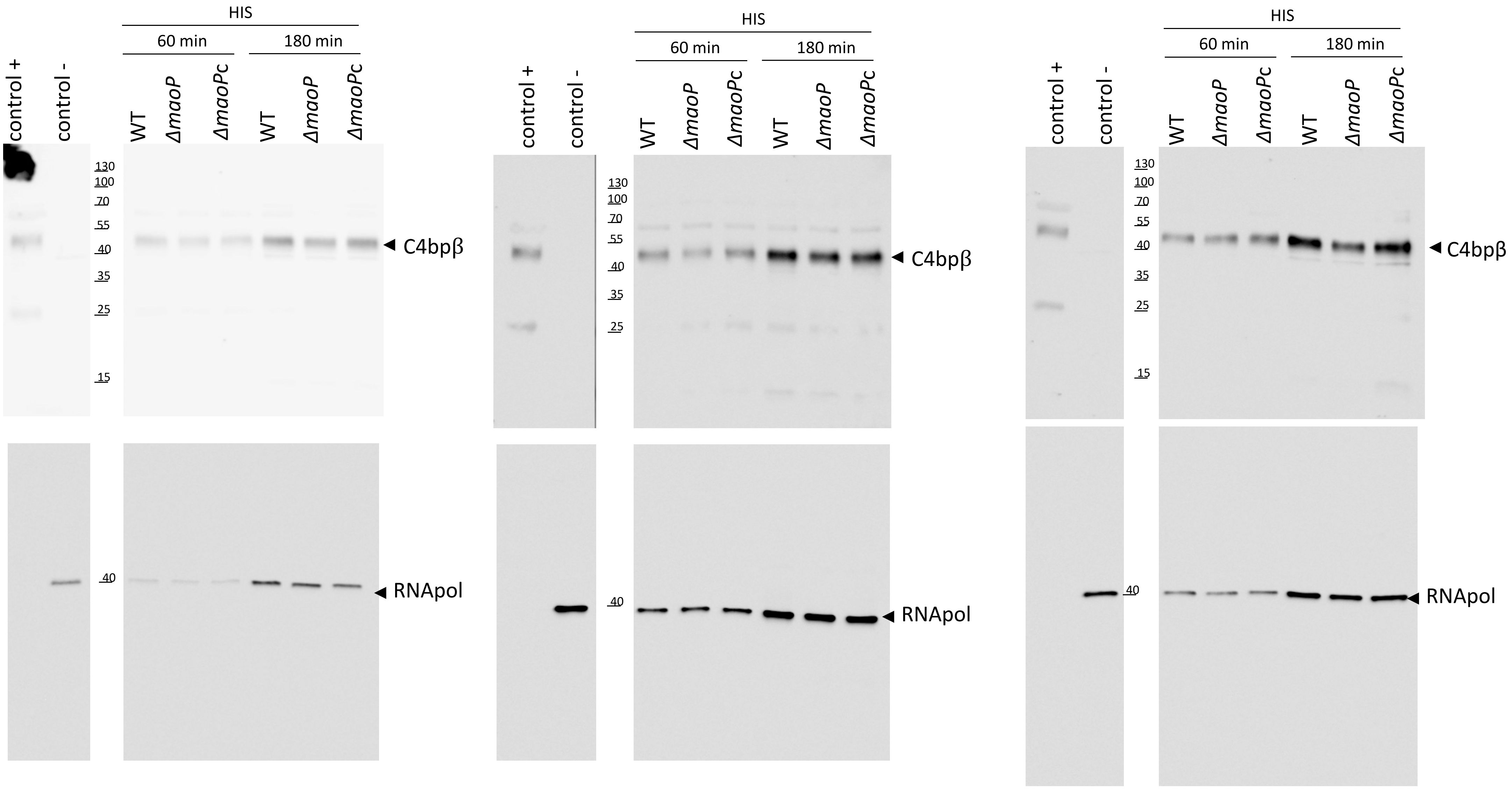

Supplementary Fig. 7d

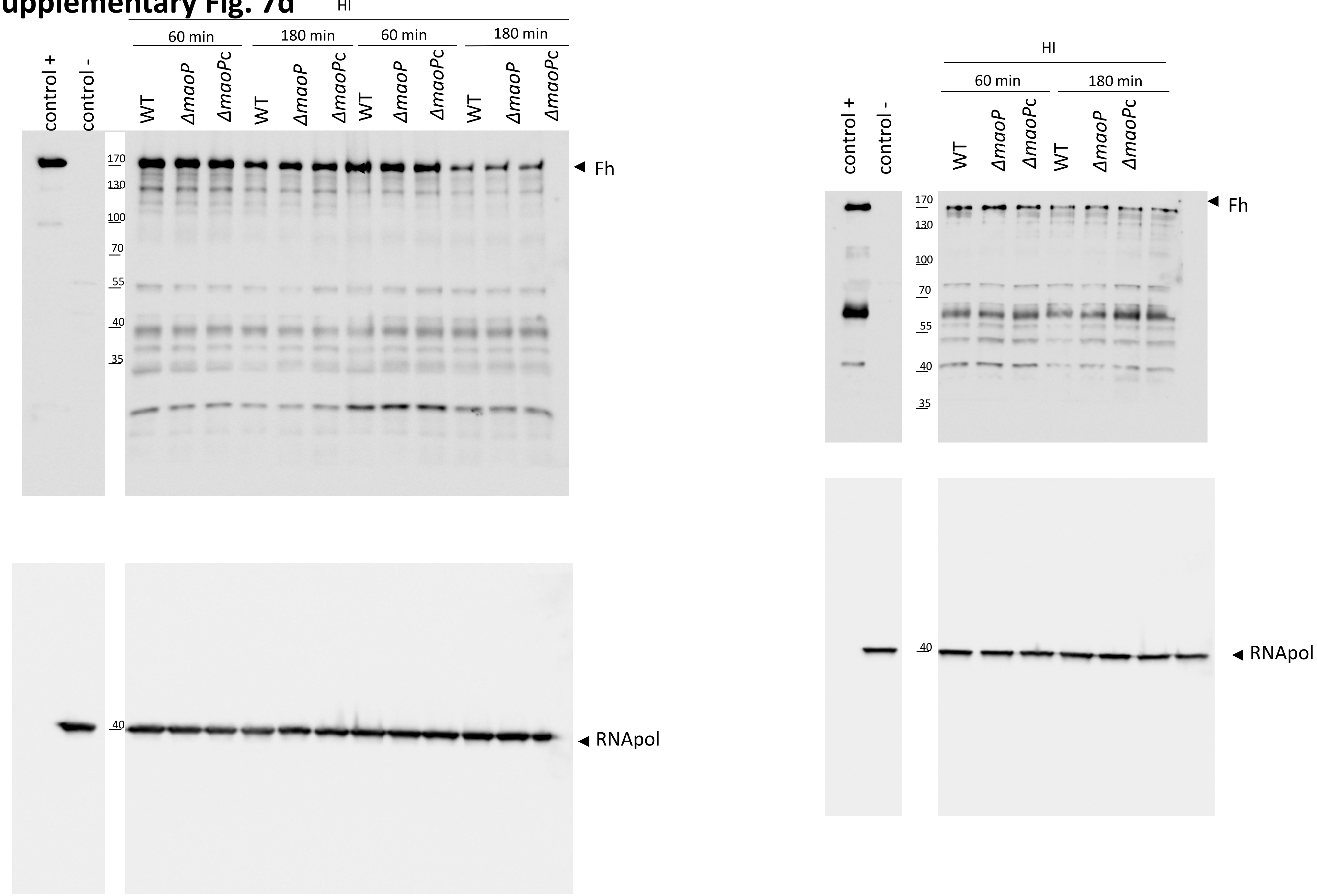

Supplementary Fig. 7d

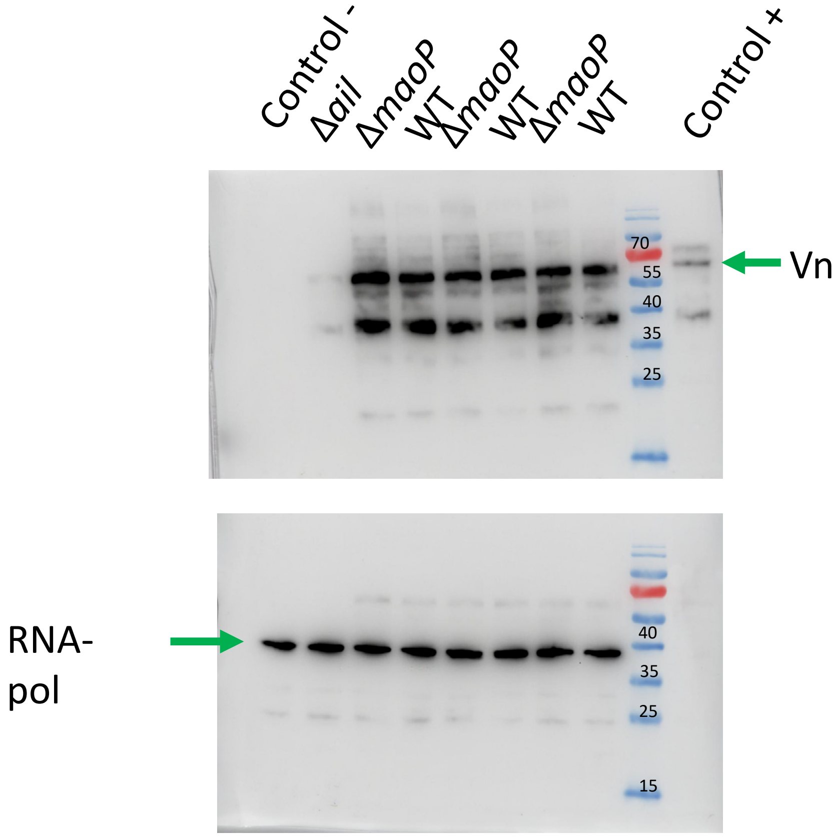

Supplementary Fig. 8

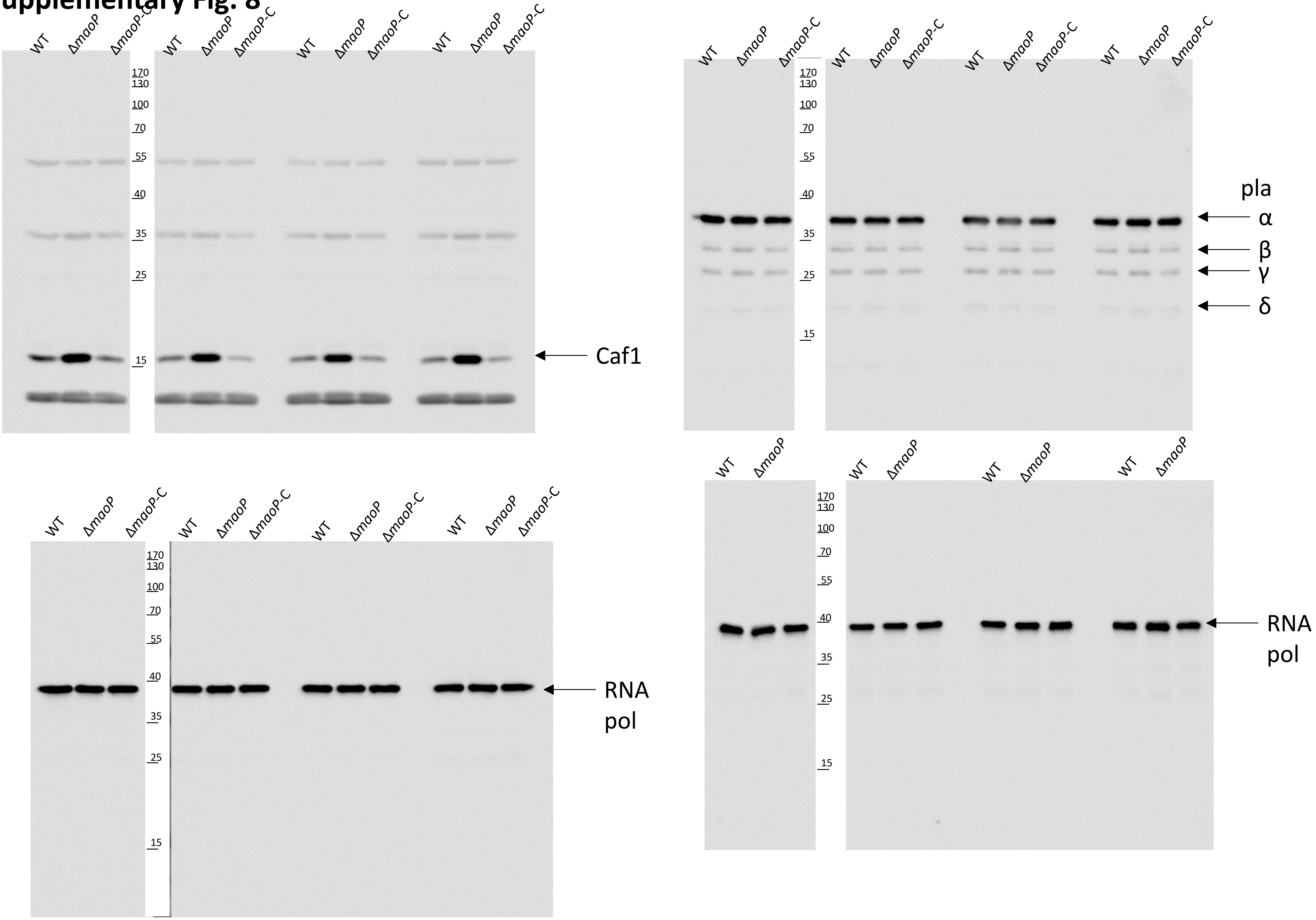

Supplementary Fig. 10b

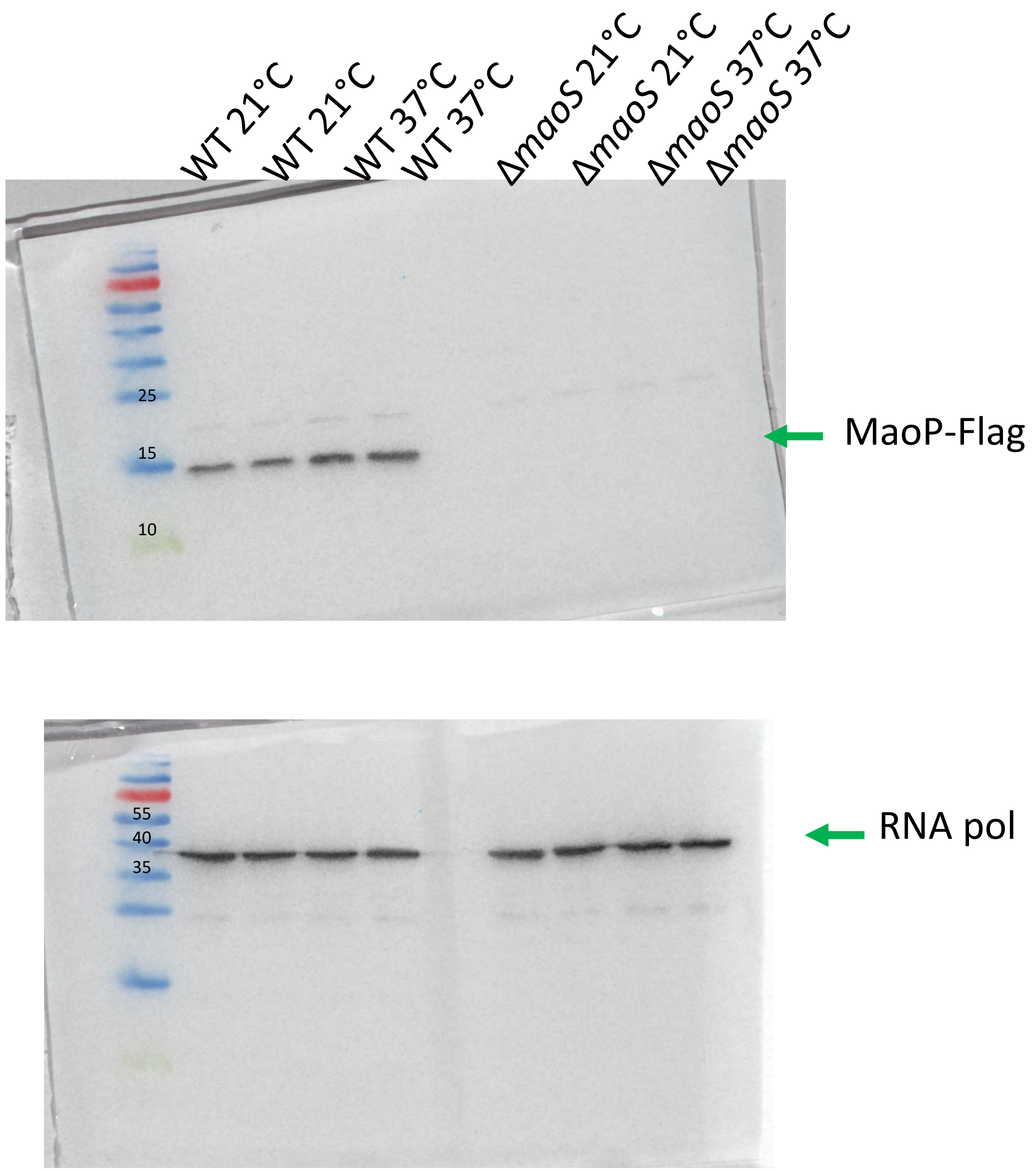

Supplementary Fig. 11B

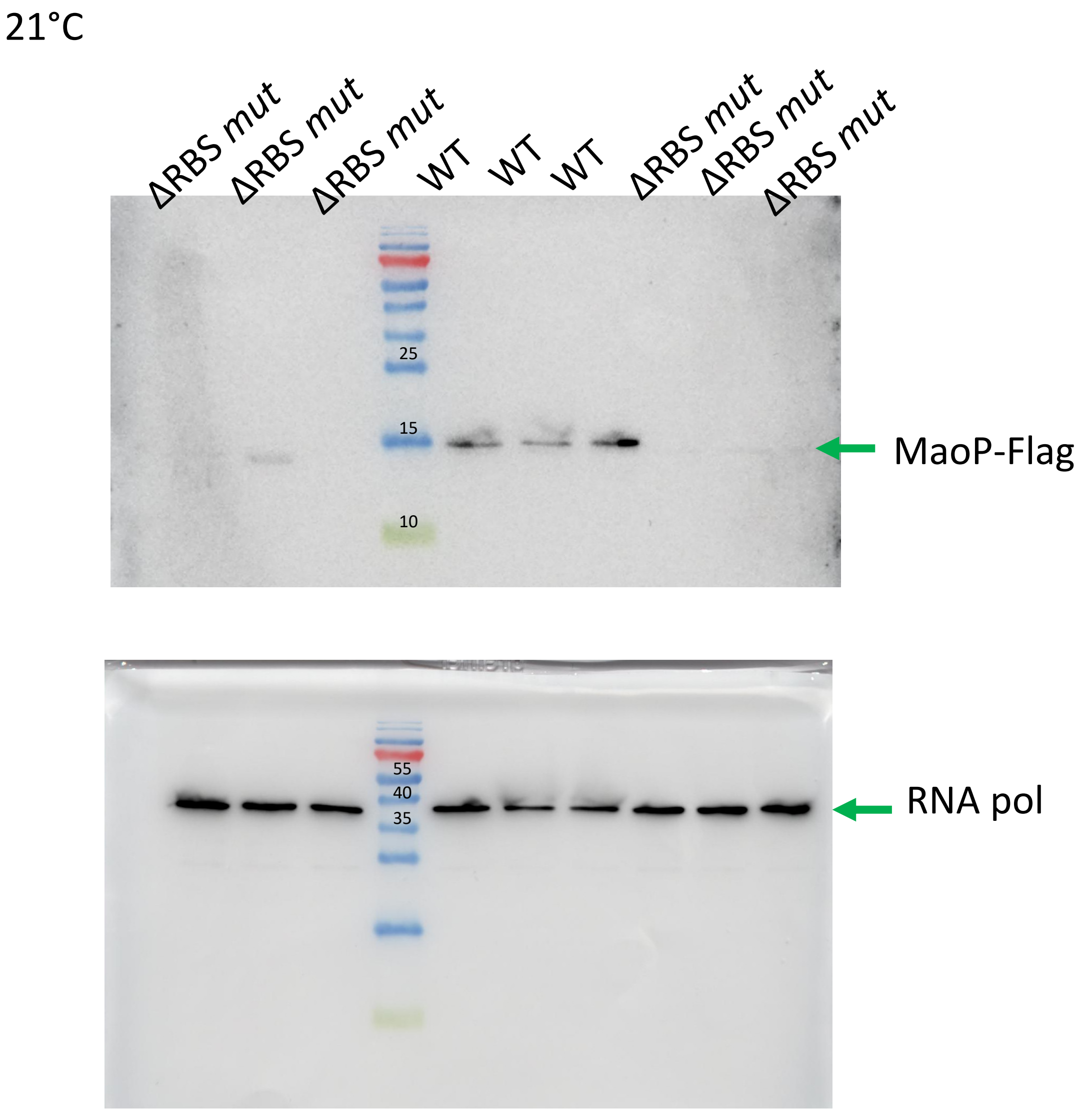

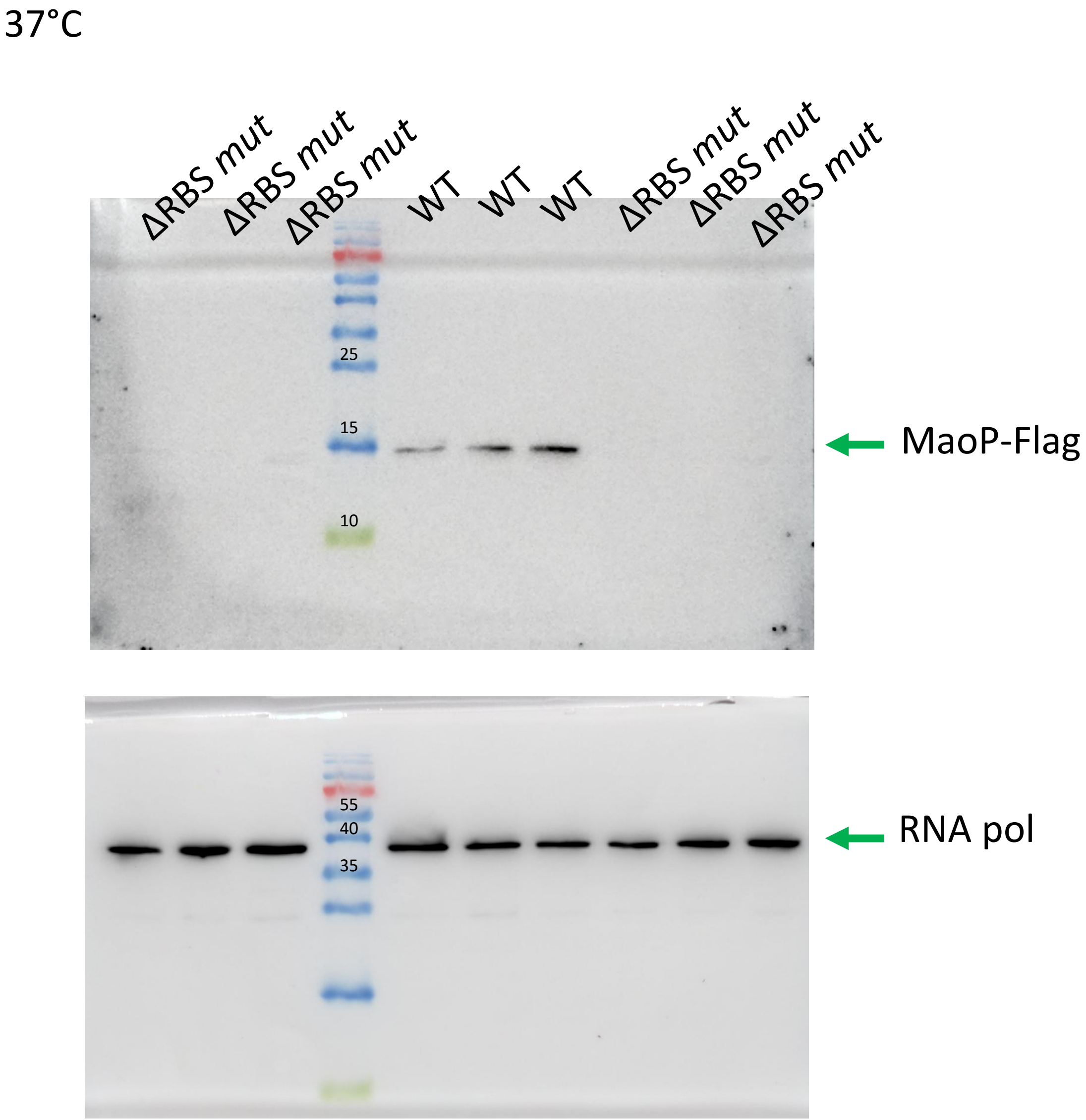

Supplement: Supplementary file 5 [file 41467_2026_77307_MOESM11_ESM.zip › Z_Gels and Blots_V2.pdf]
